# Evaluating the Effectiveness of Tethered Bis(urazolyl) Diradicals as Molecular Building Blocks for Dynamic Covalent Chemistry

**DOI:** 10.1021/acs.joc.3c00732

**Published:** 2023-06-26

**Authors:** Gary W. Breton, Kenneth L. Martin, James Alexander Bowron, John Bacsa

**Affiliations:** † Department of Chemistry, Berry College, Mount Berry, Georgia 30149 United States; ‡ Department of Chemistry, Emory University, Atlanta, Georgia 30322 United States

## Abstract

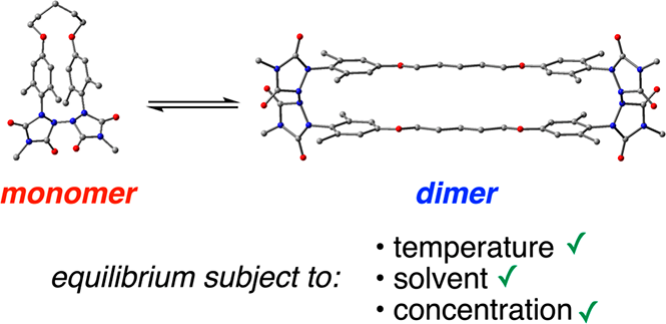

Dynamic covalent chemistry (DCvC) is a powerful means by which to rapidly prepare complex structures from simple molecular building blocks. Effective DCvC behavior is contingent upon the reversibility of covalent bond formation. Stabilized radical species, therefore, have been effectively used for these applications. In earlier work we demonstrated that properly substituted 1-arylurazolyl radicals showed promise as oxygen-insensitive heterocyclic N-centered radicals with a propensity for reversible bond formation. In this work we have synthesized several tethered bis(urazolyl) diradicals, varying by the type and length of connectivity between the urazole rings, and tested them for DCvC behavior. We have found that when the two aryl rings to which the urazolyl radical sites are attached are tethered by a chain of five or more carbons, equilibrium mixtures of monomeric and dimeric species are formed by N–N bond formation between two radical sites. DCvC behavior is observed that is sensitive to changes in temperature, concentration, and (to a lesser extent) solvent. In general, the dimer species is favored at lower temperatures and higher concentrations.

## Introduction

**Figure 1:**
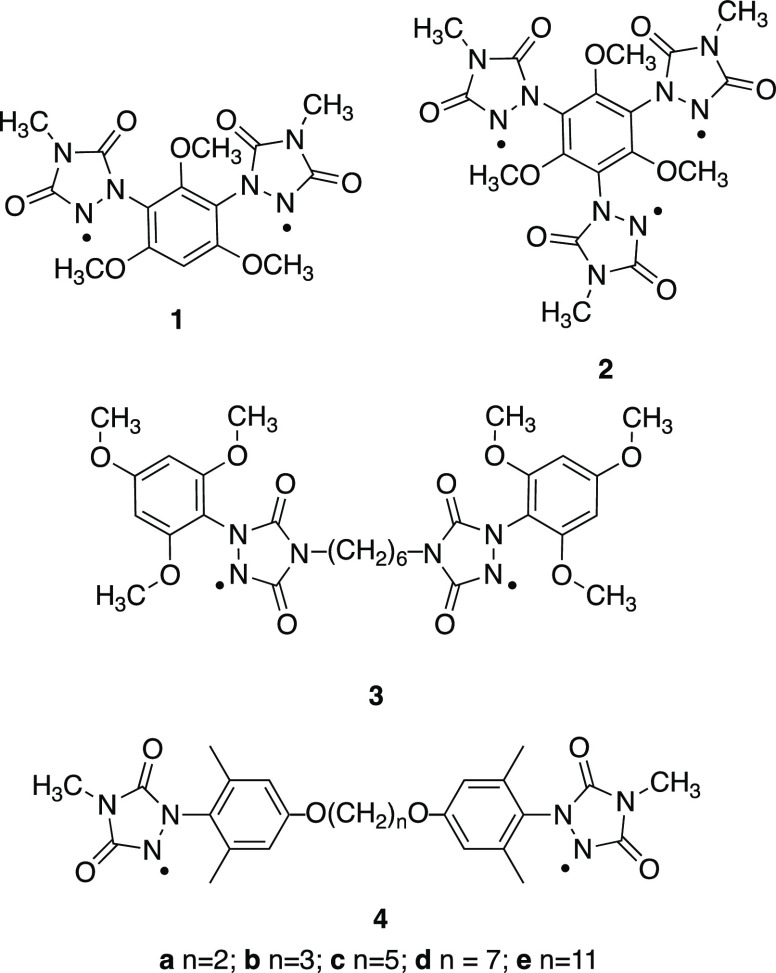
Structures of di- and triurazolyl radicals **1**–**4** tested for dynamic covalent chemistry behavior.

Dynamic covalent chemistry (DCvC) has emerged as a powerful means by which to rapidly prepare complex structures from simple molecular building blocks.^1−3^ The resulting structures have proven useful for a variety of applications, including those of materials science, catalysis, and biomedical sensing.^1−3^ A key aspect of DCvC that provides its strength as a synthetic method is the reversibility of covalent bond formation between the molecular building blocks. This reversibility allows the system to be responsive to environmental stimuli such as changes in temperature, concentration, pH, mechanical stress, and others.^1−3^ On the other hand, the requirement for the reversibility of bonding limits the variety of bonds that can be employed at practically accessible reaction temperatures and conditions. Imine, boronic acid ester, and disulfide bonds have proven especially robust.^1−3^ Radical species have also shown promise as molecular building blocks for DCvC applications.^4,5^ Indeed, we recently reported our findings on the behavior of bis- and tris(urazolyl) di- and triradicals **1** and **2**, respectively (Figure 1).^6^ Note that the bis-*ortho* substitution on the benzene rings to which the urazole rings are attached was determined to be a necessary structural requirement for the urazolyl radicals to prefer to form intermolecular N–N covalent bonds (required for DCvC applications) rather than to predominantly exist in radical form. While diradical **1** exhibited some borderline reversible behavior consistent with DCvC, triradical **2** did not, although in both cases interesting cage compounds were formed as major products. We suspected that structural restrictions imposed on these compounds from their proximate locations on the benzene rings may be the reason that their dynamic covalent behavior was stifled. To provide greater flexibility to the systems, we have now synthesized bis(urazolyl) diradicals **3** and **4** in which the radical urazole rings are tethered by a six-carbon alkyl chain and alkyl chains of varying lengths in **3** and **4**, respectively (Figure 1). Herein, we report our findings on the DCvC behavior of these interesting diradicaloid compounds.

## Results and Discussion

### Synthesis of Bis(urazole) Diradical Precursors

**Scheme 1:**
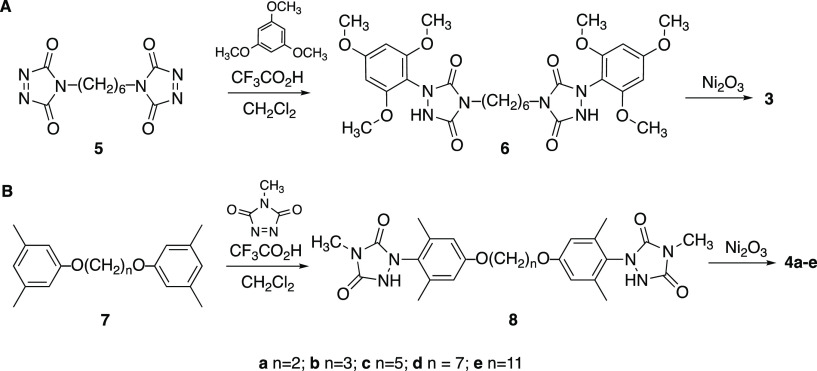
(A) Synthesis of Diradical Urazole Precursor **6** and Its Oxidation to Diradical **3** and (B) Synthesis of Diradical Urazole Precursors **8** and Their Oxidation to Diradicals **4a**–**e**

The bis(urazole) precursor to diradical **3**, compound **6**, was synthesized as outlined in Scheme 1A. Treatment of the known bistriazolinedione compound **5**(7) with 1,3,5-trimethoxybenzene in the presence of TFA as catalyst provided diurazole **6** in a 66% yield. In a similar fashion, the bis(urazole) precursors to diradicals **4** were synthesized as outlined in Scheme 1B. Tethered bis(ether) compounds **7** with varying methylene chain lengths were treated with the potent electrophile *N*-methyl-1,2,4-triazoline-3,5-dione (MeTAD) in CH_2_Cl_2_ in the presence of TFA to form diurazoles **8** in good yields. The corresponding diradicals were then generated via oxidation of the bis(urazoles) using the heterogeneous oxidant Ni_2_O_3_ as has been described previously.^6^

### Behavior of Bis(urazolyl) Diradical **3**

Treatment of a solution of diurazole **6** in CH_2_Cl_2_ with Ni_2_O_3_ initially turned the solution light purple in color, reminiscent of the deep blue color of simple aryl-substituted urazolyl radicals and indicative of the formation of radical species.^8^ After stirring for 4–5 h, the mixture was filtered to afford a yellow-brown solution that was concentrated to a brown plastic-like film. The loss of the purple color suggested that radical sites had been quenched, likely via the formation of inter- and or intramolecular N–N bonds.^6,9^ The ^1^H NMR spectrum of the crude product was complex and suggestive of the predominant oligomerization of diradicals **3** to form polymer chains of varying lengths. Indeed, TLC analysis in 100% ethyl acetate failed to budge the majority of the product mixture from the baseline, which was consistent with a very polar polymeric product. The initial formation of a plastic-like material had similarly been observed from reaction of diurazolyl radical **1**.^6^ However, heating of this plastic in boiling CHCl_3_ for 24 h was sufficient to convert the initially formed polymeric product to a single cage-like compound resulting from the dimerization of two of the diradical species.^6^ Unfortunately, however, heating a solution of the plastic formed from diradical **3** in CHCl_3_ failed to direct the system toward formation of discrete, characterizable products according to ^1^H NMR and TLC analyses.

We have demonstrated previously that thiophenol is a sufficiently strong hydrogen atom donor to quench urazolyl radicals.^6^ Addition of an excess of thiophenol to a solution of the oligomeric product in CDCl_3_ resulted in reaction within 2 h to form starting diurazole **6** (isolated in quantitative yield) in addition to the oxidized byproduct, diphenyl disulfide. This finding suggests that the N–N bonds formed in the reaction products are highly reversible and readily expose free radical sites to make them available for reduction by the thiophenol.

These initial results were discouraging, as it appeared that oligomeric products were favored over the formation of discrete monomeric, dimeric, or other types of products. Furthermore, it appeared that N–N bonds formed in the products were especially labile. We therefore abandoned the study of bis(urazolyl) diradicals tethered by connecting the N-4 nitrogen atoms of the urazole rings (i.e., **3**) in favor of bis(urazolyl) diradicals tethered via the N-1 substituted aromatic rings as in **4**.

### Behavior of Bis(urazolyl) Diradical **4a**

**Figure 2:**
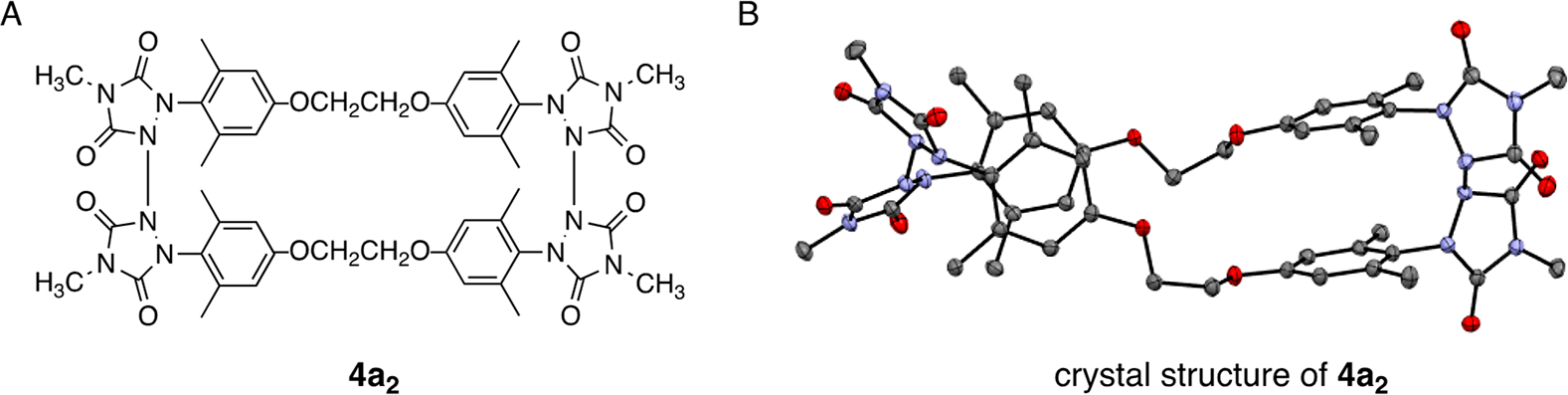
(A) Structure of diradical dimer **4a**_**2**_. (B) X-ray crystal structure of **4a**_**2**_ with thermal elipsoids set at 50% probability. Hydrogen atoms have been hidden to enhance visual clarity.

Bis(urazole) **8a** had very low solubility in CH_2_Cl_2_, which prevented efficient oxidation using Ni_2_O_3_ at room temperature. Therefore, a mixture of **8a** and Ni_2_O_3_ in CHCl_3_ was refluxed for 3 h, then cooled, and filtered. The ^1^H NMR spectrum indicated formation of a single major product, which was isolated via column chromatography in 63% yield. Prolonged heating of the reaction mixture in the presence of Ni_2_O_3_ (or an independently prepared mixture of purified product and Ni_2_O_3_) led to degradation of the product, although heating the product alone as a solution in CHCl_3_ (i.e., in the absence of Ni_2_O_3_) for 24 h did not affect the product. Interestingly, the ^1^H NMR spectrum revealed a single N-Me signal, but a set of doublets for the aromatic ring protons (δ 6.6 and 6.2 ppm), a set of doublets for the methylene protons (δ 4.3 and 4.2 ppm), and two signals for the aryl CH_3_ protons (2.3 and 1.7 ppm). In the ^13^C NMR spectrum, 6 different aromatic ring carbons were observed as well as two different benzylic CH_3_ carbons, but a single signal for both methylene carbons. This data suggested that the 2-carbon methylene chains were likely constrained in a conformation that rendered the geminal protons inequivalent. The high-resolution mass spectrum (HRMS) was consistent with a dimer-type structure in which the ends of two different diradicals **4a** had been joined via N–N bond formation as in the dimer in Figure 2A, designated as **4a**_**2**_. Fortunately, we were able to confirm the structure of the molecule via X-ray crystallography (Figure 2B). The twisted nature of the dimer in the crystal structure may reflect the symmetry-breaking conformation adopted in solution that results in multiple chemical environments in the ^1^H NMR spectrum, whereas the nominal structure (Figure 2A) suggests identical chemical environments.

The N–N bonds joining the urazole rings had bond lengths (1.39 Å) that were even slightly shorter than the N–N bonds within the urazole rings (1.44 Å), suggesting that they were not particularly strained. Indeed, treating either a freshly filtered mixture of oxidized **8a**, or a sample of isolated and purified **4a**_**2**_, with an excess of thiophenol again led to clean formation of the starting bis(urazole) **8a** (68% isolated yield), but the process took over a week to complete. Thus, the N–N bonds in **4a**_**2**_ were apparently less prone to opening to expose the nitrogen-centered radical than what had been observed for reaction mixtures of diradical **3**.

### Behavior of Bis(urazolyl) Diradical **4b**

**Figure 3:**
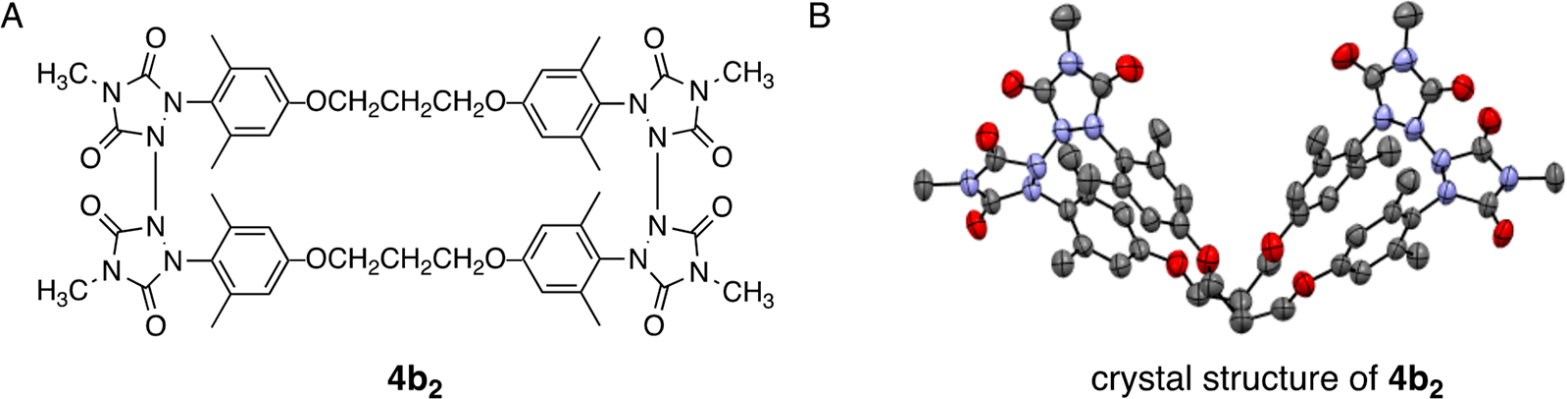
(A) Structure of diradical dimer **4b**_**2**_. (B) X-ray crystal structure of **4b**_**2**_ with thermal elipsoids set at 50% probability. Hydrogen atoms have been hidden to enhance visual clarity.

Unlike bis(urazole) **8a**, bis(urazole) **8b** was soluble in chlorinated solvents. Thus, oxidation of **8b** with Ni_2_O_3_ could be performed at room temperature within an hour’s time. Concentration of the resulting solution, after filtering and washing the heterogeneous catalyst, afforded a colorless plastic-like film. ^1^H NMR and TLC analysis suggested the formation of a single major product along with several minor products that were not identified. The major product could be isolated via column chromatography as a white solid in a 64% yield. The ^1^H and ^13^C NMR spectra of the product displayed an asymmetry analogous to that of dimer **4a**_**2**_ discussed above, suggesting a similar structure. HRMS confirmed the molecular formula to be consistent with that of dimer **4b**_**2**_ (Figure 3A). The NMR spectra were complicated by the presence of a nearly identical number of much smaller signals located just adjacent to the expected major signals for the compound in about a 5:1 ratio. While these smaller signals were at first taken to be those of an impurity, we found that they reversibly coalesced with the larger signals upon heating the sample in the probe of an NMR spectrometer. Hence, we believe they represent the signals of a minor, slowly interconverting, conformer. As observed for dimer **4a**_**2**_, heating a CDCl_3_ solution of dimer **4b**_**2**_ to reflux for 24 h had no effect on the dimer. Finally, we were able to isolate crystals of this compound suitable for X-ray crystallography to confirm our structure assignment (Figure 3B).

Treatment of a sample of **4b**_**2**_ with an excess of thiophenol resulted in clean reduction of the dimer back to bis(urazole) **8b** (identified by ^1^H NMR spectroscopy), which could be isolated in 82% yield, over a four-day period.

### Behavior of Bis(urazolyl) Diradical **4c**

**Figure 4:**
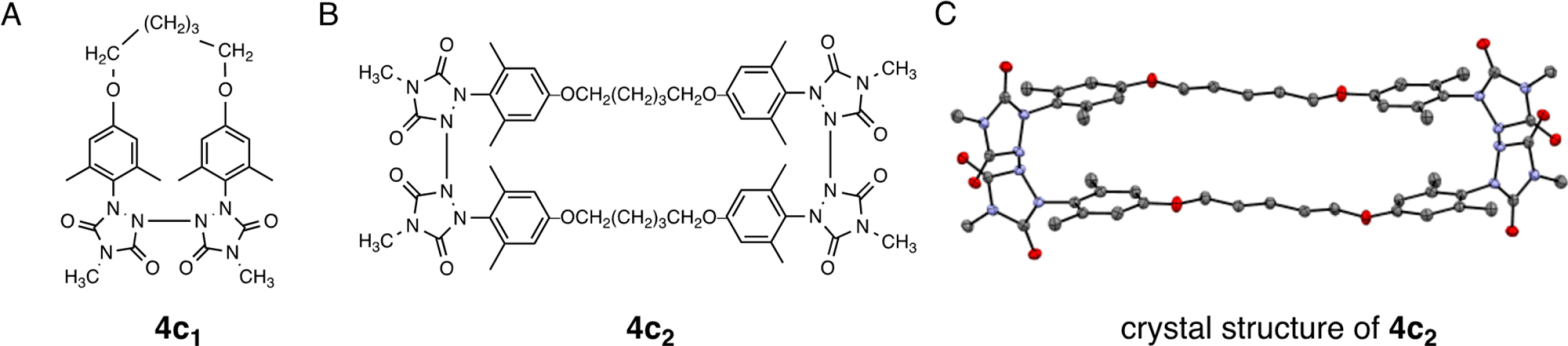
(A) Structure of monomer **4c**_**1**_. (B) Structure of diradical dimer **4c**_**2**_. (C) X-ray crystal structure of **4c**_**2**_ with thermal elipsoids set at 50% probability. Hydrogen atoms have been hidden to enhance visual clarity.

Bis(urazole) **8c** was sufficiently soluble in chlorinated solvents to allow for room temperature oxidation. Thus, oxidation of **8b** with Ni_2_O_3_ in CH_2_Cl_2_ at rt for 1 h afforded, after filtration, a white solid. TLC analysis revealed the formation of two products. The products could be separated via column chromatography to afford two compounds in approximately a 3:1 mass ratio (comprising ∼90% of the starting mass), with the minor component being the less polar. NMR spectroscopic analysis and high-resolution mass spectrometry identified the minor component as a monomer, designated as **4c**_**1**_, and the major component as dimer **4c**_**2**_ (see Figure 4). The ^1^H and ^13^C NMR spectra of the dimer indicated the presence of two conformations in unequal amounts in solution (in an approximate 2:1 ratio) as discussed earlier for **4b**_**2**_. Diffusion-ordered NMR spectroscopy (DOSY) conducted on a mixture of the two compounds clearly separated key signals of the faster diffusing monomer **4c**_**1**_ from those of the slower diffusing dimer **4c**_**2**_ (see the Supporting Information). We were fortunate to be able to crystallize dimer **4c**_**2**_ and perform X-ray crystal analysis to confirm its structure (see Figure 4C). Unlike **4a**_**2**_ and **4b**_**2**_, the longer alkyl chain of **4c_2_** enforces a nearly linear geometry upon the dimer’s structure, at least in the crystal lattice. The N–N bonds joining the two urazole rings remain at 1.39 Å as observed for the other two dimers (1.39 and 1.38 Å, respectively). Unfortunately, the monomer eluded out efforts to form crystals of sufficient purity for X-ray analysis.

**Figure 5:**
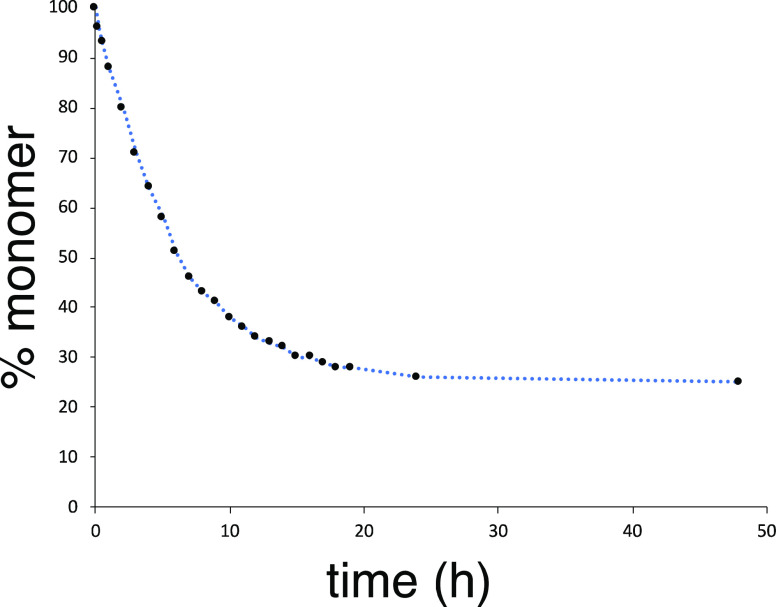
Equilibration of monomer **4c**_**1**_ with dimer **4c**_**2**_ in CDCl_3_ solution as monitored by ^1^H NMR spectroscopy.

For both the purified monomer and dimer, if left in solution, an equilibrium mixture of the two compounds was re-established over the course of ∼24 h. Figure 5 traces this equilibration process starting with a sample of pure monomer. When thiophenol was added to a solution of the monomer **4c**_**1**_ prior to allowing it time to equilibrate, only tiny amounts of the dimer were observed to form. Instead, clean reduction to bis(urazole) **8c** took place and conversion was complete within 7 h. Similarly, when thiophenol was added to a solution of dimer **4c**_**2**_, clean reduction to the bis(urazole) took place, but the process took nearly four days to complete. The bis(urazole) was isolated in an 83% yield.

We tested the response of the monomer/dimer equilibrium to a variety of external stimuli, including change in solvent polarity, concentration, and temperature. The ratio of dimer **4c**_**2**_ to monomer **4c**_**1**_ in each case was determined from integrations of well-separated signals corresponding to each species in the ^1^H NMR spectrum.

#### Solvent Polarity Dependence

Equivalent concentrations of a mixture of monomer and dimer were allowed to equilibrate for 24 h in solvents of increasing polarity, and the final ratio of dimer to monomer was determined by relative integrations (**4c**_**2**_/**4c**_**1**_): C_6_D_6_, 66:34; CDCl_3_, 68:32; and (CD_3_)_2_SO, 69:31. Unfortunately, the compound was insoluble in CD_3_OH, so we could not determine whether potential hydrogen bonding effects would have affected the equilibrium. While there was a slight correlation of the amount of dimer present relative to monomer with increasing solvent polarity, the variance was small and likely within the limits of error.

#### Concentration Dependence

Solutions of varying initial concentrations of mixtures of monomer/dimer in CDCl_3_ were allowed to equilibrate at room temperature for at least 24 h before analysis (**4c**_**2**_/**4c**_**1**_): 0.5 mM, 58:42; 1.0 mM, 59:41; 2 mM, 60:40; 4 mM, 61:39; 8 mM, 67:33; 16 mM, 74:26; 32 mM, 81:19; and 64 mM, 85:15. Thus, higher concentrations greatly favored formation of the dimer species, consistent with the greater opportunity for diradical species of **4b** to encounter one another.

#### Temperature Dependence

**Figure 6:**
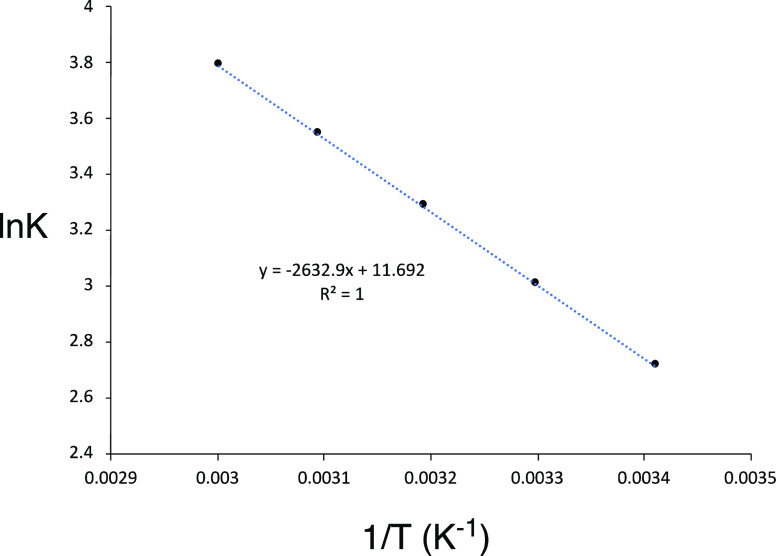
Linear dependence of ln *K* for dimer/monomer equilibrium on 1/*T* as measured by ^1^H NMR spectroscopy of equilibrated solutions at various temperatures.

A solution of a mixture of monomer and dimer in DMSO–D_6_ was sealed under vacuum in an NMR tube. The tube was equilibrated in a mineral oil bath at various temperatures for 24 h prior to analysis (**4c**_**2**_/**4c_1_**): 20 °C, 68:32; 30 °C, 64:36; 40 °C, 60:40; 50 °C, 56:44; and 60 °C, 52:48. At temperatures of 70 °C and above, the compounds degraded, most noticeably toward the formation of bis(urazole) **8c**. The increase in the amount of monomer with higher temperatures is consistent with the increasing importance of the entropic advantage of forming the monomer. A linear Van’t Hoff plot of the dependence of ln *K* versus 1/*T* provided Δ*H* = +5.2 kcal/mol and Δ*S* = +23.2 cal/mol·K for the dimer-to-monomer conversion (see Figure 6).

**Figure 7:**
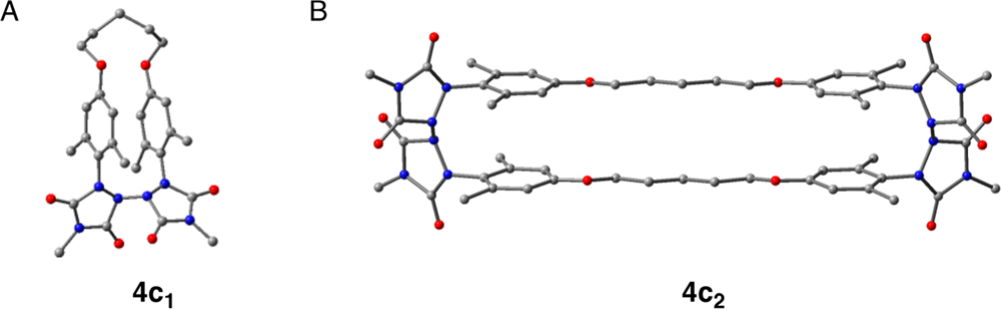
(A) Computationally obtained structure for C2-symmetric monomer **4c**_**1**_. (B) Computationally obtained structure for *C*_*i*_-symmetric dimer **4c**_**2**_. Hydrogen atoms have been hidden to enhance visual clarity.

We optimized the geometry of dimer **4c**_**2**_ computationally using the DFT functional ωB97X-D (which includes dispersion effects) in conjunction with the 6-31G* basis set (see Figure 7B). We were able to use the X-ray crystal structure coordinates as an initial guess for the structure of **4c**_**2**_, although we enforced *C*_*i*_ symmetry on the system (the symmetry classification closest to the X-ray derived geometry). In the absence of a starting geometry for monomer **4c**_**1**_, we conducted a conformation search and landed upon the *C*_2_-symmetric structure shown in Figure 7A. The change in enthalpy for the dimer-to-monomer conversion was calculated to be +13.4 kcal/mol, which is in qualitative agreement with the +5.2 value obtained experimentally. The change in entropy was calculated to be +29.9 cal/mol·K, in good agreement with the experimental value (+23.2 cal/mol·K). Thus, both experimental and computational results agree that the dimer is favored enthalpically but the monomer is favored entropically. The positive entropic change for the reaction is consistent with the conversion of a single dimer unit into two monomers. To identify the reason for the positive change in enthalpy, we carefully analyzed the optimized structures of the monomer and dimer. The N–N bonds joining the urazole rings in both **4c**_**2**_ and **4c**_**1**_ were essentially identical in length (1.37 Å) indicating that one set of bonds was not under any substantial strain relative to the other. Additionally, the distances between the stacked benzene rings in both cases were similar (3.73 Å in the dimer versus 3.78 Å in the monomer), as well as the distances between the two oxygen atoms in the tethering chains (4.00 versus 4.08 Å for **4c**_**2**_ and **4c**_**1**_, respectively). However, one significant difference in structures was noted between the dimer and monomer that would impact relative stabilities. It was noted in the structure of **4c**_**2**_ that the C–C bonds of the five-carbon tethering chain are able to adopt antistaggered conformations throughout, thereby minimizing steric strain. The structure of **4c**_**1**_, on the other hand, requires that the C–C bonds exist in gauche conformations that would gradually accumulate strain energy. Indeed, assuming the penalty for assuming a gauche conformation is similar to that for butane, the four such interactions at 0.6 kcal/mol apiece provide a total strain energy of ∼5 kcal/mol (for two monomers), which is consistent with the Δ*H* value measured experimentally (+5.2 kcal/mol).^10^ Therefore, it is likely the added strain imposed by these gauche conformational interactions that provides the enthalpic advantage to the dimer.

### Behaviors of Bis(urazolyl) Diradicals **4d** and **4e**

The behaviors of two additional bis(urazolyl) diradicals, **4d** and **4e**, were investigated. These two diradicals had 7-carbon and 11-carbon tethering chains, respectively. Both of the parent bis(urazoles) were soluble in CDCl_3_, which allowed for their oxidation at room temperature. The 7-carbon-tethered bis(urazole) **8d** afforded a mixture of monomer **4d**_**1**_ (21% yield) and dimer **4d**_**2**_ (56% yield) upon oxidation in a manner similar to that found for the 5-carbon bis(urazole) **8c**. The dimer **4d**_**2**_ again exhibited signals for two conformations in solution, this time in an approximate 1.5:1 ratio. If the purified compounds remained in solution, both the isolated monomer and dimer re-established an equilibrium mixture within 24 h. Treatment of a mixture of the two compounds with thiophenol resulted in clean reduction back to the starting urazole **8d** over a 24 h period. Oxidation of the 11-carbon-tethered bis(urazole) **8e** proceeded similarly to that of **8d**. Both a monomer **4e**_**1**_ (12% yield) and a dimer **4e**_**2**_ (36% yield) were isolated. Initial examination of the ^1^H NMR spectrum of the dimer suggested that only a single conformer might be present, unlike several of the other dimers. However, careful examination of the ^13^C NMR spectrum, especially upon spectral processing in the absence of line broadening, revealed that most carbons were actually twins of nearly equivalent intensity, suggesting that an approximate 1:1 ratio of conformers existed in solution. The DOSY spectrum, however, separated only two species, a faster migrating compound with signals corresponding to those of the monomer and a slower migrating species with signals corresponding to those of the dimer. Thus, in the series of dimers **4b**_**2**_, **4c**_**2**_, **4d**_**2**_, and **4e**_**2**_, there was a regular progression of conformational isomer mixtures from (major/minor) 5:1, 3:1, and 1.5:1 to 1:1, respectively.

Note that the total yield of monomer and dimer obtained upon room-temperature oxidation of bis(urazole)s **8c**, **8d**, and **8e** decreased (93%, 67%, and 48% total yields, respectively) with the increasing length of the carbon chain tether. This is likely due to the formation of increasing amounts of very polar oligomeric products competing with the formation of the two major products. Longer chains are undoubtedly more likely to form oligomers due to the decreased probability of the two nitrogen radicals finding one another to form the discrete products.

## Conclusions

Diurazolyl diradicals tethered via the N-4 nitrogen of the urazole ring as in **3** appear to be a poor choice for providing discrete products, at least under the reaction conditions investigated. Instead, random oligomerization is the preferred mode of N–N connectivity of the radical sites. On the other hand, tethering via phenoxy rings as in **4** appears to be more promising. Short tethering chains (i.e., 2- and 3-carbon, respectively) as in **4a** and **4b** form single dimeric products similar to what had been previously reported for diradicals **1** and **2**. However, longer chain lengths (i.e, 5, 7, and 11, respectively) as in **4c**, **4d**, and **4e** provide two major discrete products, a monomer and a dimer. These products are able to interconvert in solution in a reasonable amount of time (∼24 h) in a dynamic process via reversible N–N bond formation of the urazolyl radical sites. However, they are sufficiently stable to be able to withstand chromatographic purification and characterization. Based on what was learned from the behavior of **4c**, the dimer **4c**_**2**_ is favored at lower temperatures due to an enthalpic advantage over **4c**_**1**_. The dimer is also favored at higher concentrations, as the probability of two diradicals encountering one another is increased. At higher temperatures, however, the equilibrium is shifted toward the monomer due to entropic contributions. Solvent effects on the equilibration process appear to be minimal. Thus, these studies suggest that properly substituted 1-aryl urazolyl radicals can serve as a novel means by which to design molecular building blocks with promising applications in dynamic covalent chemistry.

## Experimental Section

### General Methods

Column chromatography was conducted on silica gel (234–400 mesh). Thin-layer chromatography was performed on precoated silica gel plates (250 mm) and visualized by ultraviolet light. ^1^H and ^13^C{^1^H} NMR spectra were obtained on a 400 MHz NMR spectrometer. Chemical shifts are reported in units of parts per million downfield from TMS. High-resolution mass spectra (HRMS) were acquired via electron spray ionization on an LTQ-FTMS hybrid mass spectrometer. *N*-Methyl-1,3,5-triazoline-3,5-dione (**2**) was synthesized via oxidation of *N*-methylurazole with DABCO-Br_2_ as described in the literature.^11,12^ Bistriazolinedione **5** was synthesized according to the literature procedure.^7^ All other chemicals and solvents were obtained from commercial sources and used without further purification unless otherwise noted.

#### Bisurazole **6**

To a solution of 0.46 g (1.64 mmol) of bistriazolinedione **5**(7) in 55 mL of CH_2_Cl_2_ was added 0.54 g (3.28 mmol, 2 equiv) of trimethoxybenzene, followed by 250 μL (3.28 mmol) of CF_3_CO_2_H via syringe. The red color of **5** was discharged over 24 h. The reaction mixture was concentrated in vacuo, taken up in 20 mL of CH_2_Cl_2_, and washed with 20 mL of 1 M aq. NaOH. The aqueous layer was washed with 20 mL of fresh CH_2_Cl_2_ and then acidified with conc. HCl to pH = 2. The aqueous layer was then washed 2 × 50 mL CH_2_Cl_2_. The combined organic layers were dried over Na_2_SO_4_, filtered, and concentrated to afford 0.64 g (63% yield) of bis(urazole) **6** as a white solid, mp 239–240 °C: ^1^H NMR (400 MHz, DMSO-*d*_6_) δ 10.58 (s, 2H), 6.30 (s, 4H), 3.81 (s, 6H), 3.74 (s, 12H), 3.43 (t, *J* = 6.7 Hz, 4H), 1.58 (br m, 4H), 1.32 (br m, 4H); ^13^C{^1^H} NMR (100 MHz, DMSO-*d*_6_) 162.2, 158.8, 153.7, 153.5, 106.2, 91.3, 56.2, 55.8, 38.4, 27.6, 25.6. HRMS (ESI) *m*/*z* [M + H]^+^ Calcd for C_28_H_37_N_6_O_10_ 617.2566, found 617.2570.

#### Oxidation of Bisurazole **6**

To a solution of 62 mg (0.1 mmol) of bis(urazole) **6** in 2 mL of CH_2_Cl_2_ were added 100 mg (0.7 mmol) of Na_2_SO_4_ and 120 mg (0.4 mmol) of Ni_2_O_3_ (30% activity) with stirring. The solution turned pale purple in color. The mixture was stirred for 4 h and then filtered through a fine glass fritted funnel under N_2_ pressure to remove insolubles. The filtrate was concentrated to 58 mg of a plastic-like film. TLC analysis (100% EtOAc) showed several very light (under UV) mobile spots but indicated the majority of the material remained at the baseline. The ^1^H NMR spectrum was complicated with very broad signals (see the Supporting Information).

The material was taken up in 2 mL of CDCl_3_ in a 10 mL RBF to which was fitted a reflux condenser and drying tube. The solution was then heated to reflux using a heating mantle for 24 h, cooled, and reconcentrated. TLC and ^1^H NMR analysis showed no noticeable change from the mixture prior to heating.

#### Reaction of Oxidized Mixture of Bisurazole **6** with Thiophenol

To a solution of 23 mg (0.1 mmol) of bis(urazole) **6** in 1.5 mL of CH_2_Cl_2_ were added 50 mg (0.4 mmol) of Na_2_SO_4_ and 45 mg (0.4 mmol) of Ni_2_O_3_ (30% activity) with stirring. The solution turned pale purple in color. The mixture was stirred for 5 h and then filtered through a fine glass fritted funnel under N_2_ pressure to remove insolubles. The filtrate was concentrated to 23 mg of a plastic-like film. This material was taken up in 0.75 mL of CDCl_3_, and 27 μL (0.77 mmol) of thiophenol was added via syringe. The reaction was followed by taking periodic ^1^H NMR spectra. Within 2 h, all of the material had been cleanly converted to bis(urazole) **6**. The solvent and excess thiophenol were removed by blowing over the solution with a stream of dry N_2_ gas to afford a white solid. The solid was partitioned between 10 mL of 0.5 M aq. NaOH and 10 mL of CH_2_Cl_2_. The aqueous layer was washed a second time with 10 mL of CH_2_Cl_2_, and the combined organic layers were concentrated to afford 8 mg of diphenyl disulfide, which was identified by TLC and ^1^H NMR analysis versus an authentic sample. The aqueous layer was acidified with conc. HCl to pH ∼ 2, and washed with 5 × 6 mL of CH_2_Cl_2_. The combined organic layers were dried over Na_2_SO_4_, filtered, and concentrated to afford 23 mg (100% yield) of bis(urazole) **6**, which was identified by TLC and ^1^H NMR analysis versus an authentic sample.

#### Bisurazole **8a**

To a solution of 1 g (8.2 mmol) of 3,5-dimethylphenol in 3 mL of ethanol was added 0.57 g (8.2 mmol) of solid sodium ethoxide. The mixture was warmed to 40 °C with a heating mantle and stirred for 0.5 h. To the resulting solution was added 0.26 mL (3.33 mmol) of 1,2-dichloroethane, and the resulting solution heated to 80 °C with a heating mantle for 24 h. The reaction mixture was cooled to room temperature, and a precipitate formed. The ethanol was removed via rotary evaporation, 25 mL of diethyl ether was added to the reaction mixture, and the contents were physically stirred thoroughly. The mixture was then filtered, and the separated solid rinsed well with ether. Concentration of the filtrate afforded 0.79 g of a brown solid which, from ^1^H NMR spectral analysis, consisted of a mixture of the diether, some monoether, and some starting phenol. The phenol was removed by taking the mixture up in 40 mL of CH_2_Cl_2_, washing it once with 40 mL of a 0.5 M aq. NaOH solution, drying it over Na_2_SO_4_, and reconcentrating it to a brown solid. Column chromatography (4:1 hexanes/EtOAc) afforded 0.322 g of a ∼2:1 mixture of the desired diether and the monoether, which coeluted on the column, as a white solid. This mixture was carried on for the next step.

To a stirring solution of the above mixture of compounds in 20 mL of CH_2_Cl_2_ was added 0.25 g (2.21 mmol) of MeTAD, followed by 170 μL (2.21 mmol) of CF_3_CO_2_H. The red color of the MeTAD decolorized within 5 h. The reaction mixture was concentrated, and the resulting solid subjected to column chromatography (5% CH_3_OH in EtOAc) to afford 0.44 g (41% yield based on MeTAD) of bis(urazole) **8a** as a pale gray solid, mp 255–256 °C: ^1^H NMR (400 MHz, DMSO-*d*_6_) δ 10.87 (s, 2H), 6.80 (s, 4H), 4.32 (s, 4H), 2.99 (s, 6H), 2.12 (s, 12H); ^13^C{^1^H} NMR (100 MHz, DMSO-*d*_6_) 158.7, 153.6, 151.4, 139.7, 125.8, 114.1, 66.4, 24.9, 17.6. HRMS (ESI) *m*/*z* [M + H]^+^ Calcd for C_24_H_29_N_6_O_6_ 497.2143, found 497.2140.

#### Oxidation of Bisurazole **8a**

To a mixture of 50 mg (0.10 mmol) of bis(urazole) **8a** in 2 mL of CHCl_3_ was added 100 mg (0.7 mmol) of Na_2_SO_4_ and 120 mg (0.44 mmol) of Ni_2_O_3_ (30% activity) with stirring. The reaction flask was fitted with a reflux condenser and drying tube and heated to a pot temperature of 80 °C using a heating mantle. After 3 h of reaction time, the mixture was cooled and filtered through a fine glass fritted funnel under N_2_ pressure to remove insolubles. The filtrate was concentrated to 43 mg of a white solid. Column chromatography (100% EtOAc) afforded 31 mg (63% yield) of dimer **4a**_**2**_ as a white solid: ^1^H NMR (400 MHz, CDCl_3_) δ 6.55 (d, *J* = 2.5 Hz, 4H), 6.20 (d, *J* = 2.5 Hz, 4H), 4.31(d, *J* = 7.8 Hz, 4H), 4.18 (d, *J* = 7.8 Hz, 4H), 3.27 (s, 12H), 2.31 (s, 12H), 1.71 (s, 12H); ^13^C{^1^H} NMR (100 MHz, CDCl_3_) 159.8, 153.9, 150.7, 141.8, 139.1, 123.6, 115.5, 112.6, 66.9, 26.2, 18.8, 17.4. HRMS (ESI) *m*/*z* [M + Cl]^−^ Calcd for C_48_H_52_N_12_O_12_Cl 1023.3522, found 1023.3539.

#### Reaction of Dimer **4a**_**2**_ with PhSH

To 31 mg (3.14 × 10^–5^ mol) of dimer **4a**_**2**_ in 1 mL of CDCl_3_ was added 45 μL (0.11 mmol, 3.5 equiv based on diradical content) of thiophenol. The resulting solution was transferred to an NMR tube, which was capped and sealed with parafilm. The reaction was followed by taking periodic ^1^H NMR spectra. Within 24 h, some crystals appeared on the walls of the NMR tube. After one week of reaction time, all of the signals corresponding to **4a**_**2**_ had vanished. The NMR tube was cut in half, the crystals were loosed from the sides of the tube with a thin spatula, and the mixture was filtered to afford 21 mg (68% yield) of crystalline bis(urazole) **8a**. The filtrate was concentrated by blowing over it with a stream of dry N_2_ gas. Analysis of the resulting solid by TLC and ^1^H NMR revealed the presence of diphenyl disulfide.

#### Bisurazole **8b**

To a solution of 1 g (8.2 mmol) of 3,5-dimethylphenol in 25 mL of DMF was added 0.91 g (8.2 mmol) of solid potassium *tert*-butoxide. The resulting solution was stirred for 0.5 to afford a clear, pale brown solution. To this solution was added 0.82 g (4.2 mmol) of 1,3-dibromopropane, and the reaction mixture stirred for 24 h. Salt precipitation began shortly after the addition of the dibromopropane and continued during the reaction time. The reaction mixture was poured into 50 mL of EtOAc, and the organic layer washed with 3 × 50 mL of H_2_O and 1 × 20 mL of sat. aq. NaCl. The organic layer was then dried over Na_2_SO_4_, filtered, and concentrated to a pale brown liquid. ^1^H NMR spectral analysis suggested the mixture consisted of the desired diether, the starting phenol, and other products. The phenol was removed by taking the mixture up in 20 mL of CH_2_Cl_2_, washing it once with 10 mL of a 0.5 M aq. NaOH solution, drying it over Na_2_SO_4_, and reconcentrating it to a brown solid. Column chromatography (9:1 hexanes/EtOAc) afforded 0.684 g of a nonresolvable mixture of the desired diether and likely the monoether. This mixture was carried on for the next step.

To a stirring solution of the above mixture of compounds in 30 mL of CH_2_Cl_2_ was added 0.45 g (3.98 mmol) of MeTAD followed by 300 μL (3.98 mmol) of CF_3_CO_2_H. The red color of the MeTAD decolorized within 1 h. The reaction mixture was concentrated, and the resulting solid subjected to column chromatography (2% CH_3_OH in EtOAc) to afford 0.54 g (27% yield based on MeTAD) of bis(urazole) **8b** as a white powder, mp 158–160 °C: ^1^H NMR (400 MHz, DMSO-*d*_6_) δ 10.86 (s, 2H), 6.77 (s, 4H), 4.13 (t, *J* = 6.2 Hz, 4H), 2.98 (s, 6H), 2.16 (p, *J* = 6.2 Hz, 2H), 2.11 (s, 12H); ^13^C{^1^H} NMR (100 MHz, DMSO-*d*_6_) 159.0, 153.7, 151.6, 139.9, 125.7, 114.2, 64.5, 28.6, 25.0, 17.7. HRMS (ESI) *m*/*z* [M + H]^+^ Calcd for C_25_H_31_N_6_O_6_ 511.2300, found 511.2303.

#### Oxidation of Bisurazole **8b**

To a mixture of 100 mg (0.20 mmol) of bis(urazole) **8b** in 4 mL of CH_2_Cl_2_ were added 200 mg (1.4 mmol) of Na_2_SO_4_ and 240 mg (0.8 mmol) of Ni_2_O_3_ (30% activity) with stirring. After 1 h of reaction time, the mixture was filtered through a fine glass fritted funnel under N_2_ pressure to remove insolubles. The filtrate was concentrated to 97 mg of a plastic-like film. Column chromatography (2:1 hexanes/EtOAc) afforded 64 mg (64% yield) of dimer **4b**_**2**_ as a white solid: ^1^H NMR (400 MHz, CDCl_3_) [present in solution as a mixture of two conformers in an approximate 5:1 ratio; signals are provided for the major conformer] δ 6.56 (d, *J* = 2.5 Hz, 4H), 6.16 (d, *J* = 2.5 Hz, 4H), 4.15 (t, *J* = 6.2 Hz, 8H), 3.26 (s, 12H), 2.31 (m, 4H), 2.29 (s, 12H), 1.61 (s, 12H); ^13^C{^1^H} NMR (100 MHz, CDCl_3_) [present in solution as a mixture of two conformers in an approximate 5:1 ratio; signals are provided for the major conformer with the corresponding minor signals in parentheses] 159.8, (154.1), 154.0, (150.4), 150.3, (142.1), 142.0, (139.5), 139.3, (123.3), 123.2, (114.6), 114.6, (114.4), 113.8, (65.0), 64.2, (29.4), 29.1, 26.2, 18.6, 17.5, (17.4). Heating this solution to 60 °C in the probe of the NMR instrument resulted in reversible coalescence of minor signals in both the ^1^H and ^13^C NMR spectra (see the Supporting Information). HRMS (ESI) *m*/*z* [M + Cl]^−^ Calcd for C_50_H_56_N_12_O_12_Cl 1051.3835, found 1051.3824.

#### Reaction of Dimer **4b**_**2**_ with PhSH

To 57 mg (0.1 mmol) of dimer **4b**_**2**_ in 2 mL of CDCl_3_ was added 30 μL (0.25 mmol, 2.5 equiv based on diradical content) of thiophenol. The resulting solution was transferred to an NMR tube, which was capped and sealed with parafilm. The reaction was followed by taking periodic ^1^H NMR spectra. After four days of reaction time, all of the signals corresponding to dimer **4b**_**2**_ had vanished. The reaction mixture was concentrated by blowing over it with a stream of dry N_2_ gas. The resulting residue was taken up in 10 mL of CH_2_Cl_2_, and the organic layer washed with 1 × 10 mL of 0.5 N aq. NaOH. The organic layer was removed, and the aqueous layer was backwashed with 1 × 5 mL of CH_2_Cl_2_. These combined organic layers were dried over Na_2_SO_4_, filtered, and concentrated to afford 21 mg of a white solid (86% of expected yield). ^1^H NMR spectroscopic and TLC analysis identified this solid as diphenyl disulfide. The aqueous layer was acidified to pH ∼ 2 and washed with 3 × 5 mL of CH_2_Cl_2_. The combined organic layers were dried over Na_2_SO_4_, filtered, and concentrated to afford 47 mg (82% yield) of bis(urazole) **8b** as identified by ^1^H NMR and TLC analysis versus authentic material.

#### Bisurazole **8c**

To a solution of 1 g (8.2 mmol) of 3,5-dimethylphenol in 25 mL of dry DMF was added 0.91 g (8.2 mmol) of solid potassium *tert*-butoxide. The resulting solution was stirred for 0.5 to afford a clear, pale brown solution. To this solution was added 0.94 g (4.2 mmol) of 1,3-dibromopentane, and the reaction mixture stirred for 24 h. Salt precipitation began shortly after the addition of the dibromopentane and continued during the reaction time. The reaction mixture was poured into 50 mL of EtOAc, and the organic layer was washed with 3 × 50 mL of 0.5 M aq. NaOH and 1 × 20 mL sat. aq. NaCl. The organic layer was then dried over Na_2_SO_4_, filtered, and concentrated to a thick liquid that crystallized upon standing. The solid was taken up in 10 mL of acetone, and methanol was added until the solution became slightly cloudy (∼35 mL). Cooling in a freezer afforded white needles that were isolated via filtration and rinsed with cold methanol to afford 0.91 g (71%yield) of diether **7c**, mp 51–52 °C (lit.^13^ 48–49 °C): ^1^H NMR (400 MHz, CDCl_3_) δ 6.58 (s, 2H), 6.53 (s, 4H), 3.95 (t, *J* = 6.5 Hz, 4H), 2.28 (s, 12H), 1.83 (p, *J* = 6.3 Hz, 4H), 1.63 (m, 2H); ^13^C{^1^H} NMR (100 MHz, CDCl_3_)δ 159.2, 139.3, 122.5, 112.4, 67.7, 29.2, 22.9, 21.6. HRMS (ESI) *m*/*z* [M + H]^+^ Calcd for C_21_H_29_O_2_ 313.2162, found 313.2164.

To a stirring solution of 0.5 g (1.60 mmol) of diether **7c** in 15 mL of CH_2_Cl_2_ was added 380 mg (3.36 mmol) of MeTAD as a solid. To the resulting red solution was added 125 μL (1.6 mmol) of TFA via syringe. After stirring 24 h, the pale pink solution was condensed in vacuo and subjected to column chromatography (95:5 CH_2_Cl_2_ /CH_3_OH) to afford 0.82 g (95% yield) of bis(urazole) **8c** as a white foam: ^1^H NMR (400 MHz, DMSO-*d*_6_) δ 10.85 (s, 2H), 6.75 (s, 4H), 4.00 (t, *J* = 6.4 Hz, 4H), 2.98 (s, 6H), 2.11 (s, 12H), 1.77 (p, *J* = 6.4 Hz, 4H), 1.55 (m, 2H); ^13^C{^1^H} NMR (100 MHz, CDCl_3_)δ 159.8, 154.7, 151.6, 139.8, 124.5, 114.3, 67.8, 28.9, 25.4, 22.8, 18.0. HRMS (ESI) *m*/*z* [M + H]^+^ Calcd for C_27_H_35_N_6_O_6_ 539.2613, found 539.2618.

#### Oxidation of Bisurazole **8c**

To a solution of 200 mg (0.37 mmol) of bis(urazole) **8c** in 5 mL of CH_2_Cl_2_ were added 400 mg (2.8 mmol) of Na_2_SO_4_ and 440 mg (1.48 mmol) of Ni_2_O_3_ (30% activity) with stirring. After 1 h of reaction time, the mixture was filtered through a fine glass fritted funnel under N_2_ pressure to remove insolubles. The filtrate was concentrated to 184 mg of a plastic-like film. Column chromatography (1:1 hexanes/EtOAc) afforded 35 mg (18% yield) of monomer **4c**_**1**_ as a white solid: ^1^H NMR (400 MHz, CDCl_3_) δ 6.45 (d, *J* = 2.6 Hz, 2H), 6.18 (d, *J* = 2.6 Hz, 2H), 4.11 (m, 2H), 3.99 (m, 2H), 3.26 (s, 6H), 2.27 (s, 6H), 1.90 (m, 2H), 1.78 (s, 6H), 1.74 (m, 2H), 1.63 (m, 2H); ^13^C{^1^H} NMR (100 MHz, CDCl_3_) δ159.6, 154.5, 151.4, 141.5, 139.0, 123.8, 115.2, 114.0, 68.3, 27.7, 26.2, 23.9, 18.6, 17.6. HRMS (ESI) *m*/*z* [M + H]^+^ Calcd for C_27_H_33_N_6_O_6_ 537.2456, found 537.2461.

Also isolated was 149 mg (75% yield) of dimer **4c**_**2**_ as a white solid: ^1^H NMR (400 MHz, CDCl_3_) [present in solution as a mixture of two conformers in an approximate 3:1 ratio; signals are provided for the major conformer] δ 6.53 (d, *J* = 2.5 Hz, 4H), 6.08 (d, *J* = 2.5 Hz, 4H), 3.94 (t, *J* = 6.4 Hz, 8H), 3.25 (s, 12H), 2.27 (s, 12H), 1.92 (m, 8H), 1.74 (m, 4H), 1.60 (s, 12H); ^13^C{^1^H} NMR (100 MHz, CDCl_3_) [present in solution as a mixture of two conformers in an approximate 3:1 ratio; signals are provided for the major conformer with the corresponding minor signals in parentheses] δ 160.0, 154.0 (154.1), 150.3 (150.2), 141.8 (141.9), 139.3 (139.4), 122.8 (122.7), 114.3 (114.4), 113.8 (113.9), 67.9 (67.8), 29.4 (29.3), 26.1, 23.1 (22.6), 18.6, 17.4 (17.3). HRMS (ESI) *m*/*z* [M + H]^+^ Calcd for C_54_H_65_N_12_O_12_ 1073.4839, found 1073.4835.

#### Dependence of the Ratio of Dimer **4c**_**2**_ to Monomer **4c**_**1**_ under Various Conditions

##### Solvent Dependence

A 10 mg mixture of **4c**_**1**_ and **4c**_**2**_ was dissolved in 0.75 mL of CDCl_3_, then allowed to stand for 24 h at room temperature, and a ratio of the two compounds was determined by ^1^H NMR spectroscopic analysis by making use of integrations of relevant signals. The CDCl_3_ was removed in vacuo, and the sample taken up in 0.75 mL of C_6_D_6_. The sample was allowed to stand for 24 h at room temperature, and the ratio determined. This process was repeated with 0.75 mL of DMSO-*d*_6_.

##### Concentration Dependence

A stock solution of 0.134 g of freshly oxidized bis(urazole) **8c** in 2 mL of CDCl_3_ was diluted with additional CDCl_3_ into individual NMR tubes to afford the concentrations listed in the text. Each tube was sealed with a cap and parafilm and allowed to stand at room temperature for 24 h prior to analysis. A ratio of the two compounds was determined by ^1^H NMR spectroscopic analysis by making use of integrations of relevant signals. Spectra are provided in the Supporting Information.

##### Temperature Dependence

A solution of 18 mg of a mixture of **4c**_**1**_ and **4c**_**2**_ in 0.75 mL DMSO-*d*_6_ in an NMR tube was frozen, the tube was evacuated, and then the tube flame-sealed. After allowing the sample to equilibrate at 20 °C for 24 h, a ratio of the two compounds was determined by ^1^H NMR spectroscopic analysis by making use of integrations of relevant signals. The sample was placed in a preheated oil bath at 30 °C and allowed to equilibrate at that temperature for 24 h prior to analysis. This process was repeated for temperatures of 40, 50, and 60 °C. Upon heating at 70 °C, evidence for decomposition, with the formation of bis(urazole) **8c**, was observed. Spectra are provided in the Supporting Information.

#### Reaction of Dimer **4c**_**2**_ with PhSH

To 30 mg (0.06 mmol) of dimer **4c**_**2**_ in 0.75 mL of CDCl_3_ was added 14 μL (0.15 mmol, 2.5 equiv based on diradical content) of thiophenol. The resulting solution was transferred to an NMR tube, which was capped and sealed with parafilm. The reaction was followed by taking periodic ^1^H NMR spectra. After four days of reaction time, all the signals corresponding to **4c**_**2**_ had vanished. The reaction mixture was concentrated by blowing over it with a stream of dry N_2_ gas. The resulting residue was taken up in 10 mL of CH_2_Cl_2_, and the organic layer washed with 1 × 10 mL of 0.5 N aq, NaOH. The organic layer was removed, and the aqueous layer washed with 1 × 5 mL of CH_2_Cl_2_. These combined organic layers were dried over Na_2_SO_4_, filtered, and concentrated to afford 10 mg of a white solid (83% of expected yield). ^1^H NMR spectroscopic and TLC analysis identified this solid as diphenyl disulfide. The aqueous layer was acidified to pH ∼ 2 and washed 3 × 5 mL of CH_2_Cl_2_. The combined organic layers were dried over Na_2_SO_4_, filtered, and concentrated to afford 25 mg (83% yield) of bis(urazole) **8c** as identified by ^1^H NMR and TLC analysis versus authentic material.

#### Reaction of Monomer **4c**_**1**_ with PhSH

To 25 mg (0.1 mmol) of monomer **9** in 0.75 mL of CDCl_3_ was added 12 μL (0.25 mmol, 2.5 equiv based on diradical content) of thiophenol. The resulting solution was transferred to an NMR tube, which was capped and sealed with parafilm. The reaction was followed by taking periodic ^1^H NMR spectra. After just 7 h of reaction time, all the signals corresponding to **4c**_**1**_ had vanished. ^1^H NMR spectroscopic and TLC analysis indicated only the presence of bis(urazole) **8c**, diphenyl disulfide, and residual thiophenol.

#### Bisurazole **8d**

To a solution of 1 g (8.2 mmol) of 3,5-dimethylphenol in 25 mL of dry DMF was added 0.91 g (8.2 mmol) of solid potassium *tert*-butoxide. The resulting solution was stirred for 0.5 h to afford a clear, pale brown solution. To this solution was added 0.85 g (3.3 mmol) of 1,3-dibromoheptane, and the reaction mixture stirred for 24 h. Salt precipitation began shortly after the addition of the dibromoheptane and continued during the reaction time. The reaction mixture was poured into 50 mL of EtOAc, and the organic layer washed with 3 × 50 mL of 0.5 M aq. NaOH and 1 × 20 mL sat. aq. NaCl. The organic layer was then dried over Na_2_SO_4_, filtered, and concentrated to a milky liquid. Column chromatography (4:1 hexanes: EtOAc) afforded 0.81 g (73% yield) of diether **7d** as a clear liquid that solidified into a waxy solid upon standing: ^1^H NMR (400 MHz, CDCl_3_) δ 6.58 (s, 2H), 6.52 (s, 4H), 3.91 (t, *J* = 6.5 Hz, 4H), 2.28 (s, 12H), 1.56 (p, *J* = 6.3 Hz, 4H), 1.44 (m, 6H); ^13^C{^1^H} NMR (100 MHz, CDCl_3_)δ 159.2, 139.1, 122.3, 112.3, 67.7, 29.4, 29.2, 26.1, 21.5. HRMS (ESI) *m*/*z* [M + H]^+^ Calcd for C_23_H_33_O_2_ 341.2475, found 341.2473.

To a stirring solution of 0.32 g (0.94 mmol) of diether **7d** in 10 mL of CH_2_Cl_2_ was added 218 mg (1.97 mmol) of MeTAD as a solid. To the resulting red solution was added 150 μL (1.97 mmol) of TFA via syringe. After stirring for 3 h, the pale pink solution was condensed in vacuo to a viscous liquid and subjected to column chromatography (95:5 CH_2_Cl_2_/CH_3_OH) to afford 0.52 g (98% yield) of bis(urazole) **8d** as a white solid, mp 67–68 °C: ^1^H NMR (400 MHz, CDCl_3_) δ 8.19 (v. br s, 2H), 6.59 (s, 4H), 3.93 (t, *J* = 6.9 Hz, 4H), 3.08 (s, 6H), 2.13 (s, 12H), 1.79 (p, *J* = 6.9 Hz, 4H), 1.50 (m, 6H); ^13^C{^1^H} NMR (100 MHz, CDCl_3_)δ 160.1, 154.7, 151.6, 139.8, 124.2, 114.4, 68.0, 29.1, 29.0, 26.0, 25.5, 18.0. HRMS (ESI) *m*/*z* [M + H]^+^ Calcd for C_29_H_39_N_6_O_6_ 567.2926, found 567.2931.

#### Oxidation of Bisurazole **8d**

To a solution of 50 mg (0.09 mmol) of bis(urazole) **8d** in 1 mL of CH_2_Cl_2_ were added 100 mg (0.7 mmol) of Na_2_SO_4_ and 110 mg (0.36 mmol) of Ni_2_O_3_ (30% activity) with stirring. After 1 h of reaction time, the mixture was filtered through a fine glass fritted funnel under N_2_ pressure to remove insolubles. The filtrate was concentrated to 47 mg of a plastic-like film. Column chromatography (1:1 hexanes/EtOAc) afforded 11 mg (22% yield) of monomer **4d**_**1**_ as a plastic-like clear colorless film: ^1^H NMR (400 MHz, CDCl_3_) δ 6.48 (d, *J* = 2.5 Hz, 2H), 6.15 (d, *J* = 2.5 Hz, 2H), 3.98 (ddd, *J* = 9.2, 6.7, 4.9 Hz, 2H), 3.80 (dt, *J* = 8.7, 6.7 Hz, 2H), 3.25 (s, 6H), 2.27 (s, 6H), 1.94–1.73 (m, [4H]), 1.70 (s, 6H), 1.72–1.60 (m, [2H]), 1.57–1.43 (m, 4H) (note: proton counts in [H] are the predicted number of hydrogens, the actual number being affected by the presence of small amounts of dimer in the sample); ^13^C{^1^H} NMR (100 MHz, CDCl_3_) δ160.2, 154.3, 151.0, 141.4, 139.0, 123.5, 115.1, 113.6, 67.2, 27.1, 26.2, 25.0, 23.9, 18.7, 17.4; HRMS (ESI) *m*/*z* [M + H]^+^ Calcd for C_29_H_37_N_6_O_6_ 565.2769, found 565.2774.

Also isolated was 28 mg (56% yield) of dimer **4d**_**2**_ as a white solid: ^1^H NMR (400 MHz, CDCl_3_) [present in solution as a mixture of two conformers in an approximate 1.5:1 ratio, the signals of which were overlapping with the exception of one of the aromatic protons] δ 6.52 (m, 4H), 6.07 (d, *J* = 2.9 Hz, 4H) [signal for the minor conformer, 6.10 (d, *J* = 2.9 Hz 4H)], 3.90 (t, *J* = 6.4 Hz, 8H), 3.25 (s, 12H), 2.26 (s, 12H), 1.82 (m, 8H), 1.59 (s, 12H), 1.56 (m, 12H); ^13^C{^1^H} NMR (100 MHz, CDCl_3_) [present in solution as a mixture of two conformers in an approximate 1.5:1 ratio; signals are provided for the major conformer with the corresponding minor signals in parentheses] δ 160.1, 154.0 (154.1), 150.3 (150.2), 141.8 (141.9), 139.3 (139.4), 122.7 (122.6), 114.4 (114.5), 113.8 (113.9), 68.0 (68.1), 29.6 (29.7), 26.2 (24.8), 26.1 (26.4), 18.6, 17.3. HRMS (ESI) *m*/*z* [M + NO_3_]^−^ Calcd for C_58_H_72_N_13_O_15_ 1190.5276, found 1190.5248.

#### Reaction of a Mixture of Monomer **4d**_**1**_ and Dimer **4d**_**2**_ with PhSH

To 22 mg of a mixture of monomer **4d**_**1**_ and dimer **4d**_**2**_ in 0.75 mL of CDCl_3_ was added 10 μL (0.13 mmol, 2.5 equiv based on diradical content) of thiophenol. The resulting solution was transferred to an NMR tube, which was then capped and sealed with parafilm. The reaction was followed by taking periodic ^1^H NMR spectra. Over a 24 h period, all the signals corresponding to compounds **4d**_**1**_ and **4d**_**2**_ had vanished. ^1^H NMR spectroscopic and TLC analysis indicated only the presence of bis(urazole) **8d**, diphenyl disulfide, and residual thiophenol.

#### Bisurazole **8e**

To a solution of 1 g (8.2 mmol) of 3,5-dimethylphenol in 25 mL of dry DMF was added 0.91 g (8.2 mmol) of solid potassium *tert*-butoxide. The resulting solution was stirred for 0.5 to afford a clear, pale brown solution. To this solution was added 1.0 g (3.3 mmol) of 1,1-dibromoundecane, and the reaction mixture stirred for 24 h. Salt precipitation began shortly after the addition of the dibromoundecane and continued during the reaction time. The reaction mixture was poured into 50 mL of EtOAc, and the organic layer washed with 3 × 50 mL of 0.5 M aq. NaOH and 1 × 20 mL sat. aq. NaCl. The organic layer was then dried over Na_2_SO_4_, filtered, and concentrated to a thick colorless liquid that crystallized upon standing. The compound was dissolved in 10 mL of acetone to which 50 mL of CH_3_OH was slowly added with swirling. Colorless crystals precipitated, which were isolated via filtration to afford 0.71 g of **7e**. Chilling the mother liquor in the freezer for several hours provided an additional 0.40 g of **7e** (86% yield total), mp 44–45 °C: ^1^H NMR (400 MHz, CDCl_3_) δ 6.57 (s, 2H), 6.52 (s, 4H), 3.91 (t, *J* = 6.7 Hz, 4H), 2.27 (s, 12H), 1.75 (p, *J* = 6.7 Hz, 4H), 1.42 (br p, *J* = 6.7 Hz, 4H), 1.30 (br m, 10H); ^13^C{^1^H} NMR (100 MHz, CDCl_3_) δ 159.3, 139.2, 122.3, 112.4, 67.8, 29.70, 29.66, 29.5, 29.4, 26.2, 21.6. HRMS (ESI) *m*/*z* [M + H]^+^ Calcd for C_27_H_41_O_2_ 397.3101, found 397.3097.

To a stirring solution of 0.57 g (1.43 mmol) of bisether **7e** in 15 mL of CH_2_Cl_2_ was added 340 mg (3 mmol) of MeTAD as a solid. To the resulting red solution was added 230 μL (3 mmol) of TFA via syringe. After stirring for 1 h, the pale pink solution was condensed in vacuo to a viscous liquid and subjected to column chromatography (95:5 CH_2_Cl_2_ /CH_3_OH) to afford 0.85 g (96% yield) of bis(urazole) **8e** as a white foam that turned into a glass upon standing: ^1^H NMR (400 MHz, CDCl_3_) δ 7.83 (v. br s, 2H), 6.60 (s, 4H), 3.92 (t, *J* = 6.7 Hz, 4H), 3.12 (s, 6H), 2.15 (s, 12H), 1.77 (p, *J* = 6.7 Hz, 4H), 1.50–1.25 (m, 14H); ^13^C{^1^H} NMR (100 MHz, CDCl_3_) δ 160.2, 154.7, 151.5, 139.8, 124.0, 114.5, 68.2, 29.6, 29.5, 29.4, 29.2, 26.0, 25.6, 18.0. HRMS (ESI) *m*/*z* [M + H]^+^ Calcd for C_33_H_47_N_6_O_6_ 623.3552, found 623.3549.

#### Oxidation of Bisurazole **8e**

To a solution of 50 mg (0.09 mmol) of bis(urazole) **8e** in 3 mL of CH_2_Cl_2_ were added 100 mg (0.7 mmol) of Na_2_SO_4_ and 100 mg (0.3.6 mmol) of Ni_2_O_3_ (30% activity) with stirring. After 1 h of reaction time, the mixture was filtered through a fine glass fritted funnel under N_2_ pressure to remove insolubles. The filtrate was concentrated to 48 mg of a plastic-like film. Column chromatography (1:1 hexanes/EtOAc) afforded 7 mg (22% yield) of monomer **4e**_**1**_ as a plastic-like clear colorless film: ^1^H NMR (400 MHz, CDCl_3_) δ 6.51 (d, *J* = 2.7 Hz, 2H), 6.12 (d, *J* = 2.7 Hz, 2H), 3.89 (t, *J* = 5.9 Hz, 4H), 3.24 (s, 6H), 2.26 (s, 6H), 1.77 (m, 4H), 1.78 (s, 6H), 1.55 (s, 6H), 1.65–1.34 (m, 14H); ^13^C{^1^H} NMR (100 MHz, CDCl_3_) δ 160.5, 154.2, 150.1, 141.9, 139.2, 122.4, 116.0, 112.6, 68.5, 28.5, 27.2, 26.7, 26.1, 25.6, 24.6, 18.6, 17.1. HRMS (ESI) *m*/*z* [M + H]^+^ Calcd for C_33_H_44_N_6_O_6_ 621.3395, found 621.3400.

Also isolated was 22 mg (44% yield) of dimer **4e**_**2**_ as a white solid: ^1^H NMR (400 MHz, CDCl_3_) [present in solution as a mixture of two conformers in an approximate 1:1 ratio, the signals of which were coincidental in the ^1^H NMR spectrum] δ 6.51 (d, *J* = 2.5 Hz, 4H), 6.08 (br s, 4H), 3.87 (br t, *J* = 6.5 Hz, 8H), 3.25 (s, 12H), 2.26 (s, 12H), 1.80 (p, *J* = 2.5 Hz, 8H), 1.58 (s, 12H), 1.54–1.30 (m, 28H); ^13^C{^1^H} NMR (100 MHz, CDCl_3_) [present in solution as a mixture of two conformers in an approximate 1:1 ratio; the second set of signals was generally very close to the first, in most cases only being differentiated if line broadening was not applied, there fore the chemical shift values are provided as if for a single conformer, but a doubled signal is represented as (2)] δ 160.2, 154.1, 150.2(2), 141.9(2), 139.4(2), 122.6(2), 114.5(2), 113.8(2), 68.2, 30.0(2), 29.9(2), 29.8(2), 29.6, 26.4(2), 26.1, 18.5, 17.2. HRMS (ESI) *m*/*z* [M + H]^+^ Calcd for C_66_H_89_N_12_O_12_ 1241.6717, found 1241.6754.

#### Reaction of a Mixture of Monomer **4e**_**1**_ and Dimer **4e**_**2**_ with PhSH

To 25 mg of a mixture of monomer **4e**_**1**_ and dimer **4e**_**2**_ in 0.75 mL of CDCl_3_ was added 10 μL (0.13 mmol, 2.5 equiv based on diradical content) of thiophenol. The resulting solution was transferred to an NMR tube, which was then capped and sealed with parafilm. The reaction was followed by taking periodic ^1^H NMR spectra. Over a 24 h period, all the signals corresponding to **4e**_**1**_ and **4e**_**2**_ had vanished. ^1^H NMR spectroscopic and TLC analysis indicated only the presence of bis(urazole) **8e**, diphenyl disulfide, and residual thiophenol. The reaction mixture was concentrated by blowing over it with a stream of dry N_2_ gas, and the resulting residue diluted with 5 mL CH_2_Cl_2_. This solution was washed with 0.5 M aq. NaOH. The aqueous layer was backwashed with 1 × 2 mL CH_2_Cl_2_ and then acidified with conc. HCl to pH ∼ 2. This solution was washed with 3 × 3 mL of CH_2_Cl_2_. The combined organic layers were dried over Na_2_SO_4_, filtered, and concentrated to afford 19 mg (76% yield) of bis(urazole) **8e** as a white solid.

### Computational Details

All calculations were carried out at the ωB97X-D/6-31G* level of theory using the Gaussian 16 suite of software.^14^ Frequency calculations were carried out at the same level of theory to ensure that the optimized geometry represented a true minimum (i.e., no imaginary frequencies), and to provide zero-point energies used for Gibbs free energy, enthalpy, and entropy values.

### X-ray Crystallographic Analysis

The diffraction data were collected on a Rigaku XtaLAB Synergy-S Dualflex HyPix diffractometer with monochromated Cu–K_α_ radiation. The structure was solved by direct methods (OLEX2.solve)^15,16^ and refined by full-matrix least-squares on F^2^ values (SHELXL).^17^ All the heavy atoms were refined anisotropically. The hydrogen atoms were localized from the difference electron density maps, after which they were refined isotropically (*U*_iso_ with a factor of 1.2 for CH and CH_2_ groups and that of 1.5 for CH_3_ groups) with riding coordinates or as rotation CH_3_ groups. Mercury was used for the structure presentation in the figures.^18^

#### Compound **4a**_**2**_

Colorless single crystals were obtained for dimer **4a**_**2**_ by slow diffusion of a layer of methanol (in which **4a**_**2**_ is insoluble) into a solution of the dimer in CH_2_Cl_2_. A colorless block-like crystal (0.33 mm × 0.13 mm × 0.10 mm) was used for diffraction measurements at 100 K. Monoclinic, space group *P*2_1_, *a* = 8.83819(7) Å, *b* = 14.23369(11) Å, *c* = 20.10392(16) Å, β = 91.7994(7)°, *V* = 2527.83(3) Å^3^, *Z* = 2, *F*(000) = 1124, ρ_calc_ = 1.411 Mg/m^3^, *R*_1_[*I* > 2σ(*I*)] = 0.0338, w*R*_2_[all data] = 0.0896, and GOF = 1.035. CCDC no. 2253254.

#### Compound **4b**_**2**_

Colorless single crystals were obtained for dimer **4b**_**2**_ by slow diffusion of a layer of methanol (in which **4b**_**2**_ is insoluble) into a solution of the dimer in CH_2_Cl_2_. A colorless block-like crystal (0.37 mm × 0.30 mm × 0.21 mm) was used for diffraction measurements at 109 K. Monoclinic, space group *I*2/*a*, *a* = 27.2958(4) Å, *b* = 17.6590(5) Å, *c* = 27.3191(6) Å, β = 95.6702(16)°, *V* = 13103.8(5) Å^3^, *Z* = 8, *F*(000) = 5680, ρ_calc_ = 1.394 Mg/m^3^, *R*_1_[*I* > 2σ(*I*)] = 0.0707, w*R*_2_[all data] = 0.2169, GOF = 1.060. CCDC no. 2253255.

#### Compound **4c**_**2**_

Colorless single crystals of **4c**_**2**_ were obtained by slow evaporation of a solution of the dimer in dimethylcarbonate. A colorless plate-like crystal (0.27 mm × 0.21 mm × 0.10 mm) was used for diffraction measurements at 100 K. Monoclinic, space group *P*2_1_/*c*, *a* = 8.92534(8) Å, *b* = 14.21670(12) Å, *c* = 51.8332(4) Å, β = 90.4433(8)°, *V* = 6576.87(9) Å^3^, *Z* = 4, *F*(000) = 2848, ρ_calc_ = 1.357 Mg/m^3^, *R*_1_[*I* > 2σ(*I*)] = 0.0448, w*R*_2_[all data] = 0.1150, GOF = 1.020. CCDC no. 2253256.

## Data Availability

The data underlying this study are available in the published article and its Supporting Information.

## Supporting Information Available

^1^H and ^13^C NMR spectra for all newly reported structures; DOSY spectra for mixtures of compounds **4c**_**1**_/**4c**_**2**_ and **4e**_**1**_/**4e**_**2**_; computational data for compounds **4c**_**1**_/**4c**_**2**_; Ortep diagrams and crystallographic data for compounds **4a**_**2**_, **4b**_**2**_, and **4c**_**2**_ (PDF)
