# Supplementary material for: Evaluating the Effectiveness of Tethered Bis(urazolyl) Diradicals as Molecular Building Blocks for Dynamic Covalent Chemistry
Source: J Org Chem. 2023 Jun 26;88(13):9157–66. doi: 10.1021/acs.joc.3c00732 (PMC10337031; doi:10.1021/acs.joc.3c00732)
Supplement: Supplementary file 1 — jo3c00732_si_001.pdf [file jo3c00732_si_001.pdf]

Supporting Information

Evaluating the Effectiveness of Tethered Bis-Urazolyl Diradicals as Molecular Building Blocks for Dynamic Covalent Chemistry

Gary W. Breton[1]*, Kenneth L. Martin[1], James Alexander Bowron, Jr. [1], and John Bacsa[2]

[1]*Department of Chemistry, Berry College, Mount Berry, GA, 30149 USA*

[2] *Department of Chemistry, Emory University, Atlanta, GA, 30322 USA*

*Corresponding Author: gbreton@berry.edu

Table of Contents

^{1}H and ^{13}C NMR Spectra of Relevant Compounds……………………………………….S2–S20

DOSY Spectra……………………………………………………………………………………………S21–S22

^{1}H NMR Spectra Used to Determine the Temperature Dependence of the Equilibrium Between Monomer **4c₁** and Dimer **4c₂**……………………………………………………………………. S23–S25

^{1}H NMR Spectra Used to Determine the Concentration Dependence of the Equilibrium Between Monomer **4c₁** and Dimer **4c₂**……………………………………………………………………. S26–S29

Computational Details for Optimized Structures………………………………………S30–S35

Crystal Structure Data……………………………………………………………………………...S36–S86

^{1}H NMR Spectrum of Compound 6 (DMSO-d_6, 400 MHz)

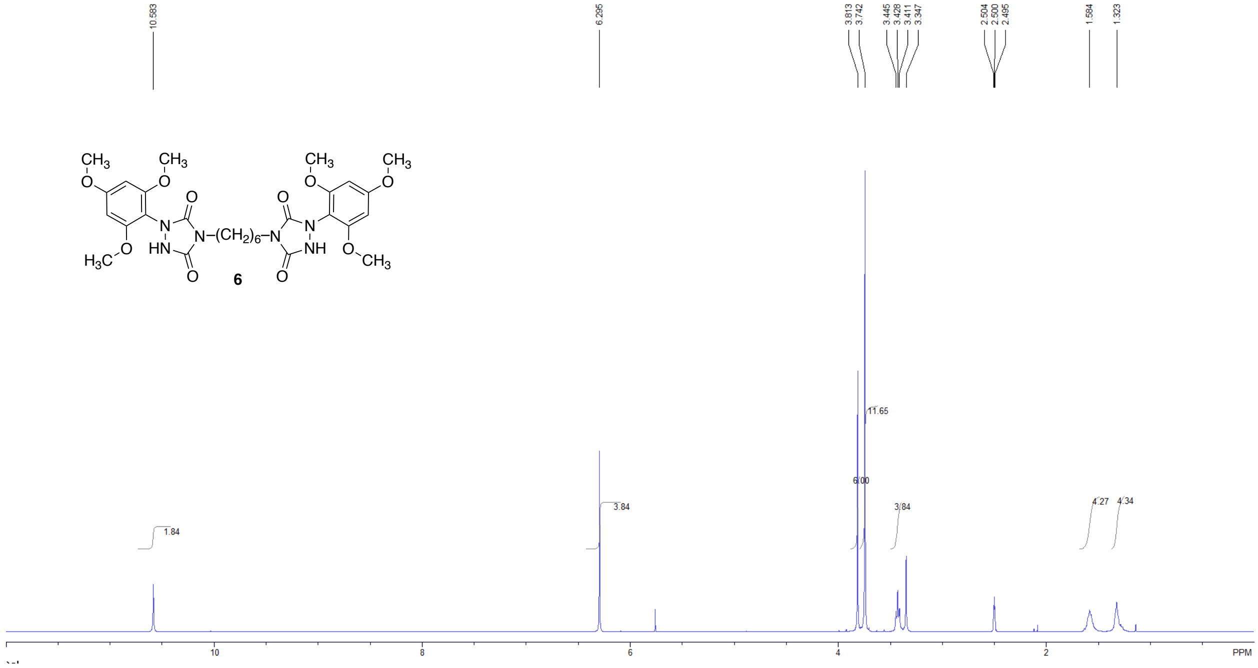

^{13}C{^{1}H} NMR Spectrum of Compound 6 (DMSO-d_6, 100 MHz)

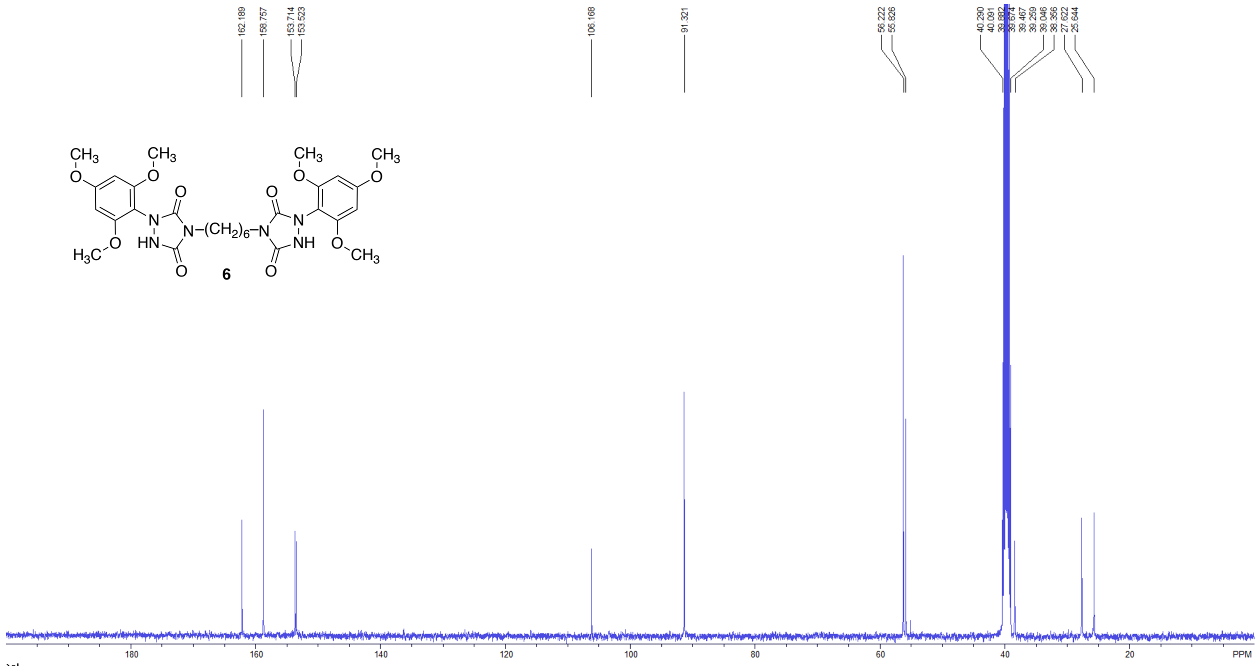

^{1}H NMR Spectrum of Oligomeric Product Resulting from Oxidation of Bisurazole 6 ($CDCl_3$, 400 MHz)

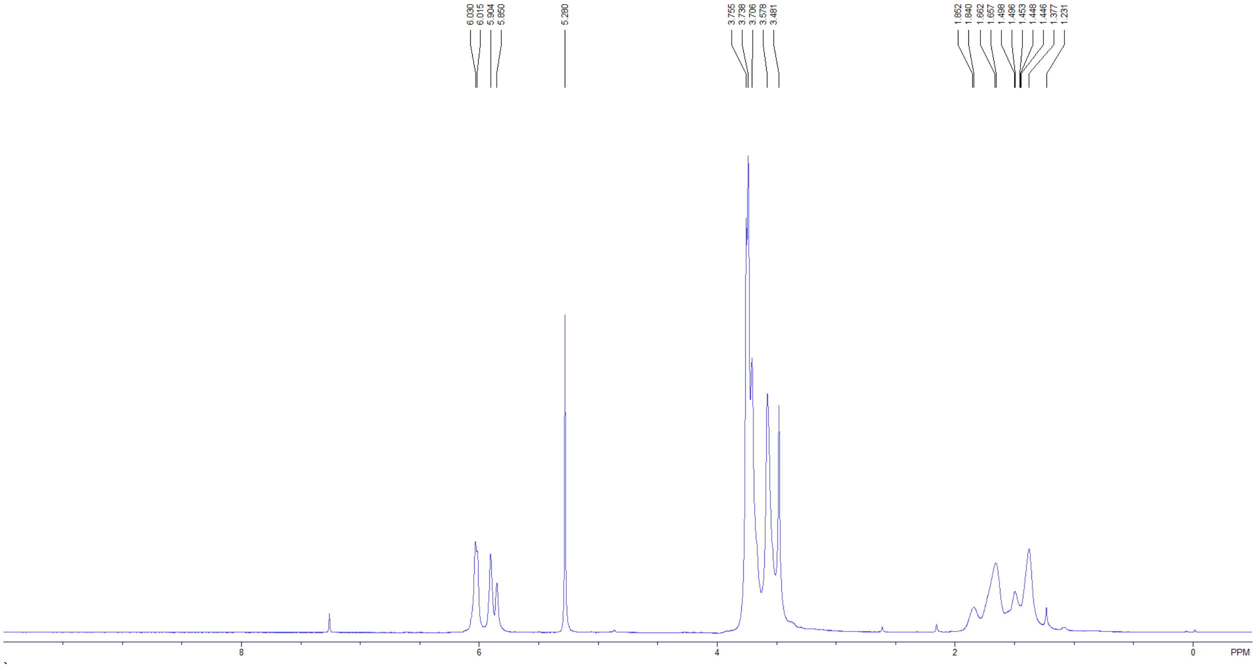

^{1}H NMR Spectrum of Bisurazole 8a (DMSO-d$_6$, 400 MHz)

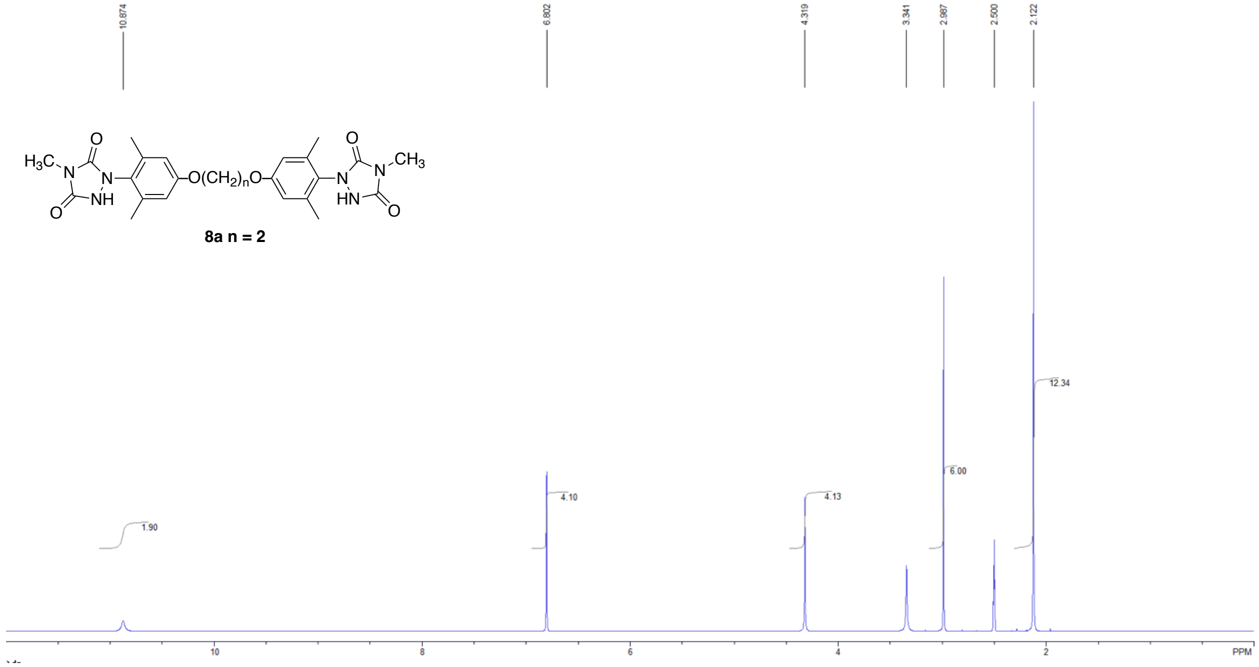

^{13}C{^{1}H} NMR Spectrum of Bisurazole 8a (DMSO-d$_6$, 100 MHz)

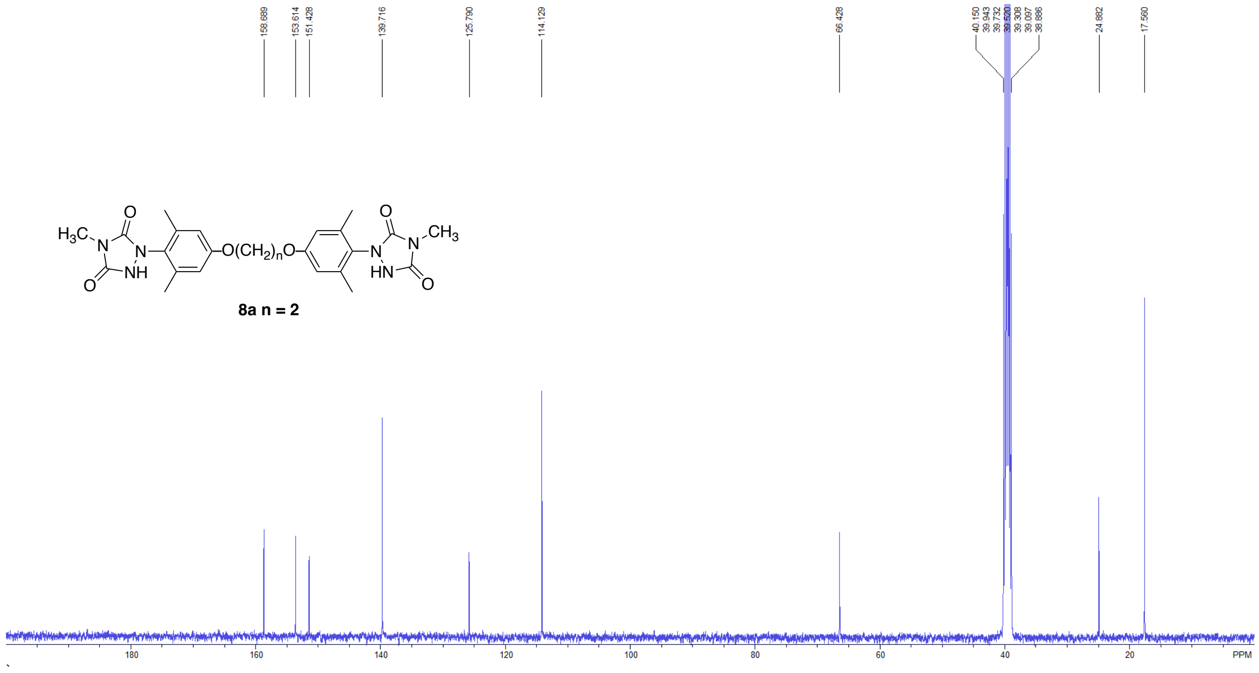

^{1}H NMR Spectrum of Dimer $4a_2$ ($CDCl_3$, 400 MHz)

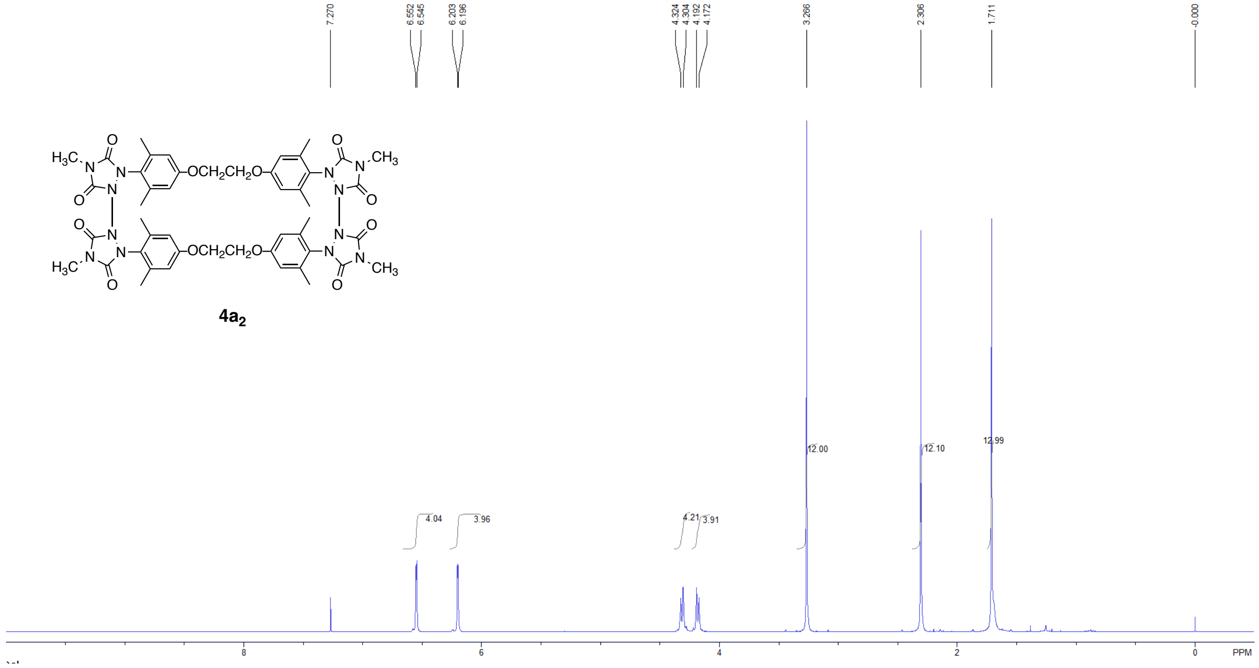

$^{13}C\{^{1}H\}$ NMR Spectrum of Dimer $4a_2$ ($CDCl_3$, 100 MHz)

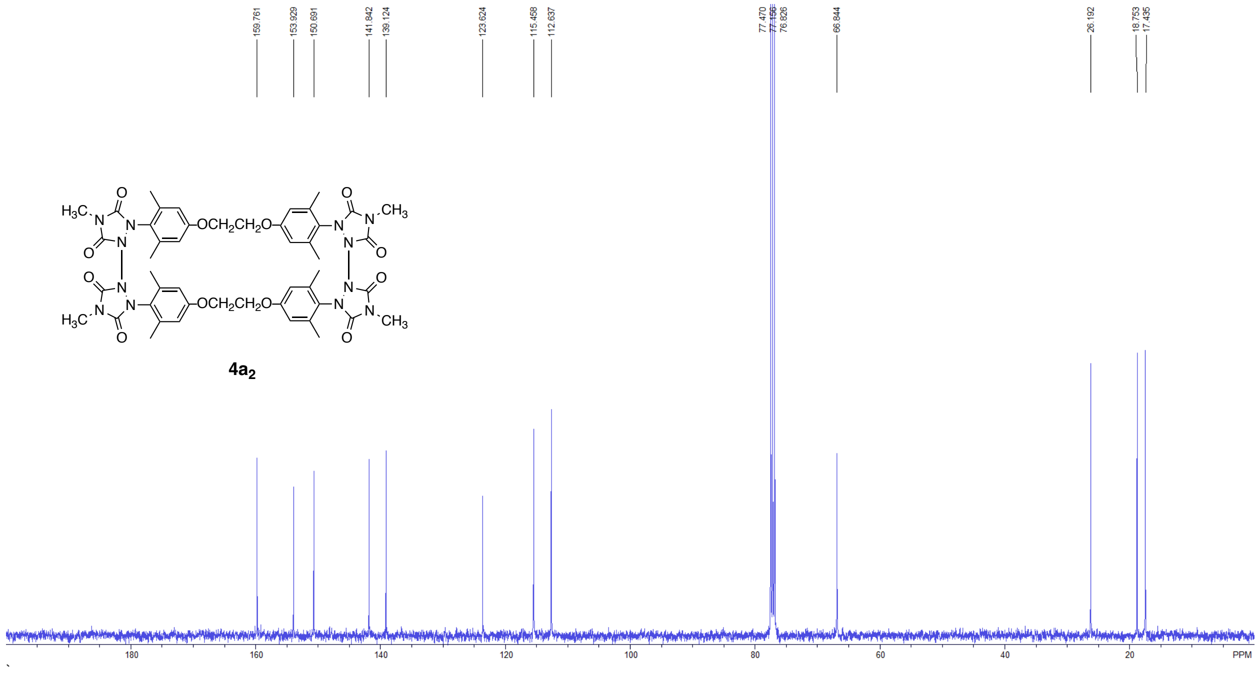

^{1}H NMR Spectrum of Bisurazole 8b (DMSO-d$_6$, 400 MHz)

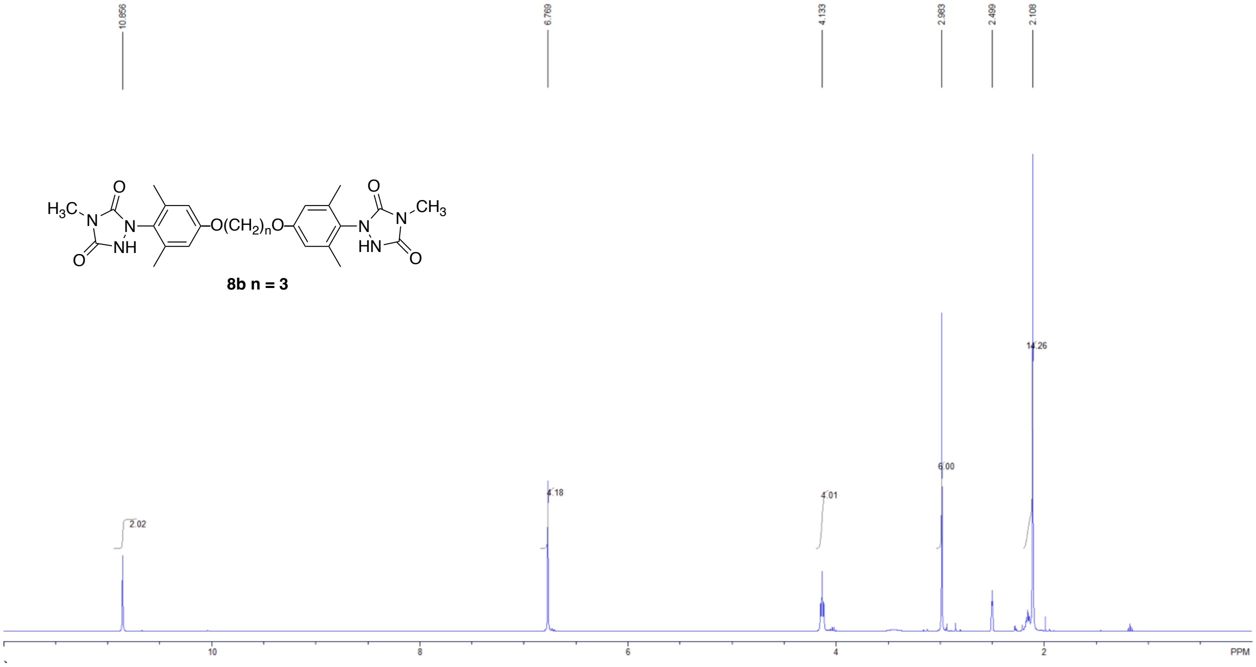

^{13}C{^{1}H} NMR Spectrum of Bisurazole 8b (DMSO-d$_6$, 100 MHz)

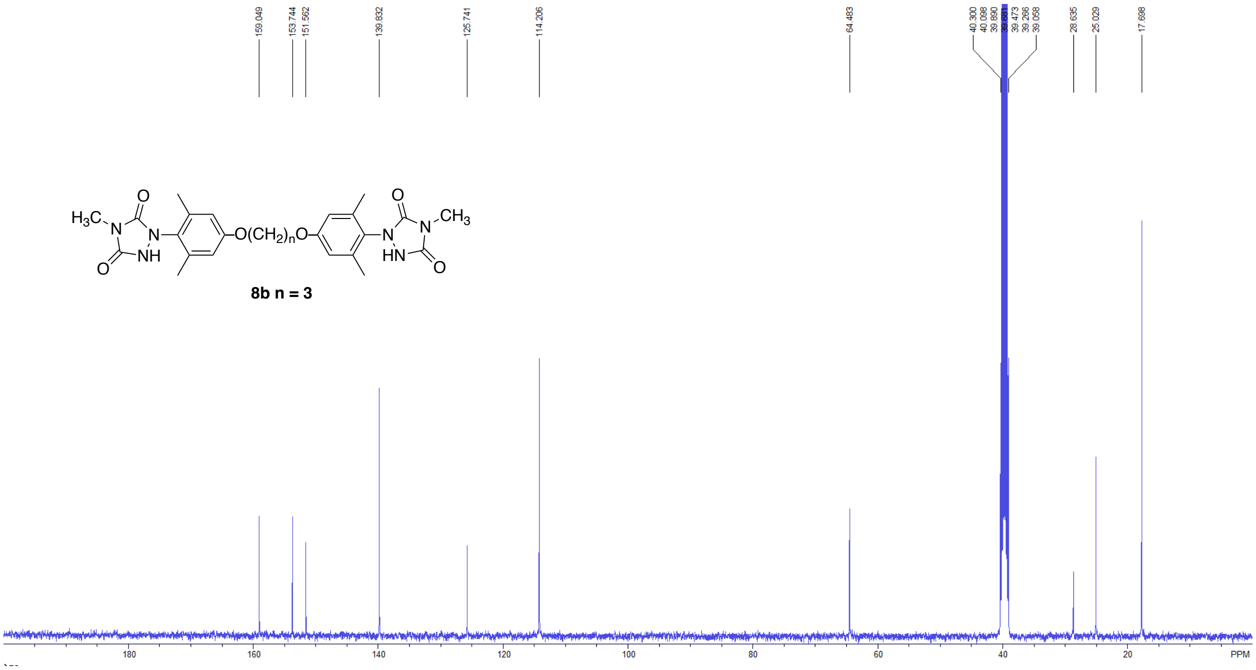

^{1}H NMR Spectrum of Dimer $4b_2$ ($CDCl_3$, 400 MHz, 25 °C)

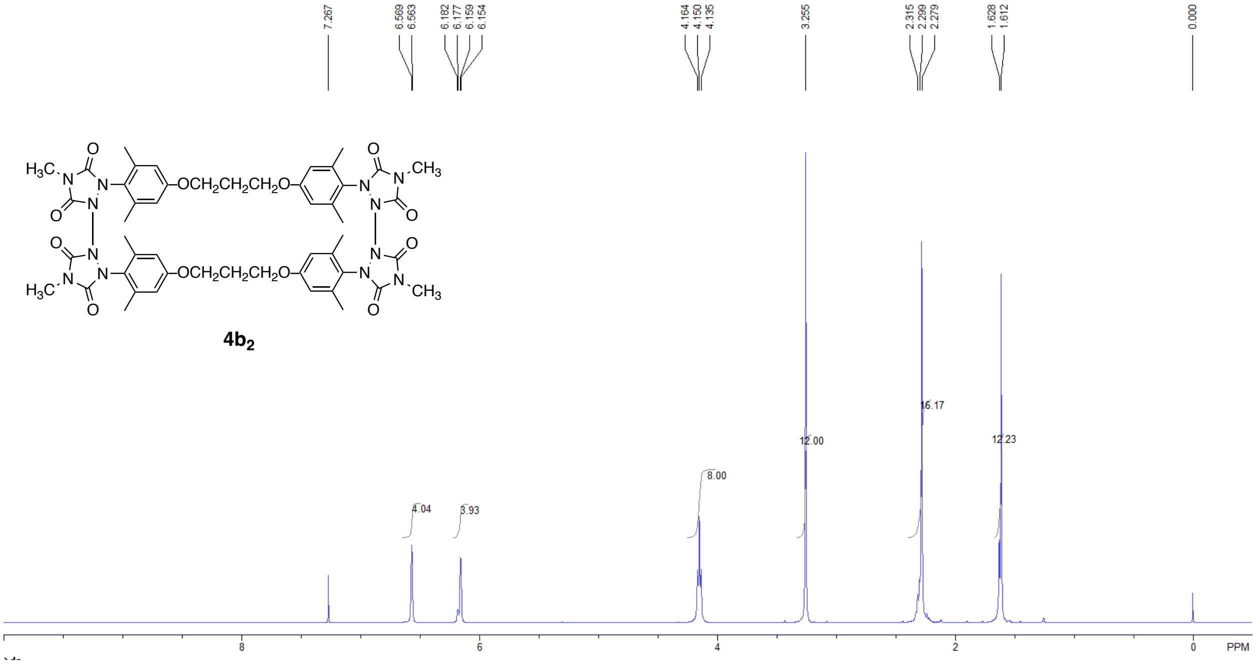

$^{13}C\{^{1}H\}$ NMR Spectrum of Dimer $4b_2$ ($CDCl_3$, 100 MHz, 25 °C)

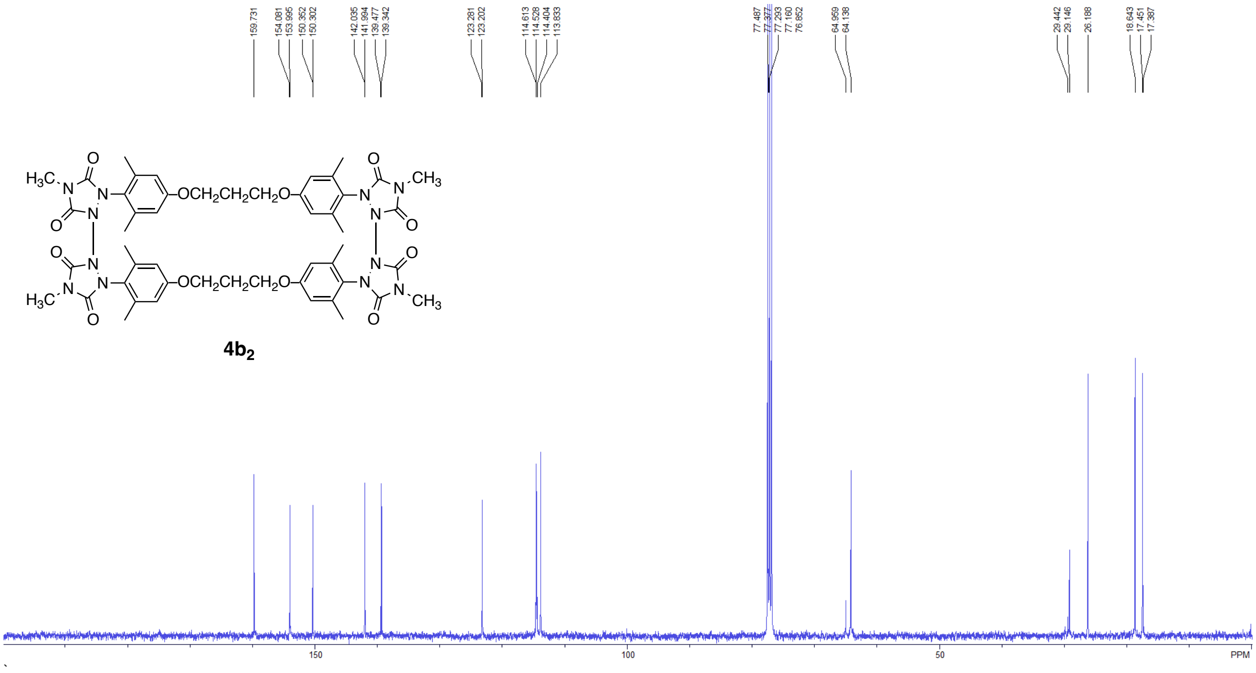

^{1}H NMR Spectrum of Dimer $4b_2$ ($CDCl_3$, 400 MHz, 60 °C)

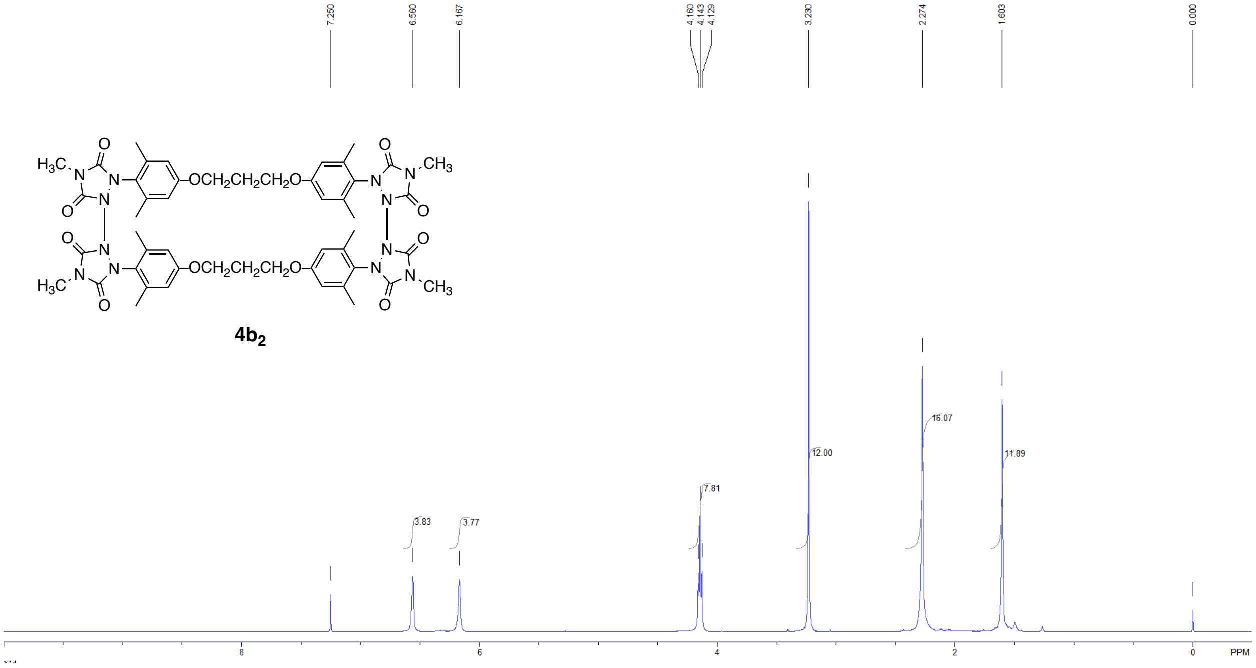

$^{13}C\{^{1}H\}$ NMR Spectrum of Dimer $4b_2$ ($CDCl_3$, 100 MHz, 60 °C)

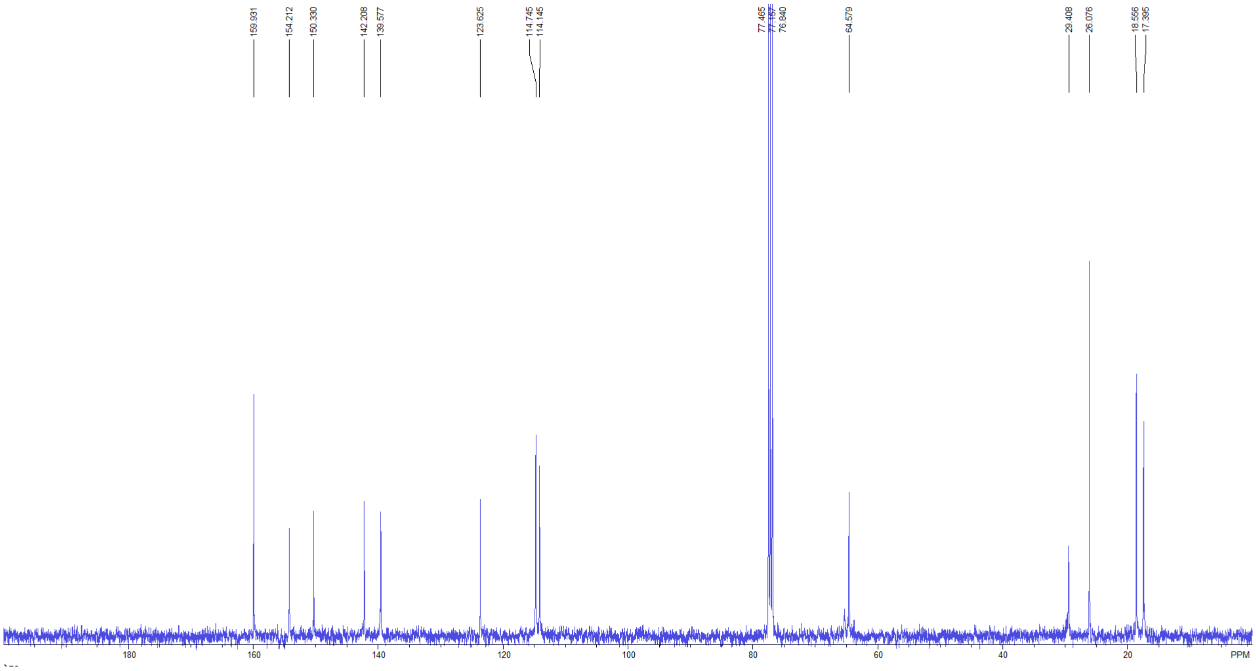

^{1}H NMR Spectrum of Compound 7c ($CDCl_3$, 400 MHz)

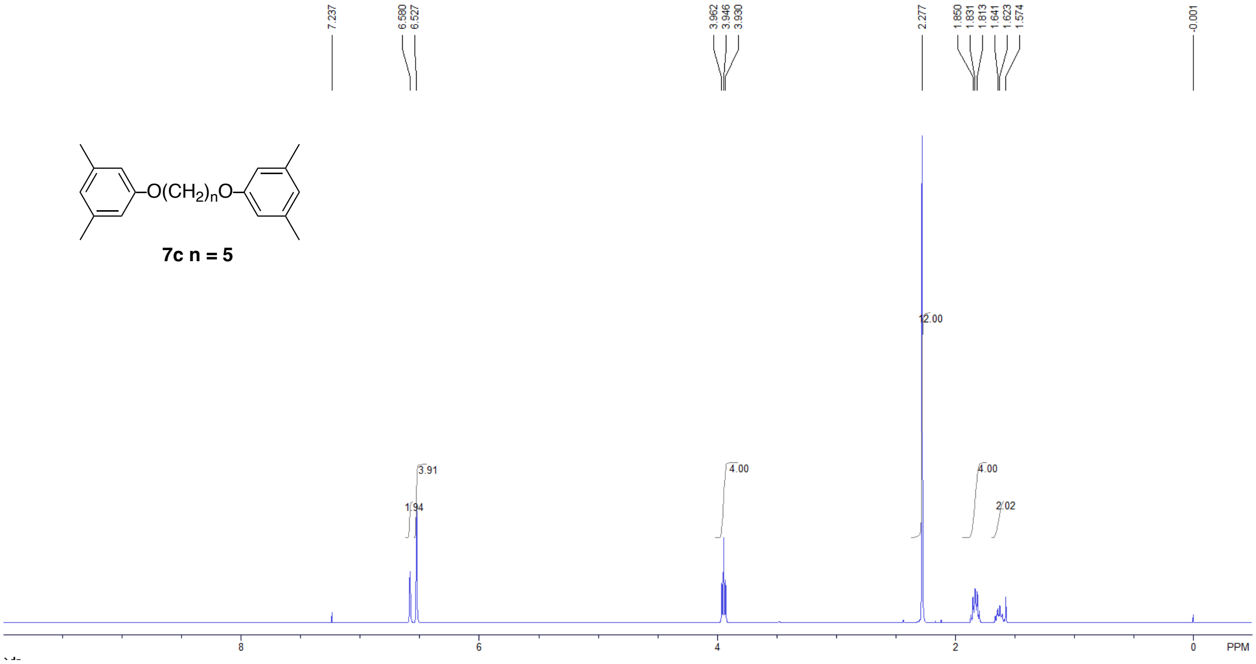

$^{13}C\{^{1}H\}$ NMR Spectrum of Compound 7c ($CDCl_3$, 100 MHz)

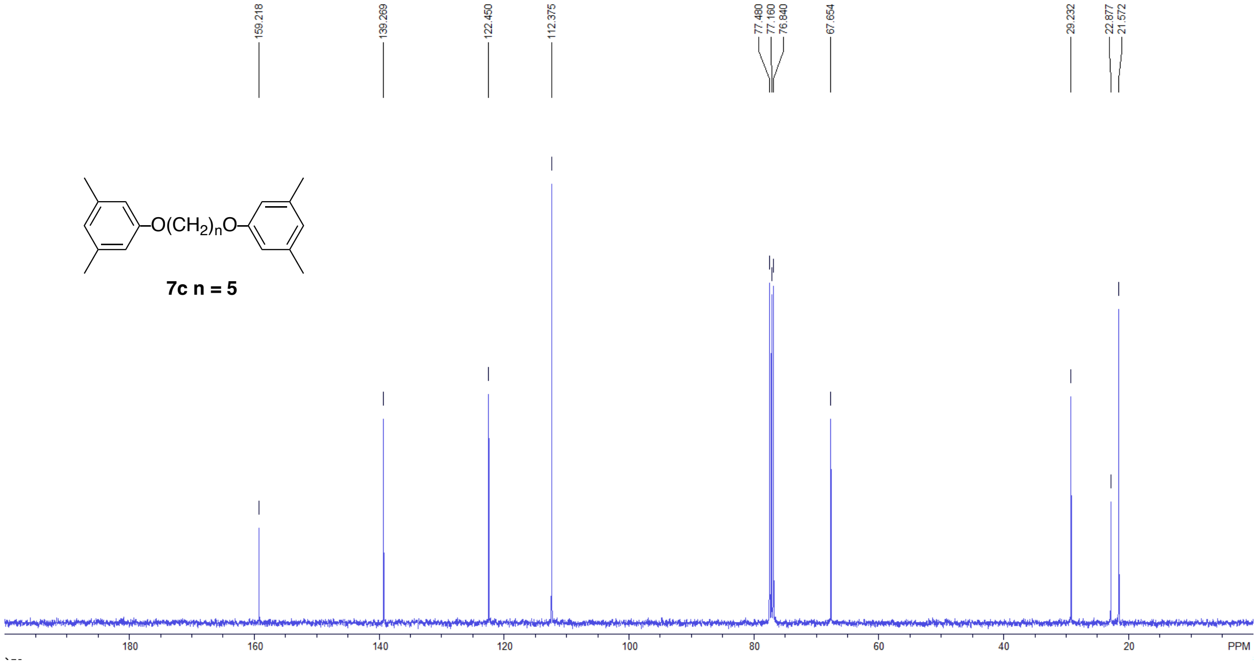

^{1}H NMR Spectrum of Bisurazole 8c (DMSO-d_6, 400 MHz)

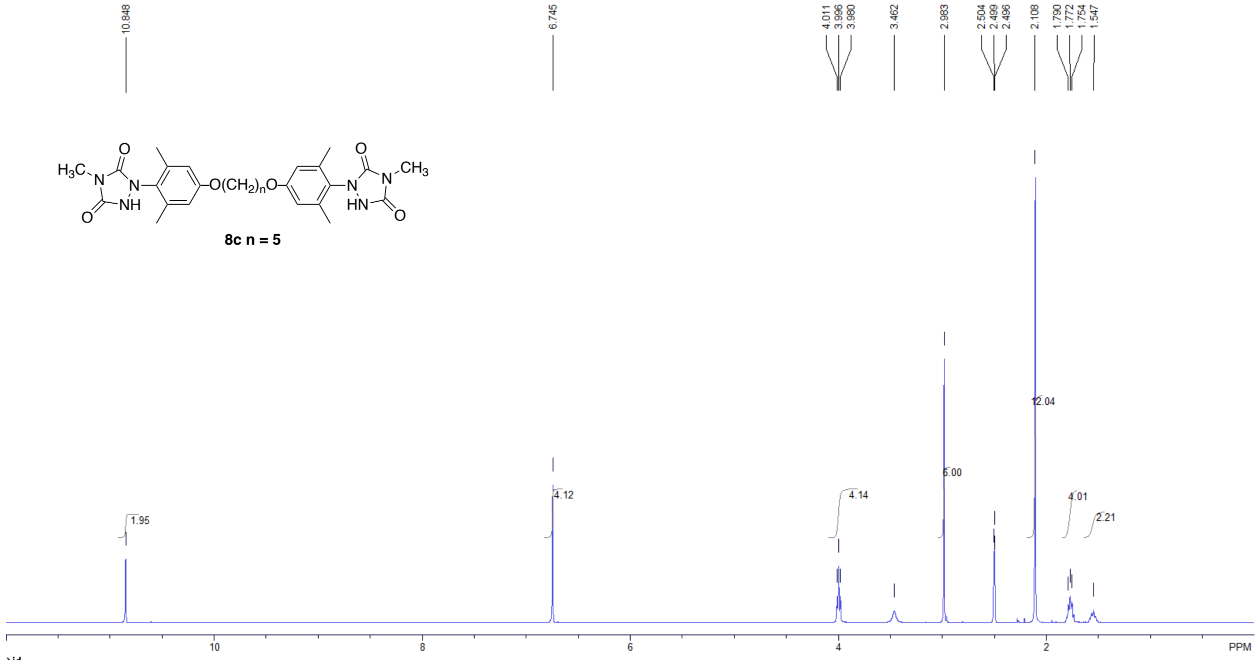

^{13}C{^{1}H} NMR Spectrum of Bisurazole 8c ($CDCl_3$, 100 MHz)

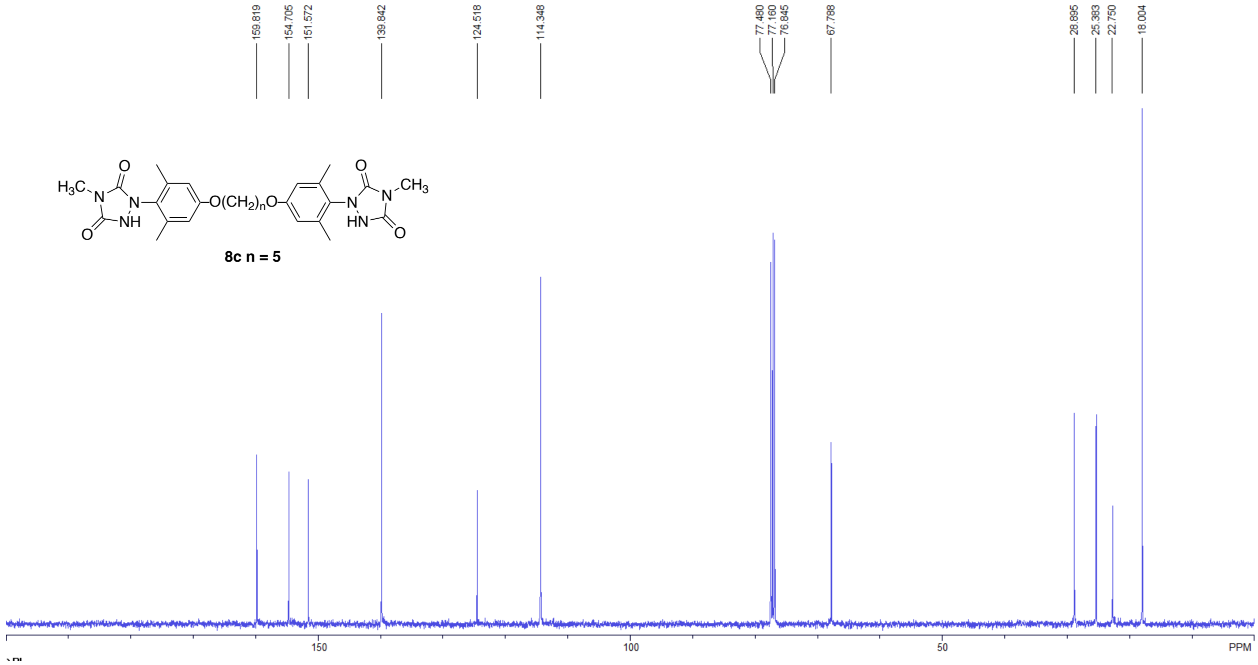

^{1}H NMR Spectrum of Monomer $4c_1$ ($CDCl_3$, 400 MHz)

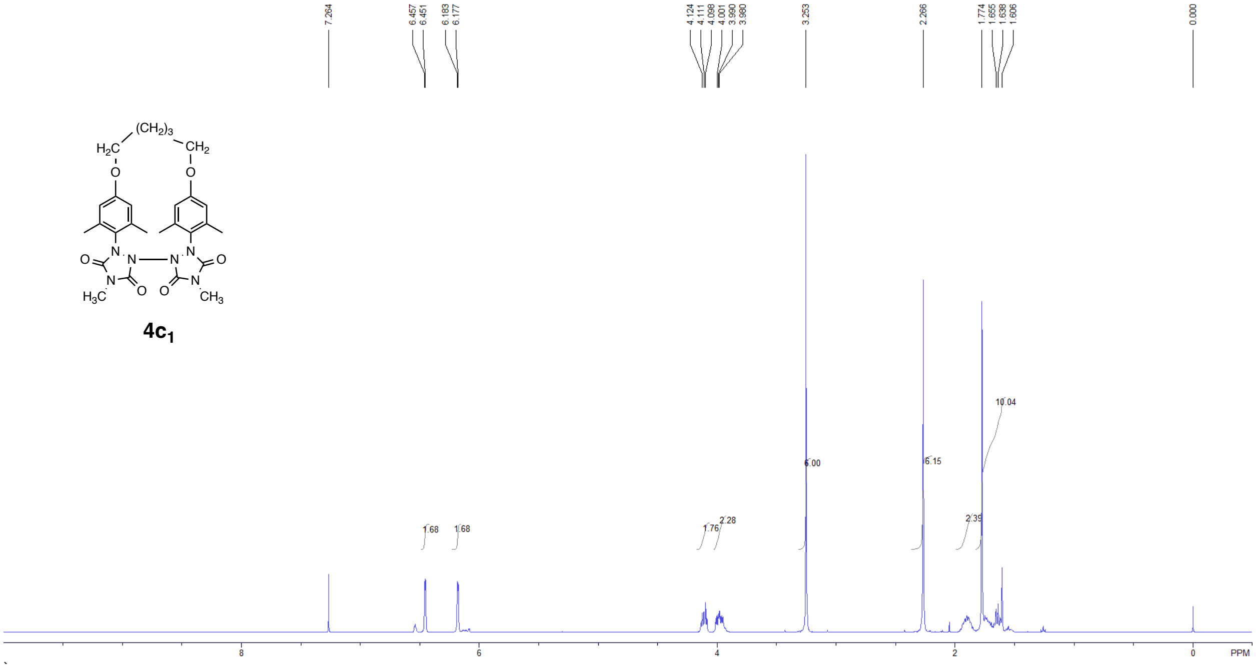

^{13}C{^{1}H} NMR Spectrum of Monomer $4c_1$ ($CDCl_3$, 100 MHz)

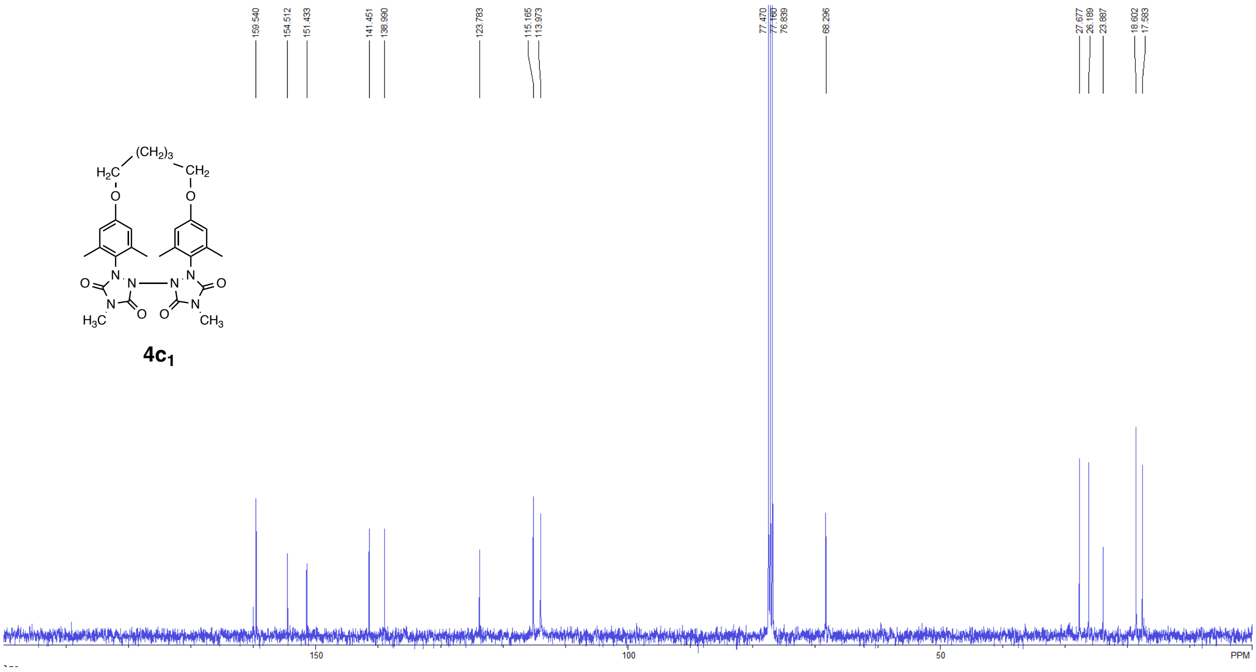

^1H NMR Spectrum of Dimer $4c_2$ ($CDCl_3$, 400 MHz)

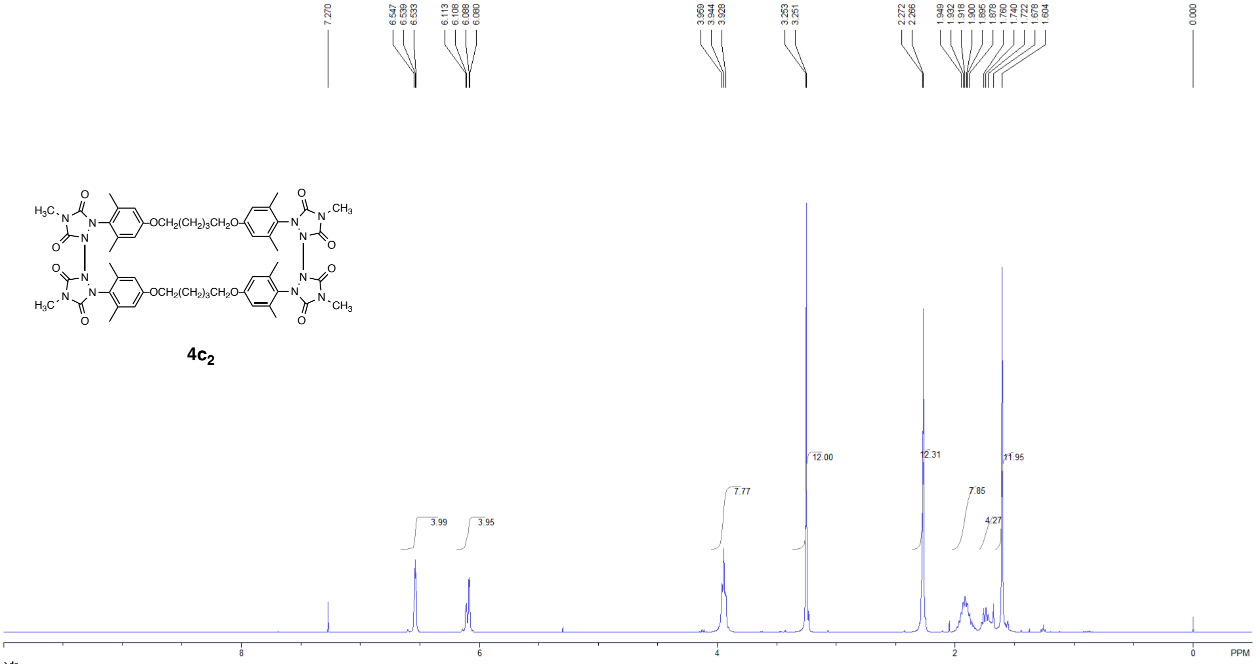

$^{13}C\{^1H\}$ NMR Spectrum of Dimer $4c_2$ ($CDCl_3$, 100 MHz)

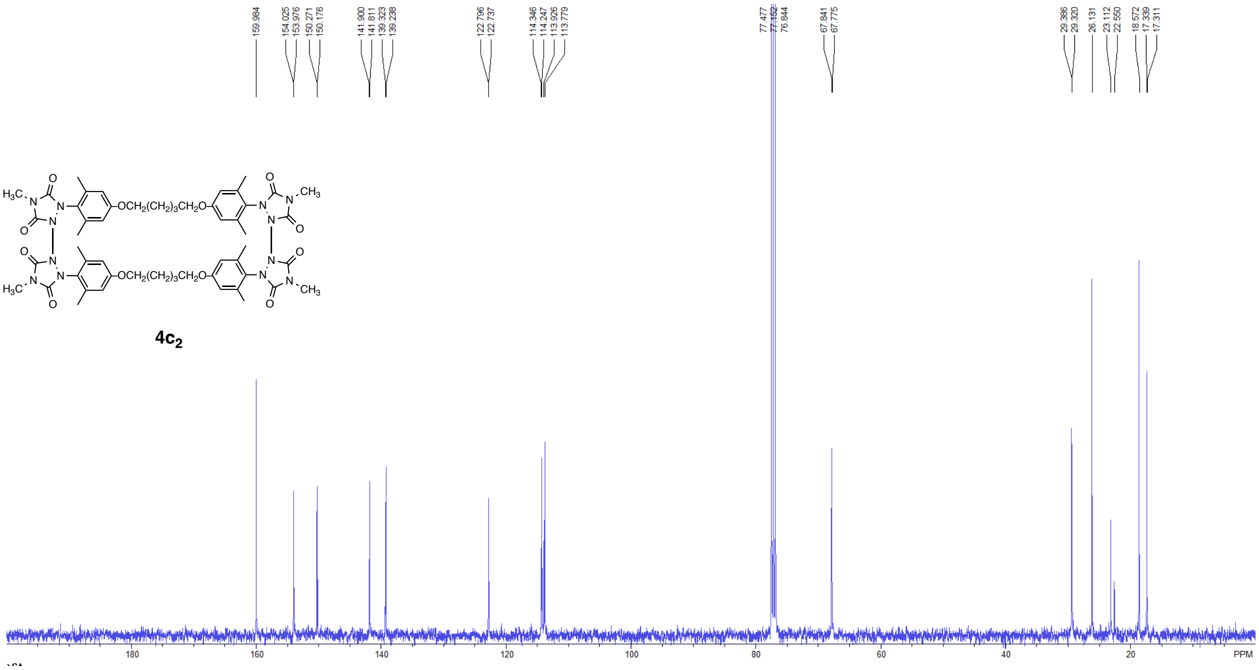

^{1}H NMR Spectrum of Compound 7d ($CDCl_3$, 400 MHz)

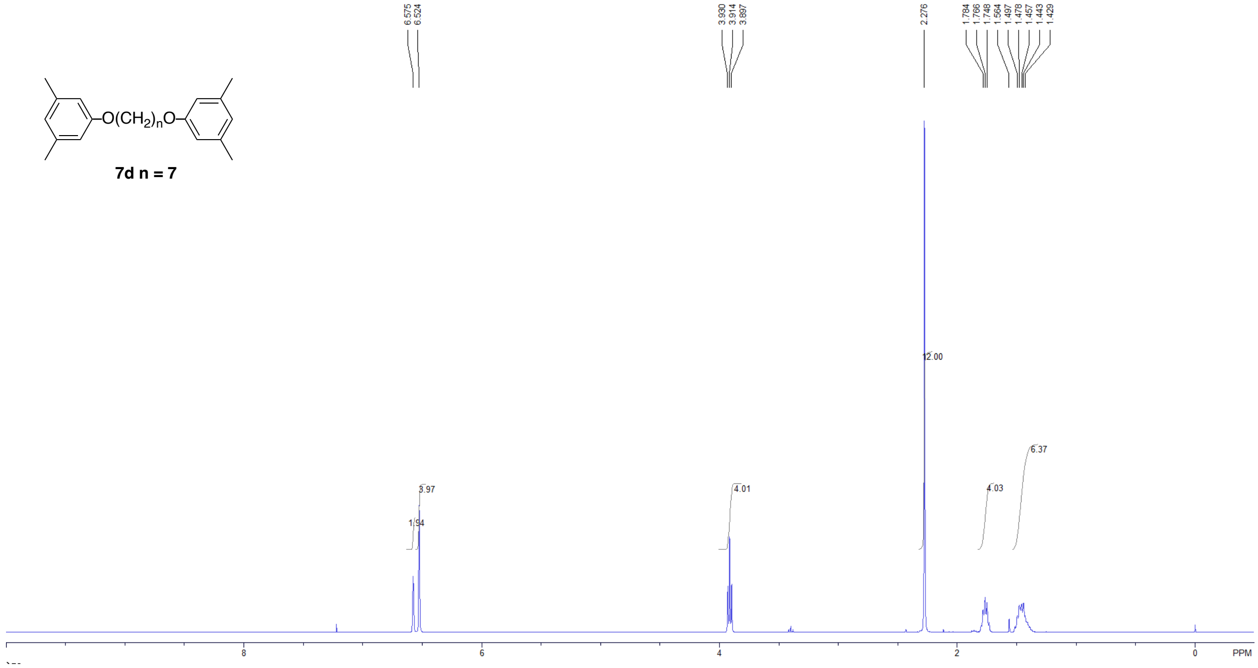

^{13}C{^{1}H} NMR Spectrum of Compound 7d ($CDCl_3$, 100 MHz)

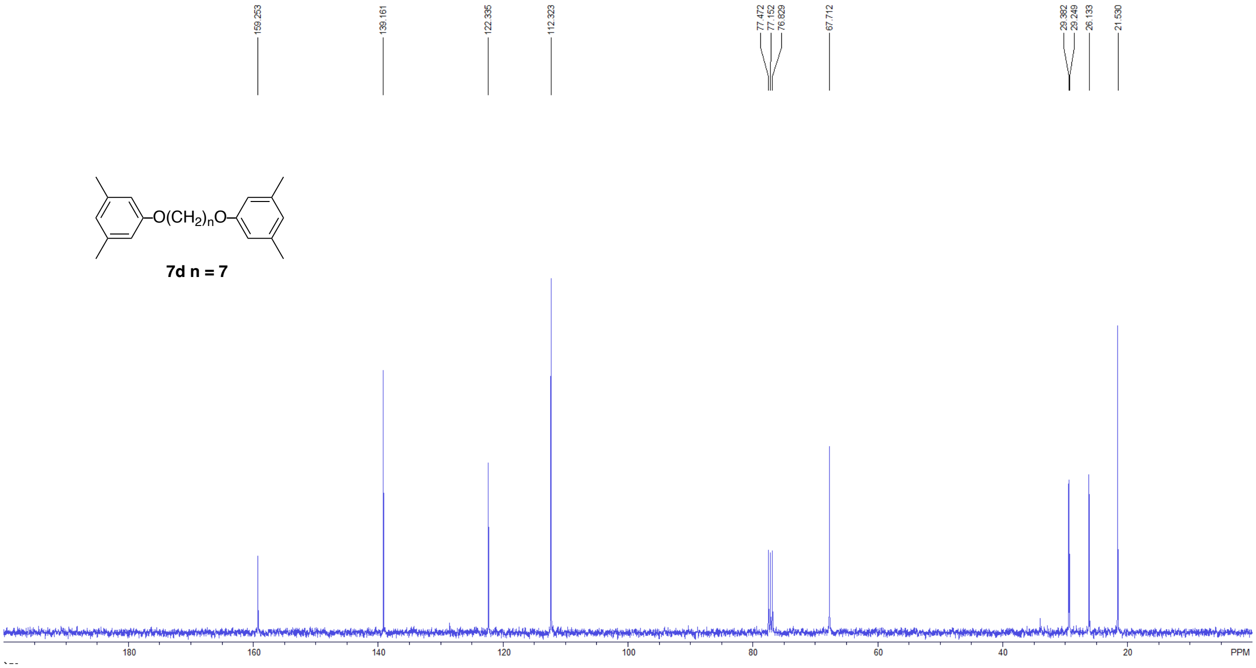

^{1}H NMR Spectrum of Compound 8d ($CDCl_3$, 400 MHz)

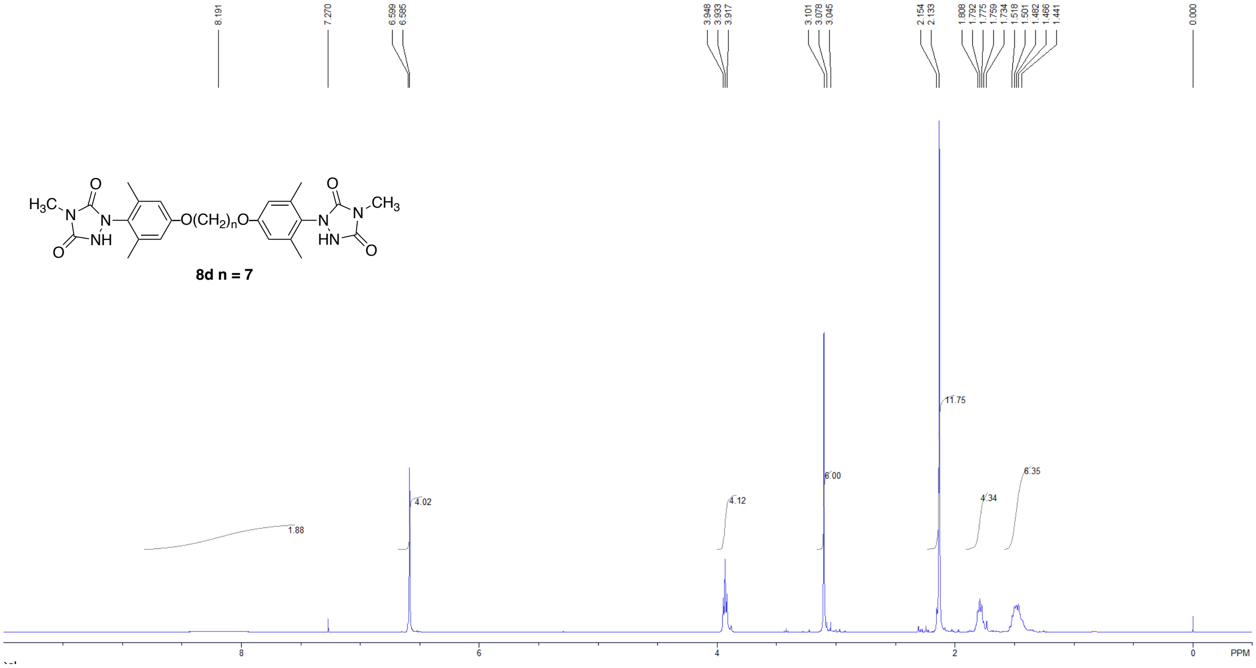

$^{13}C\{^{1}H\}$ NMR Spectrum of Compound 8d ($CDCl_3$, 100 MHz)

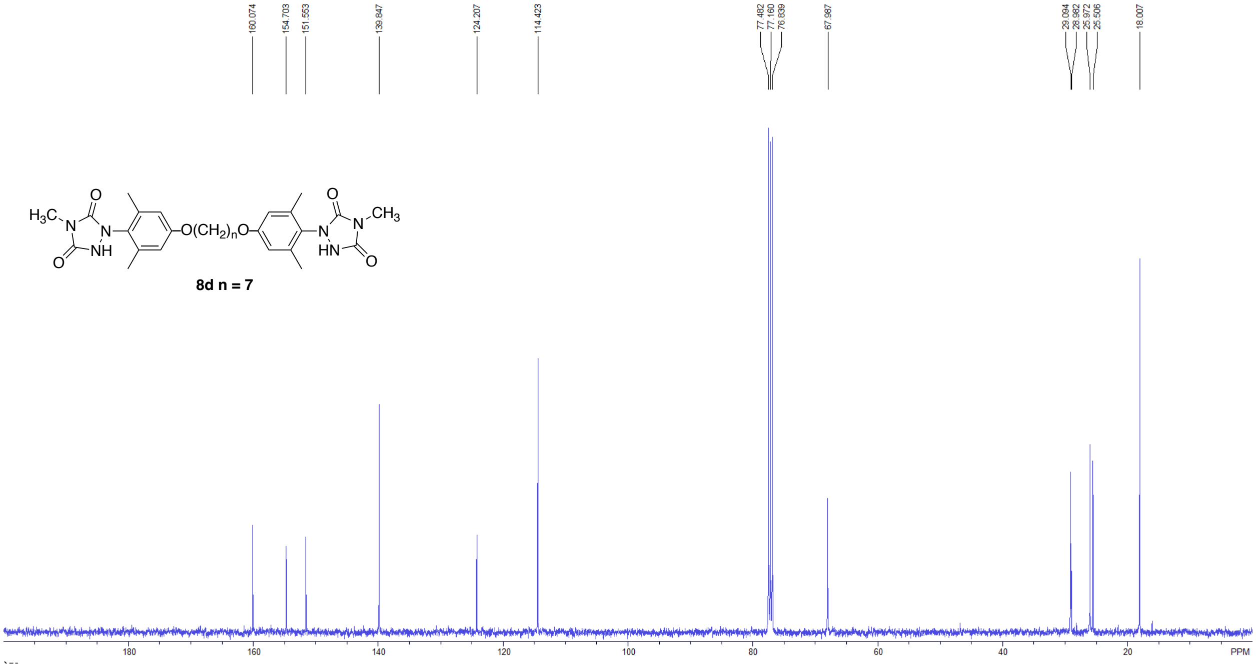

^{1}H NMR Spectrum of Monomer 4d$_1$ (CDCl$_3$, 400 MHz)

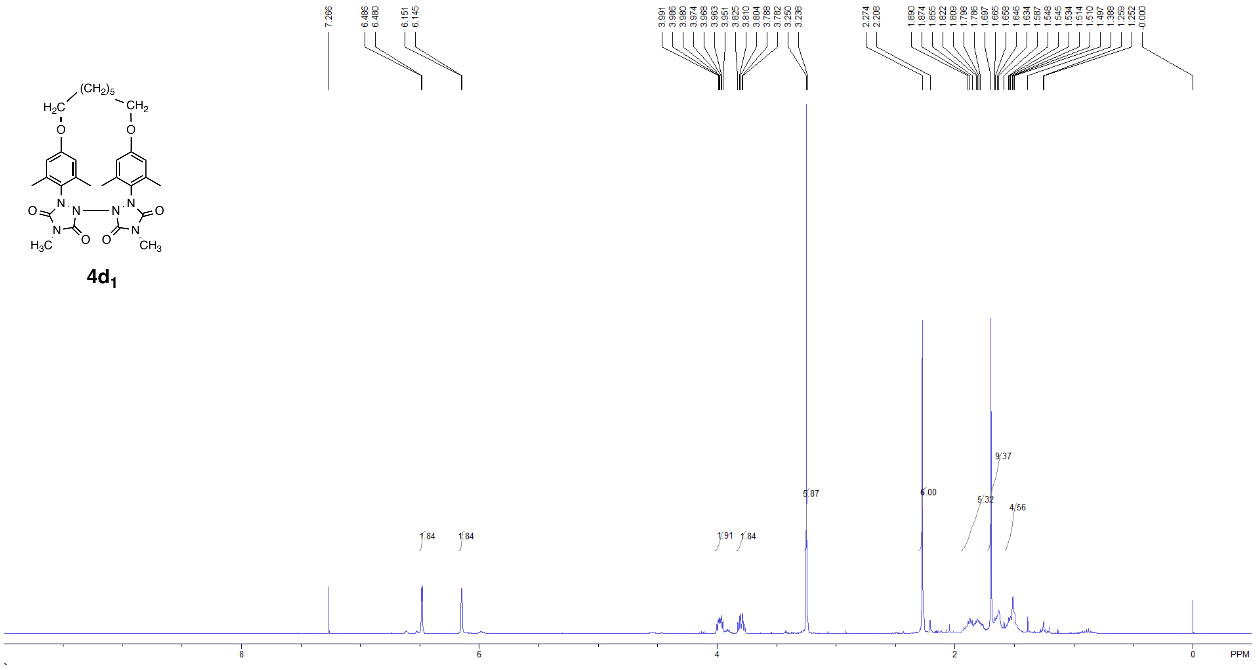

^{13}C{^{1}H} NMR Spectrum of Monomer 4d$_1$ (CDCl$_3$, 100 MHz)

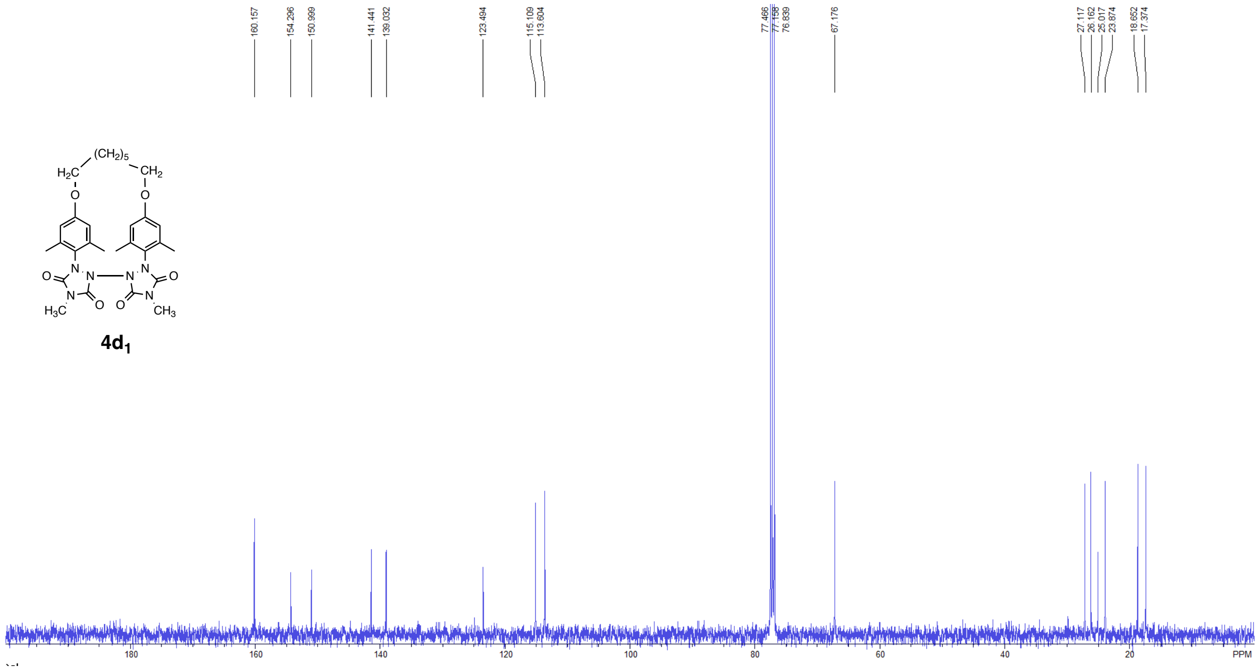

^{1}H NMR Spectrum of Dimer $4d_2$ ($CDCl_3$, 400 MHz)

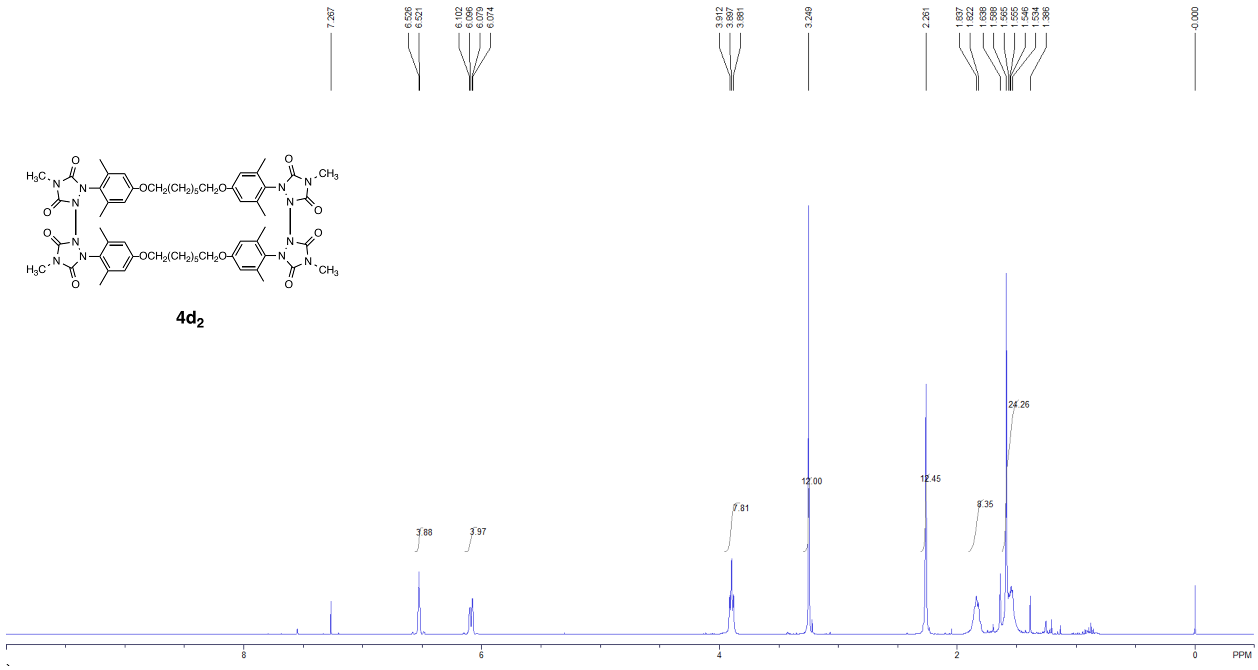

$^{13}C\{^{1}H\}$ NMR Spectrum of Dimer $4d_2$ ($CDCl_3$, 100 MHz)

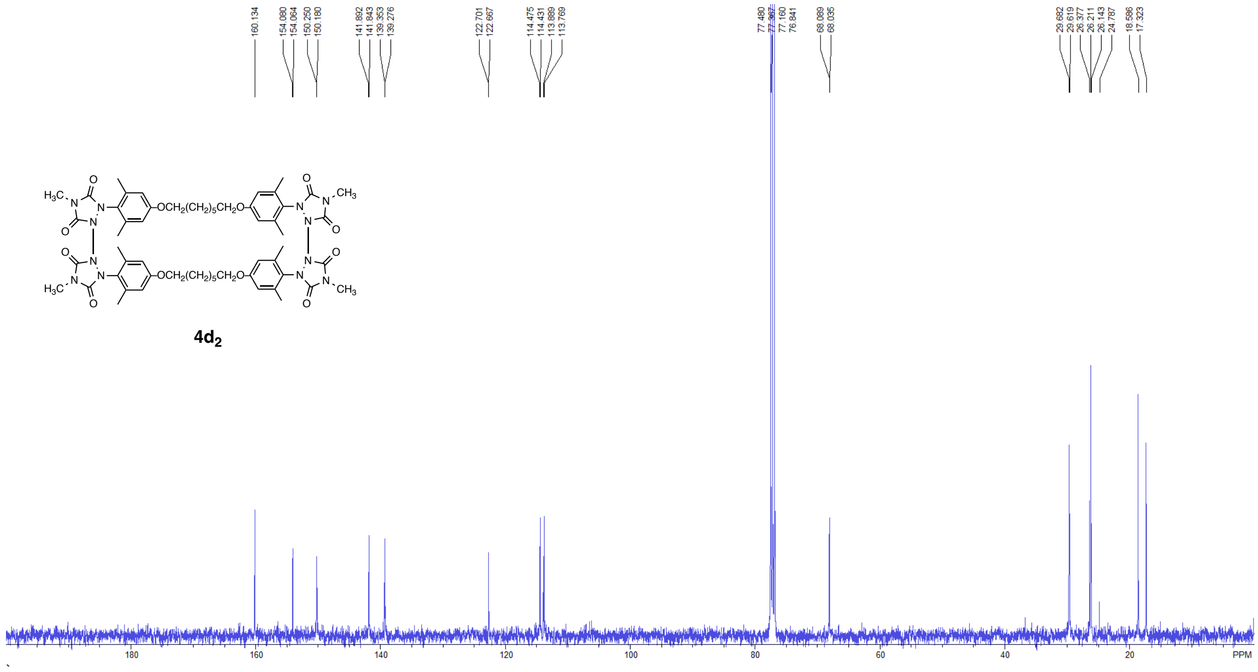

^{1}H NMR Spectrum of Compound 7e ($CDCl_3$, 400 MHz)

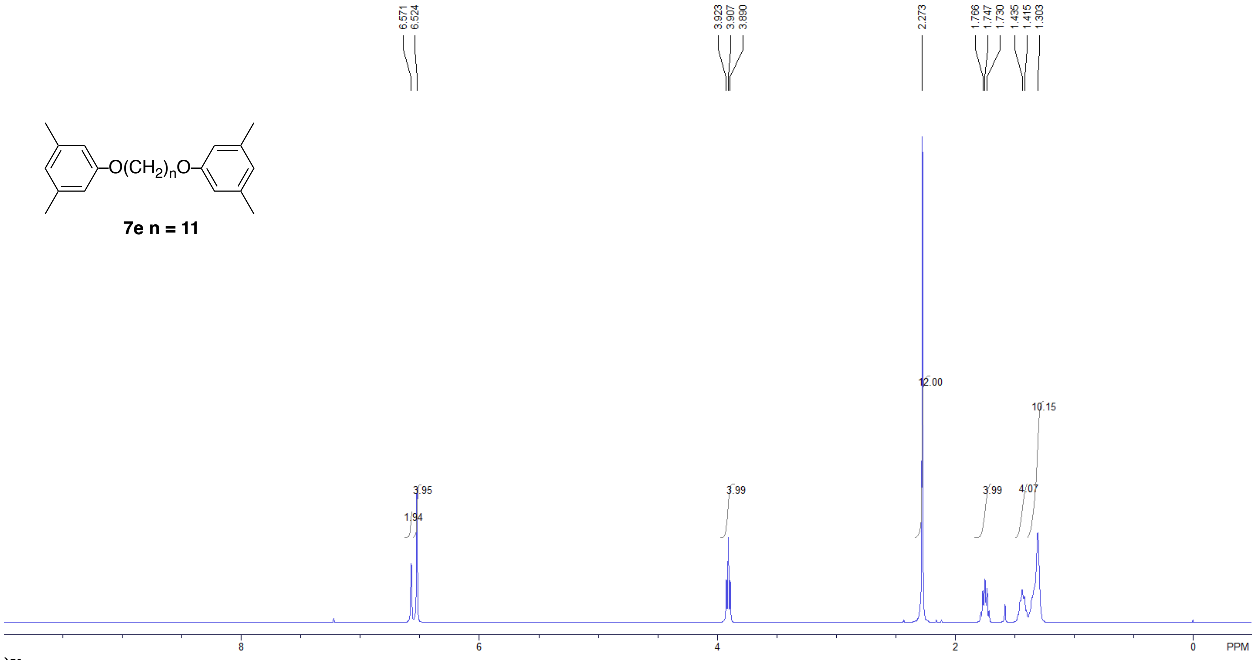

$^{13}C\{^{1}H\}$ NMR Spectrum of Compound 7e ($CDCl_3$, 100 MHz)

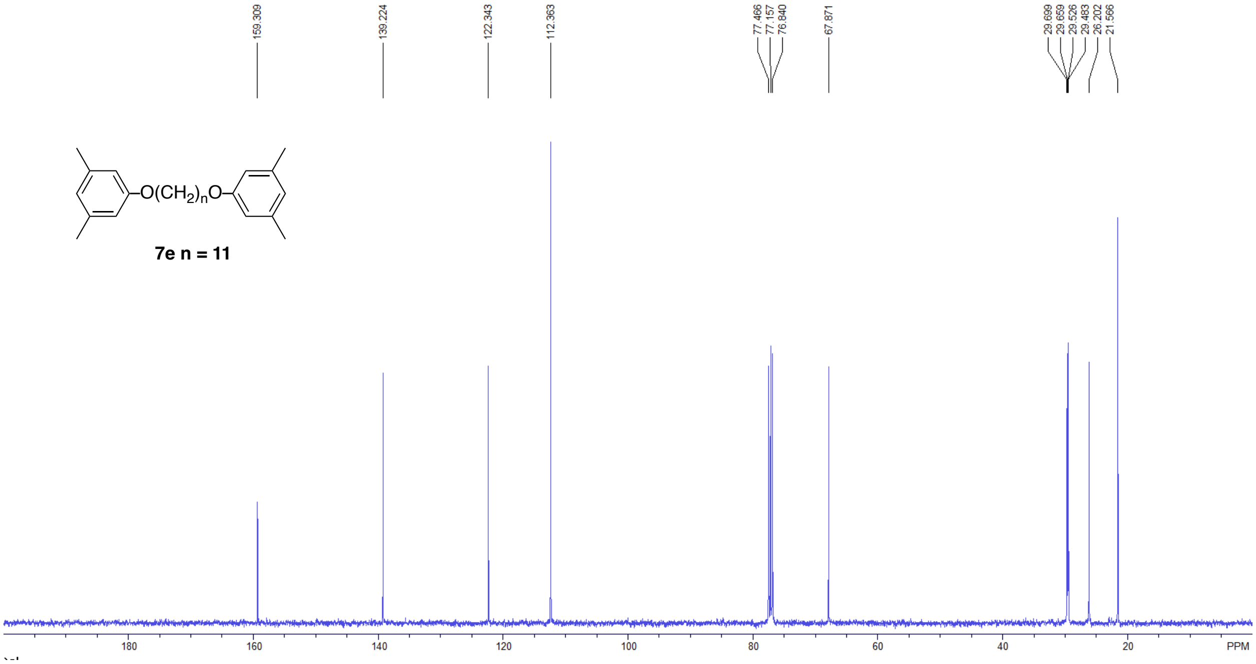

^1H NMR Spectrum of Compound 8e ($CDCl_3$, 400 MHz)

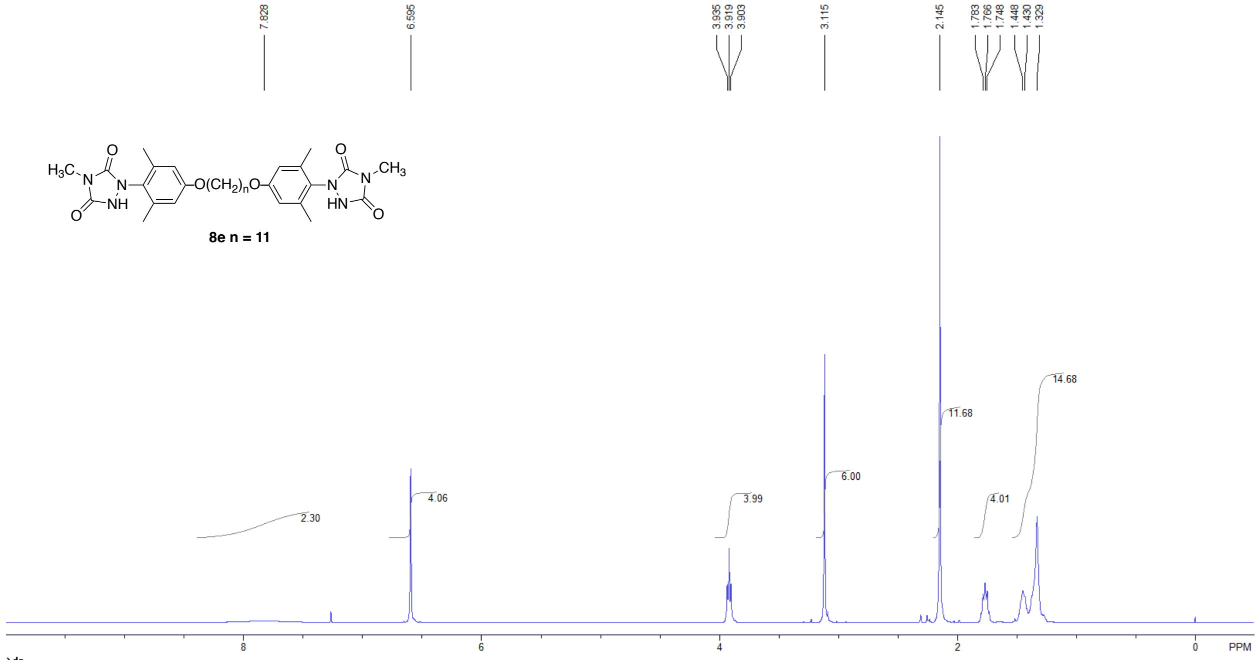

$^{13}C\{^1H\}$ NMR Spectrum of Compound 8e ($CDCl_3$, 100 MHz)

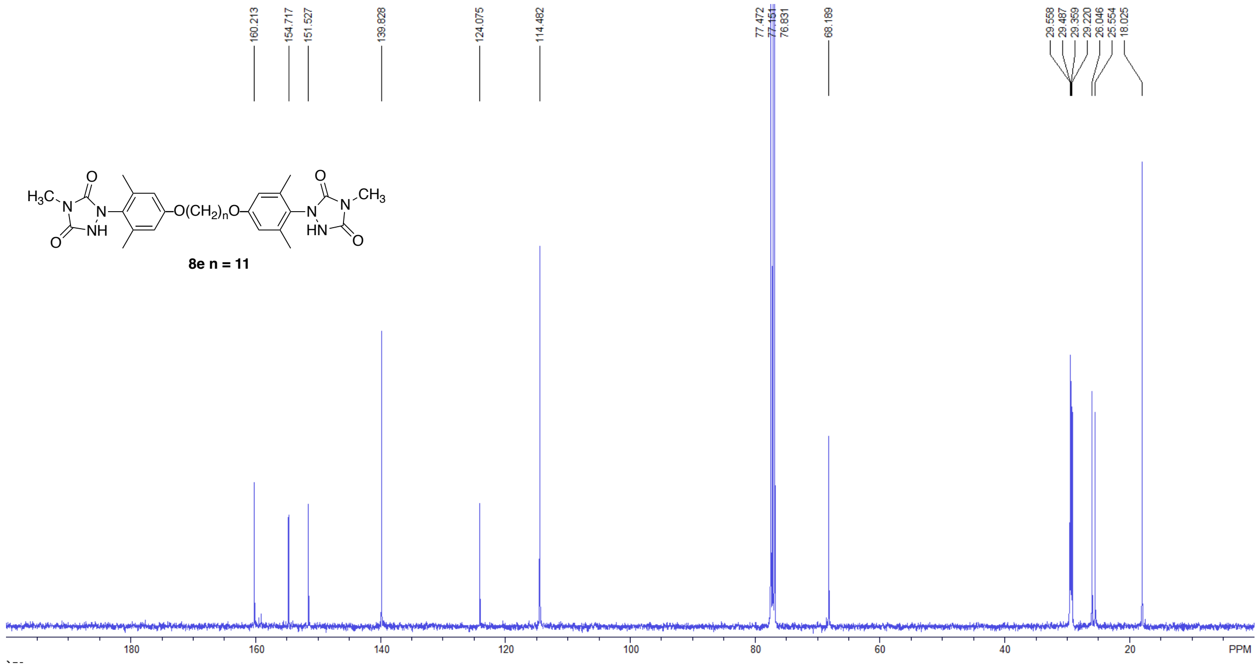

^{1}H NMR Spectrum of Monomer $4e_1$ ($CDCl_3$, 400 MHz)

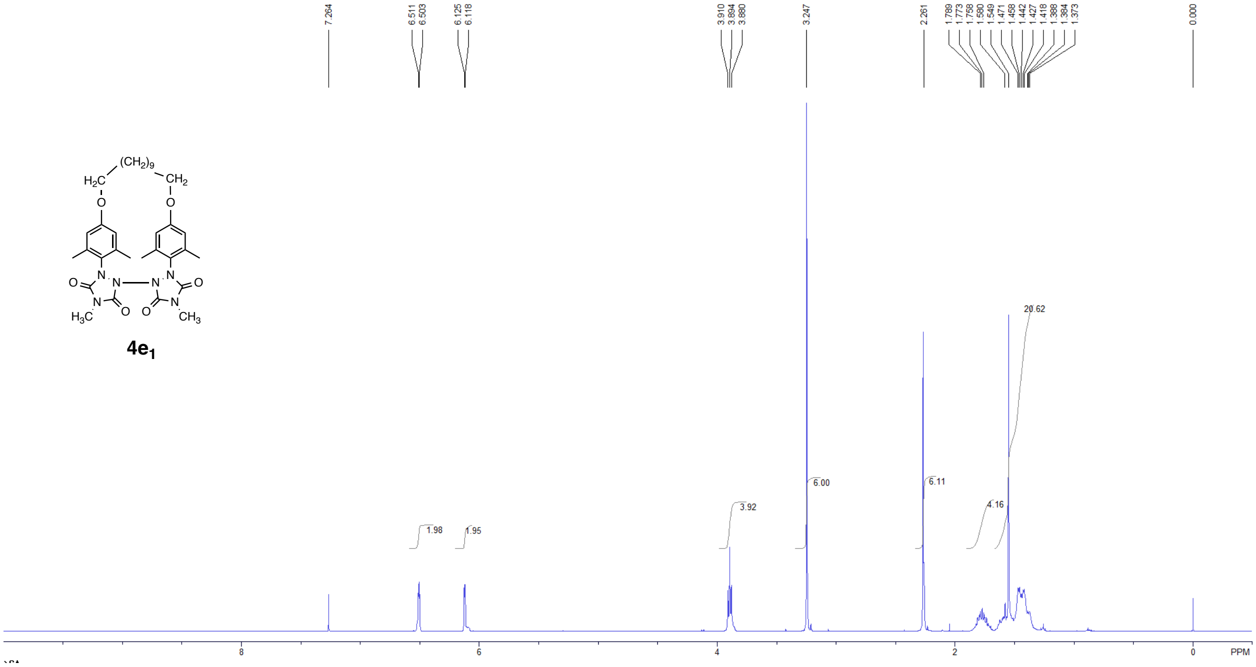

^{13}C{^{1}H} NMR Spectrum of Monomer $4e_1$ ($CDCl_3$, 100 MHz)

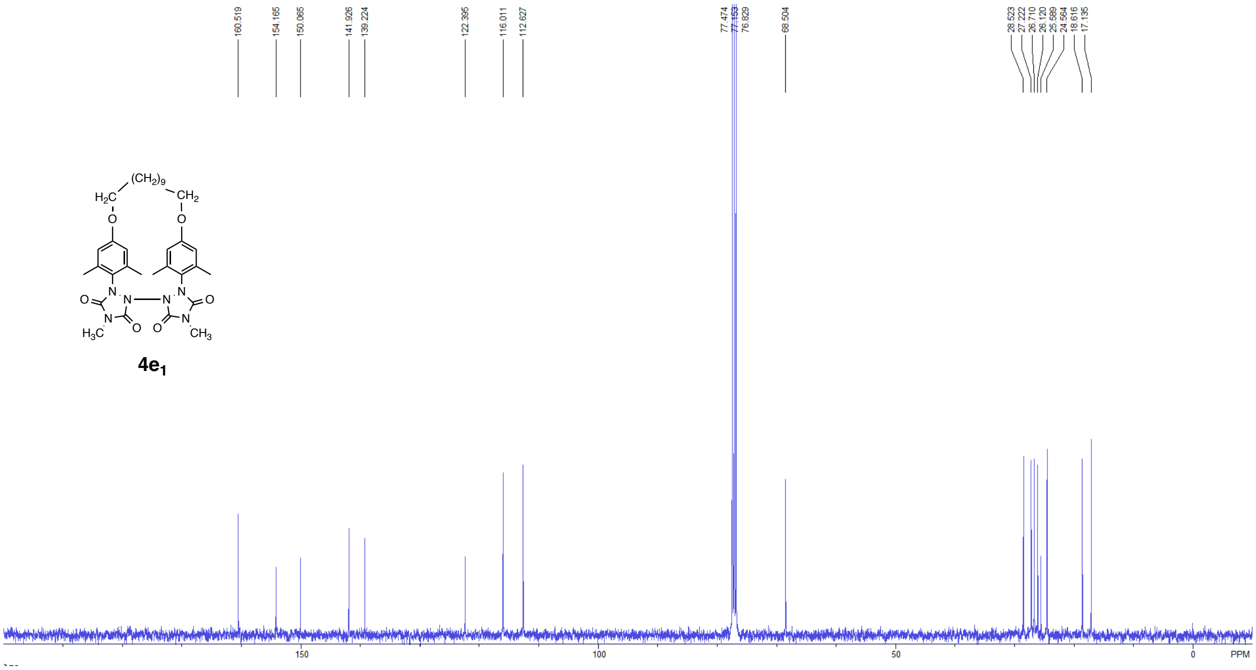

^{1}H NMR Spectrum of Dimer $4e_2$ ($CDCl_3$, 400 MHz)

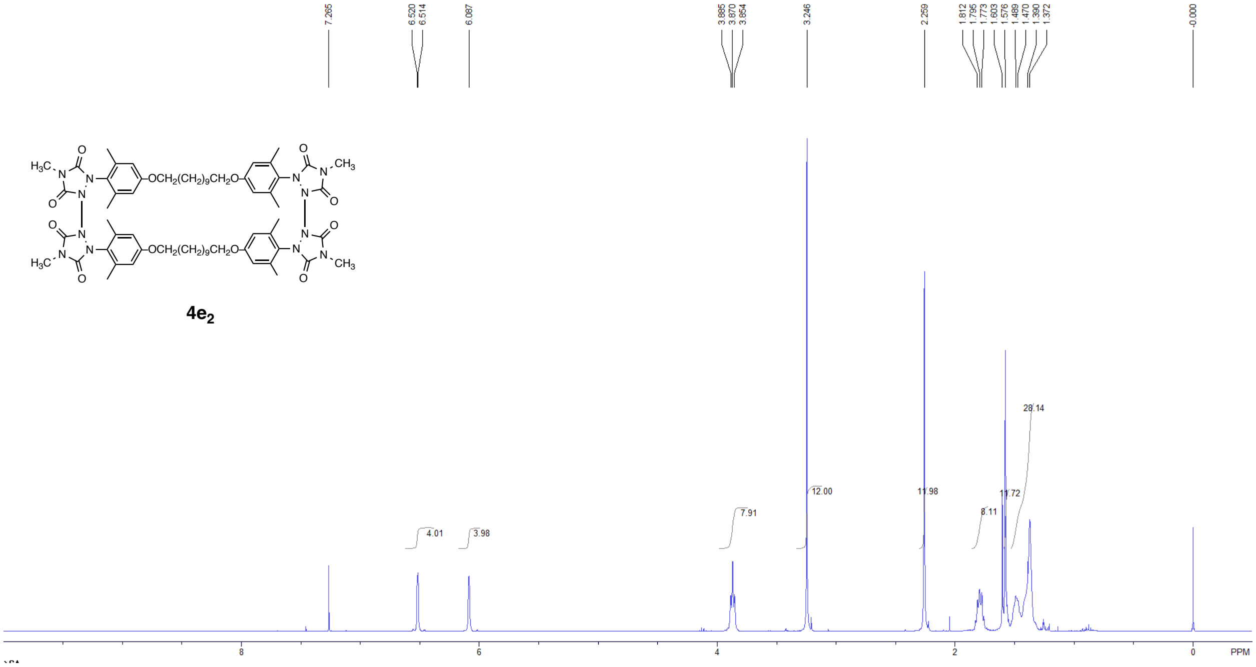

$^{13}C\{^{1}H\}$ NMR Spectrum of Dimer $4e_2$ ($CDCl_3$, 100 MHz)

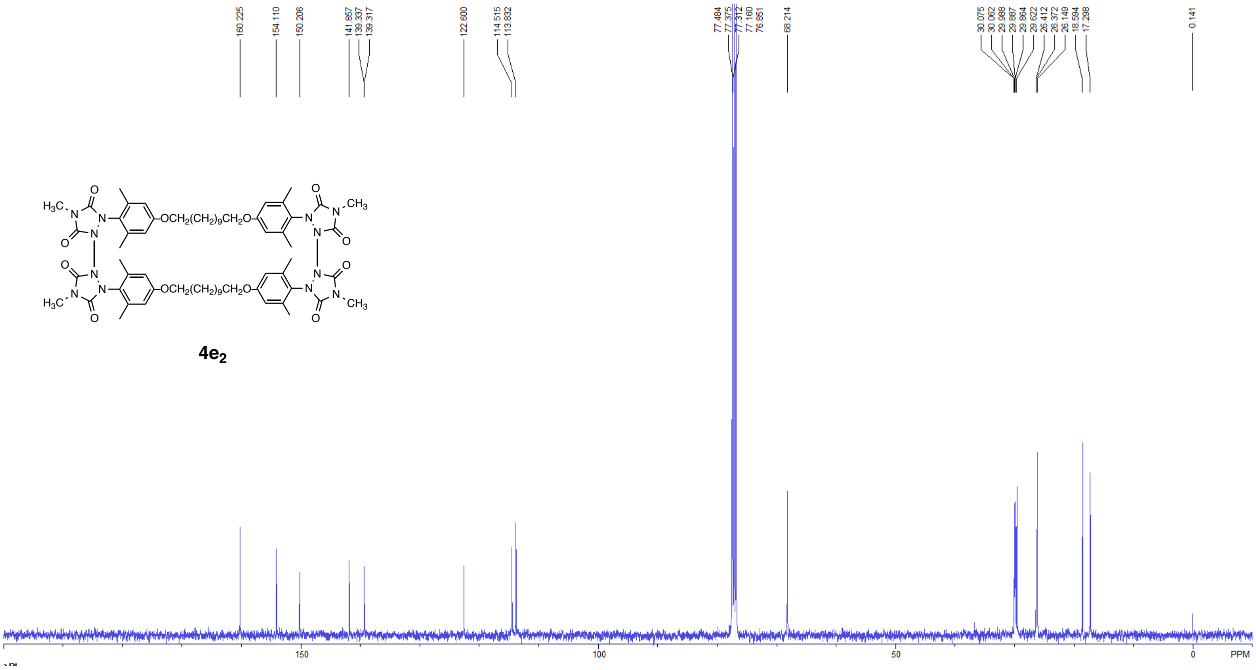

DOSY spectrum of a mixture of monomer $4c_1$ and dimer $4c_2$

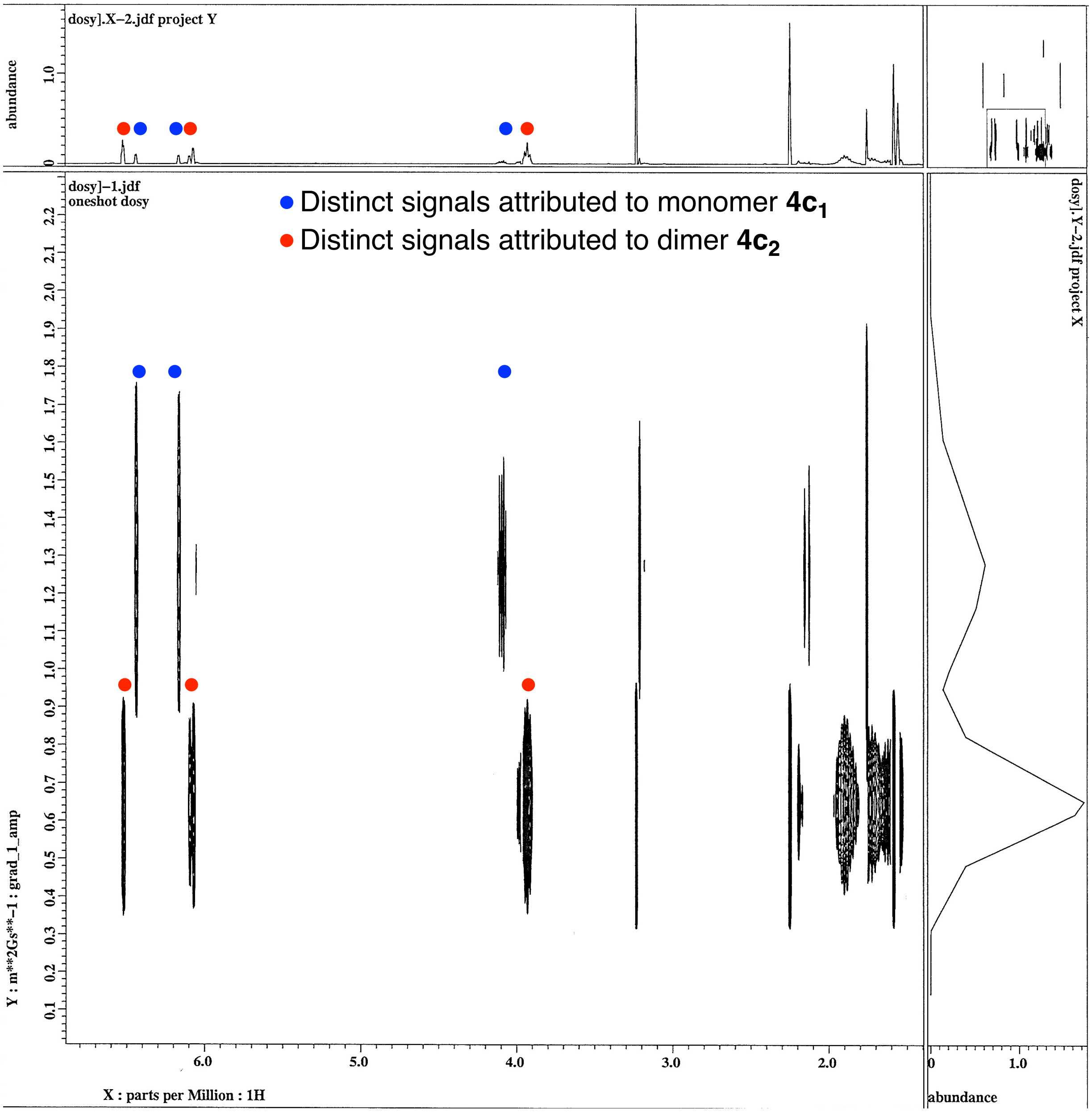

DOSY spectrum of a mixture of monomer $4e_1$ and dimer $4e_2$

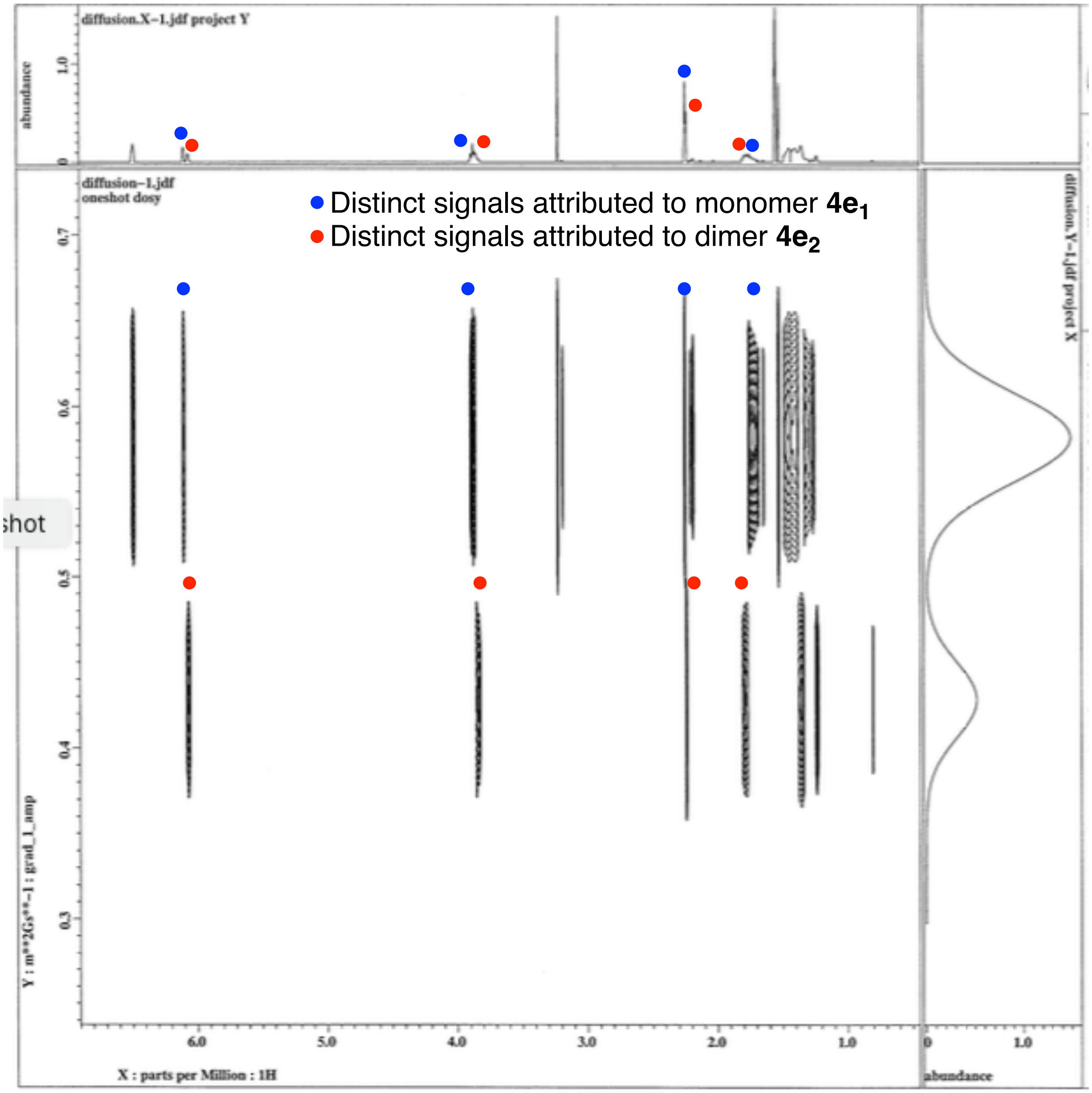

^{1}H NMR Spectra Used to Determine the Temperature Dependence of the Equilibrium Between Monomer 4c$_1$ and Dimer 4c$_2$

All spectra are collected as solutions in DMSO-d$_6$ at 400 MHz. The signals used for integrations are those at 6.65 ppm for dimer **4c$_2$** (4H) and 6.53 pm for monomer **4c$_1$** (2H). The percentage of **4c$_2$** was calculated according to ([1/2(integration of **4c$_2$** signal)/ [1/2(integration of **4c$_2$** signal)+1]

Temperature = 20 °C

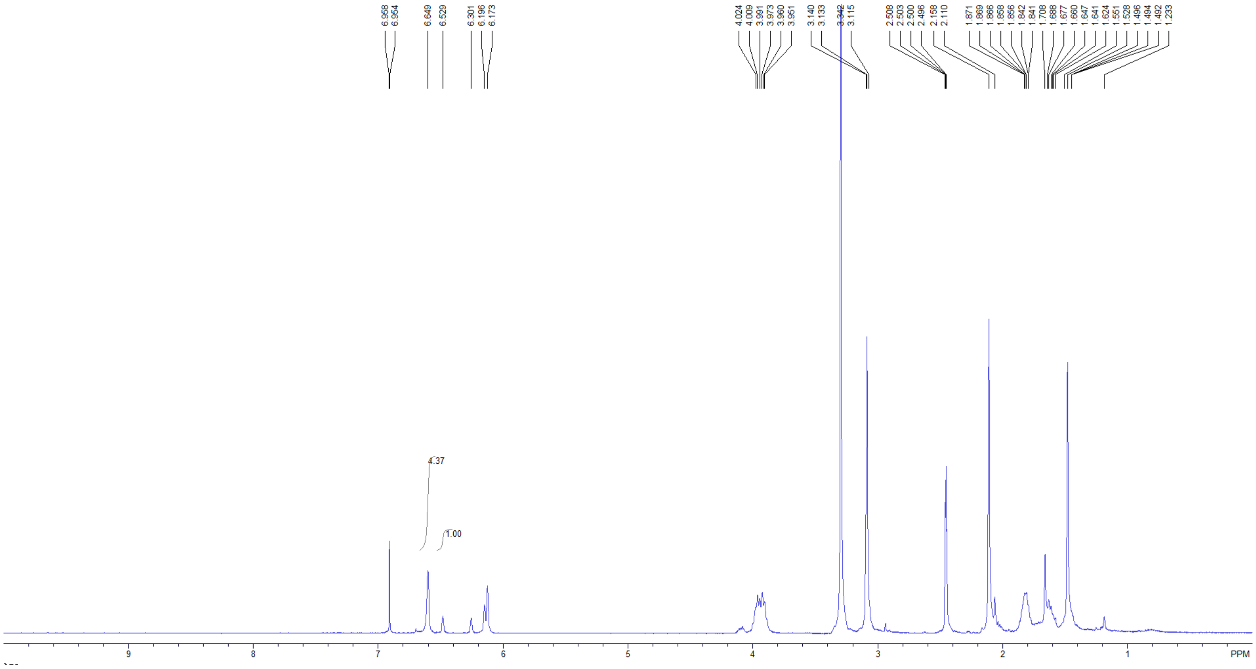

Temperature = 30 °C

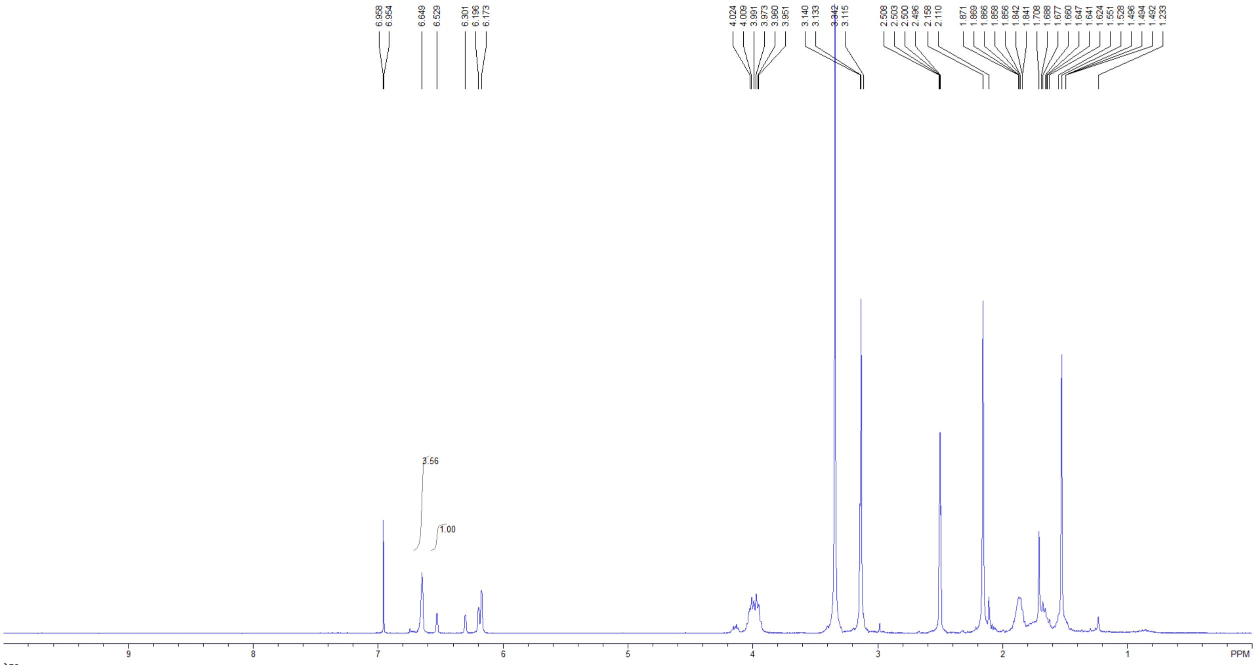

Temperature = 40 °C

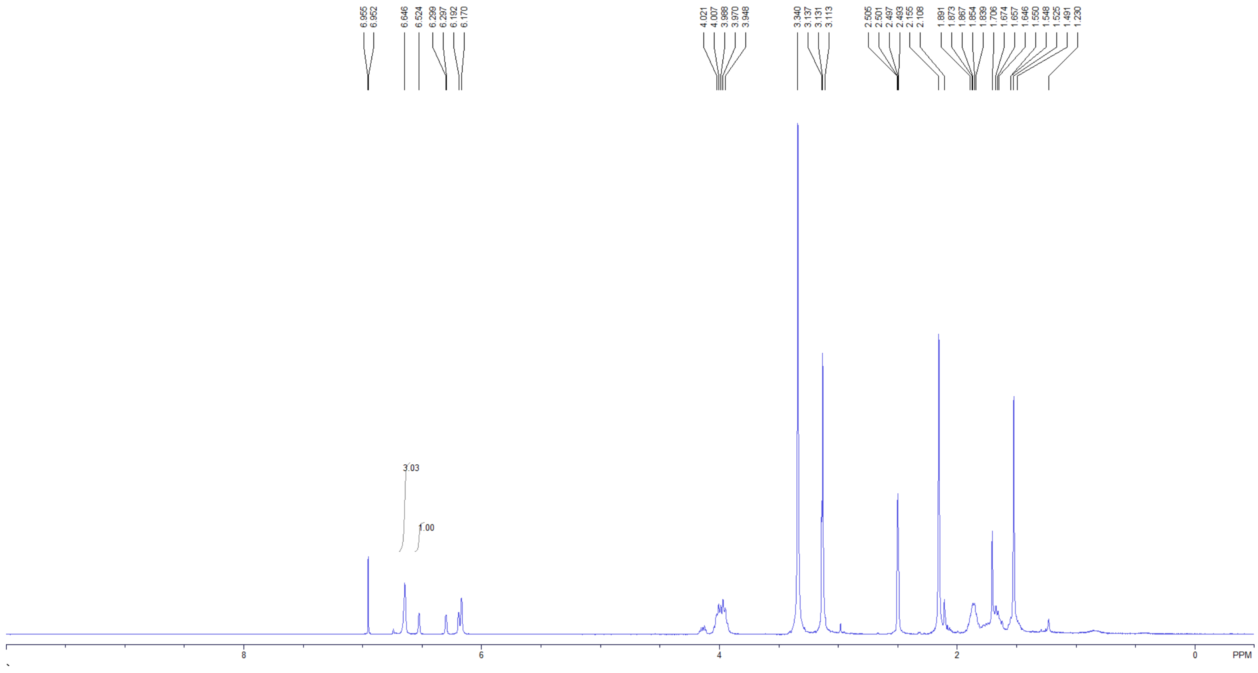

Temperature = 50 °C

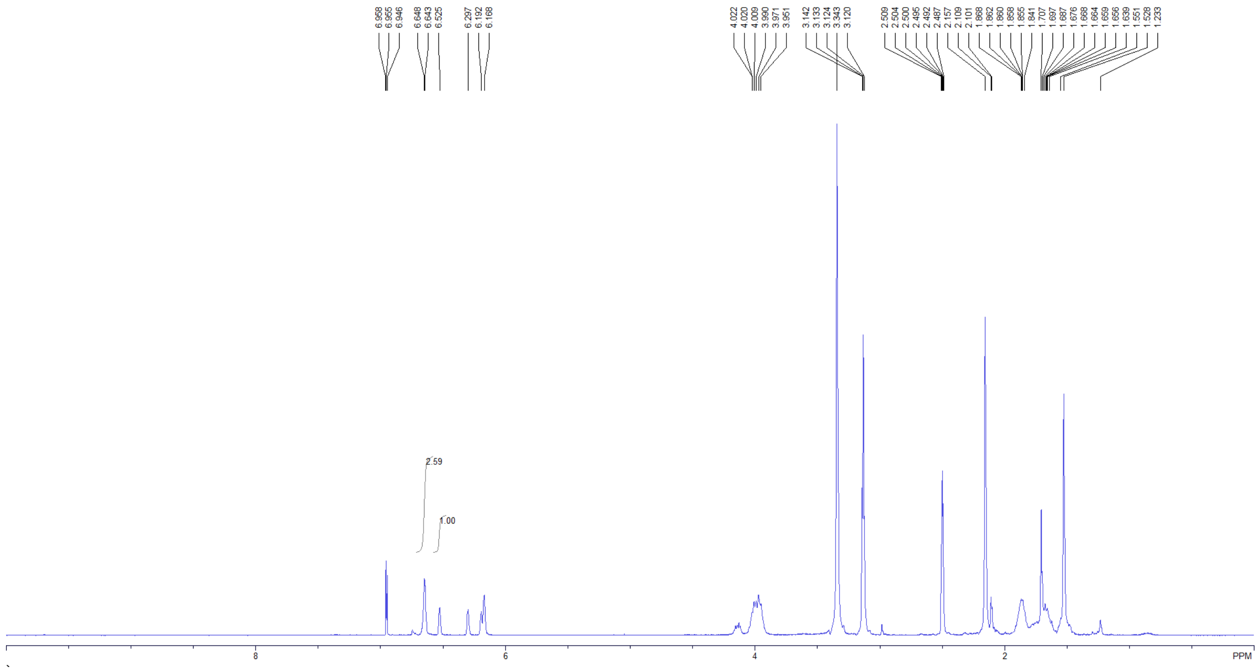

Temperature = 60 °C

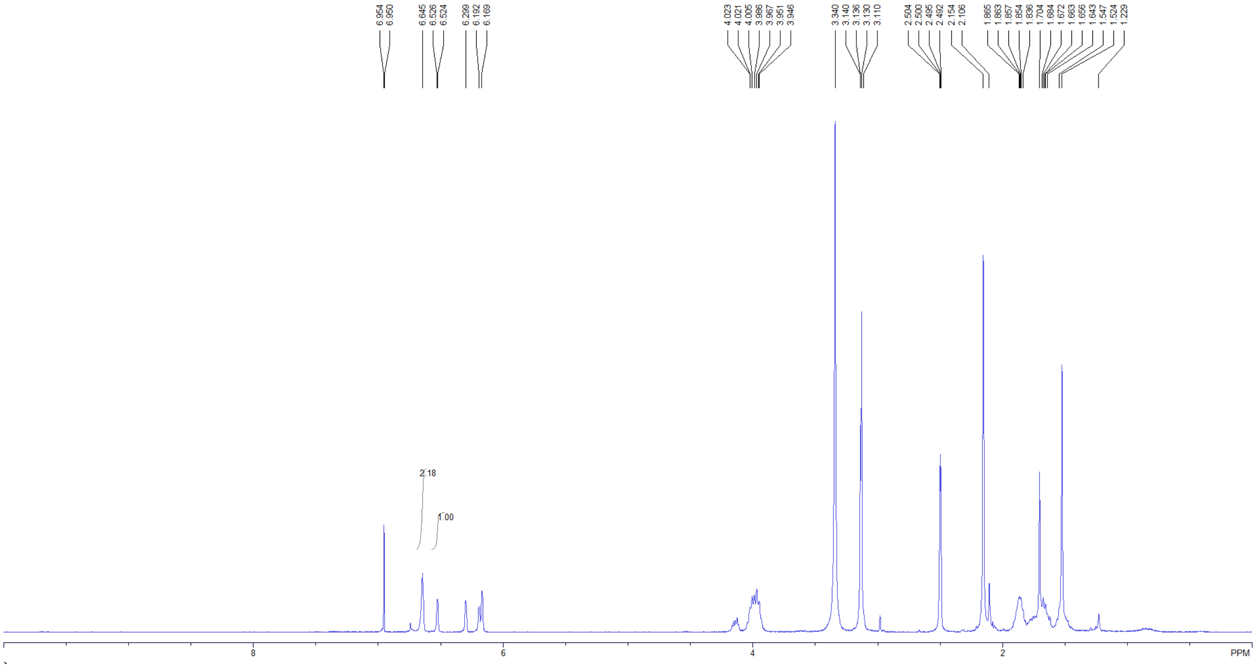

^{1}H NMR Spectra Used to Determine the Concentration Dependence of the Equilibrium Between Monomer 4c$_1$ and Dimer 4c$_2$

All spectra are collected as solutions in $CDCl_3$ at 400 MHz. The signals used for integrations are those at 6.65 ppm for dimer **4c$_2$** (4H) and 6.52 pm for monomer **4c$_1$** (2H). The percentage of **4c$_2$** was calculated according to ([1/2(integration of **4c$_2$** signal)/ [1/2(integration of **4c$_2$** signal)+1]

Initial Concentration = 62.9 mM

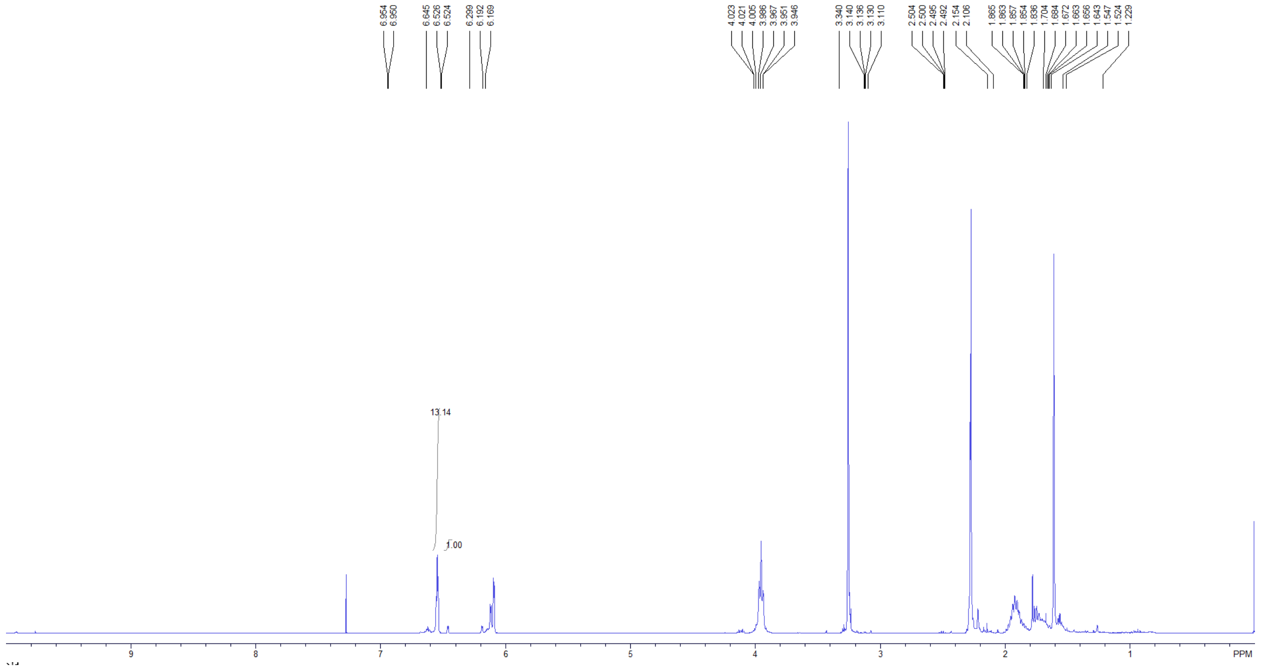

Initial Concentration = 31.4 mM

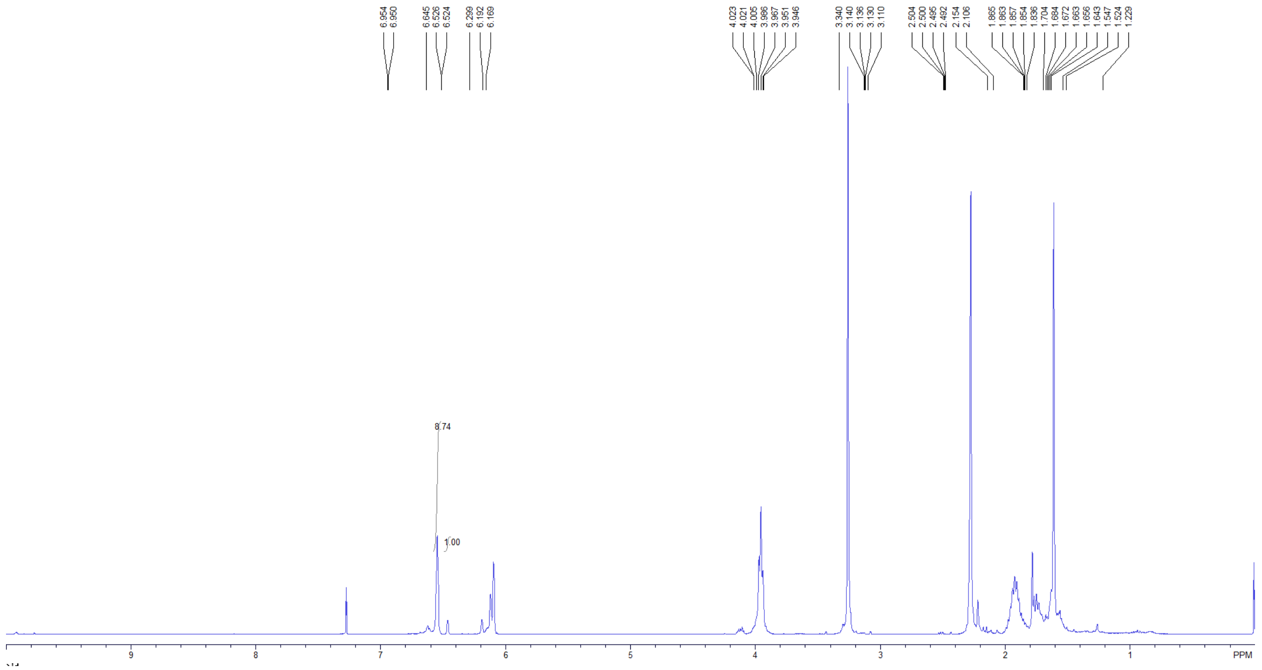

Initial Concentration = 15.7 mM

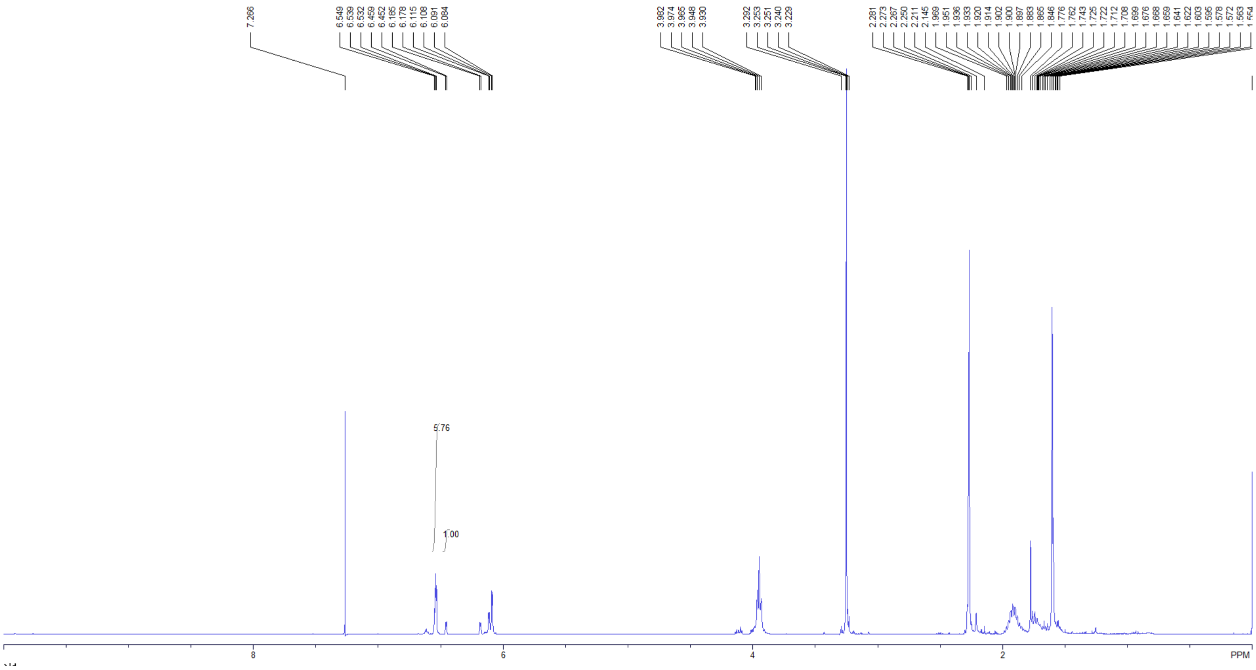

Initial Concentration = 7.9 mM

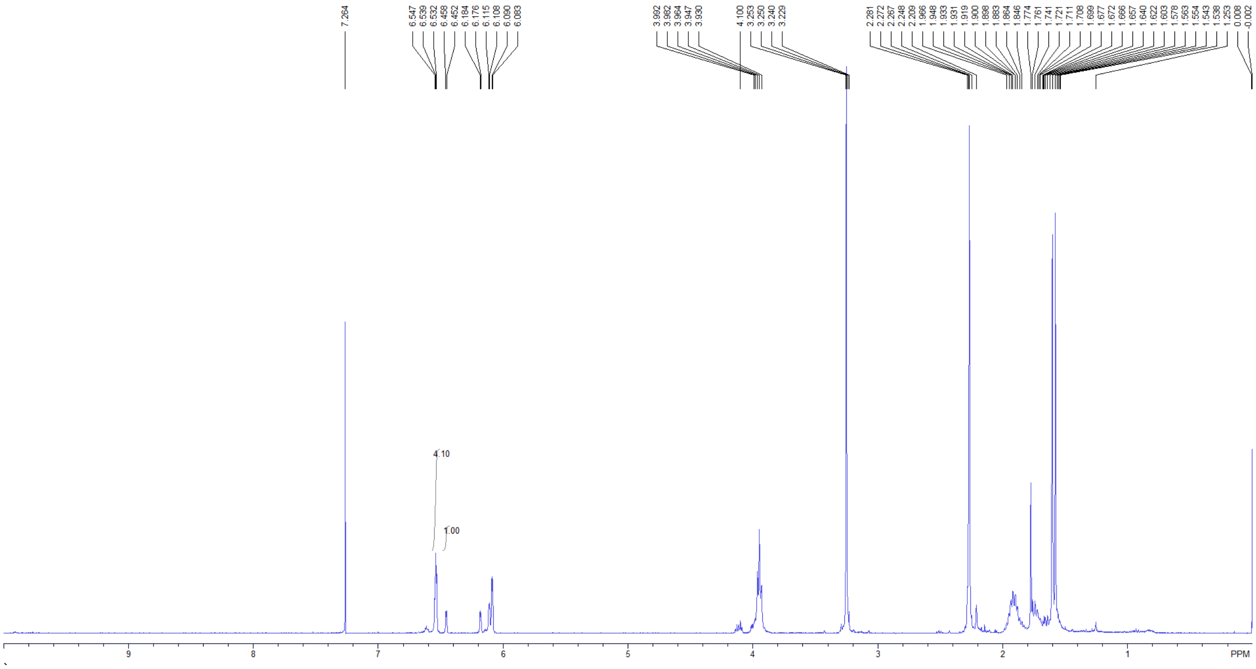

Initial Concentration = 3.9 mM

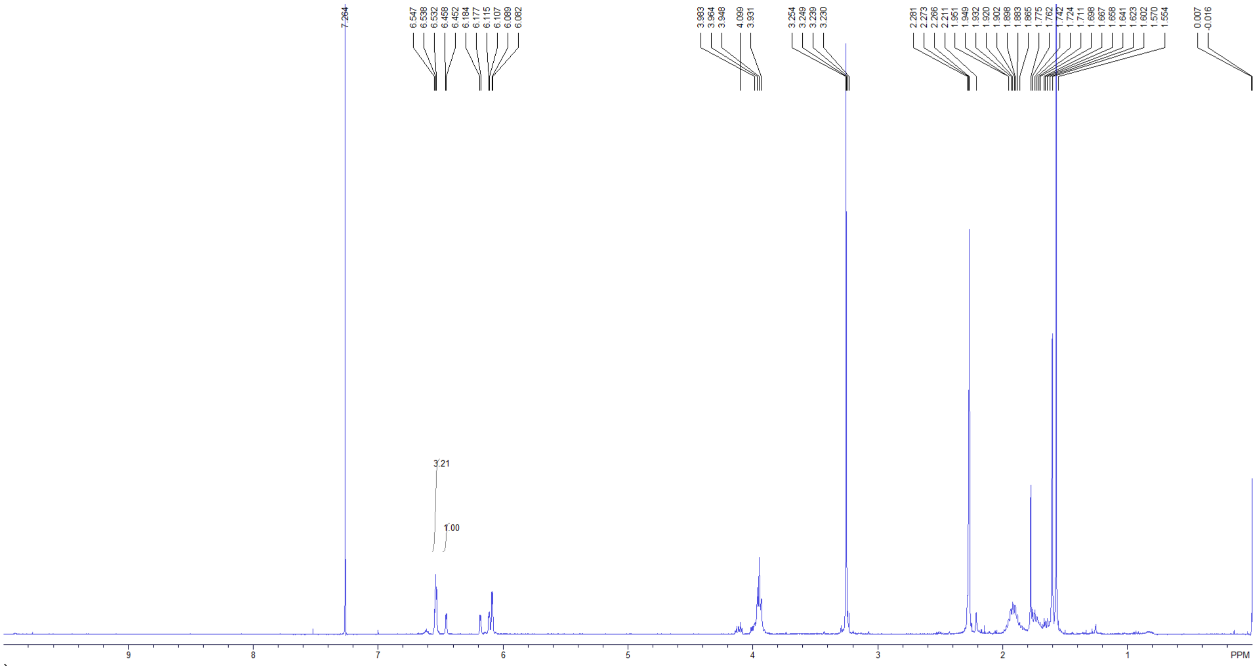

Initial Concentration = 2.0 mM

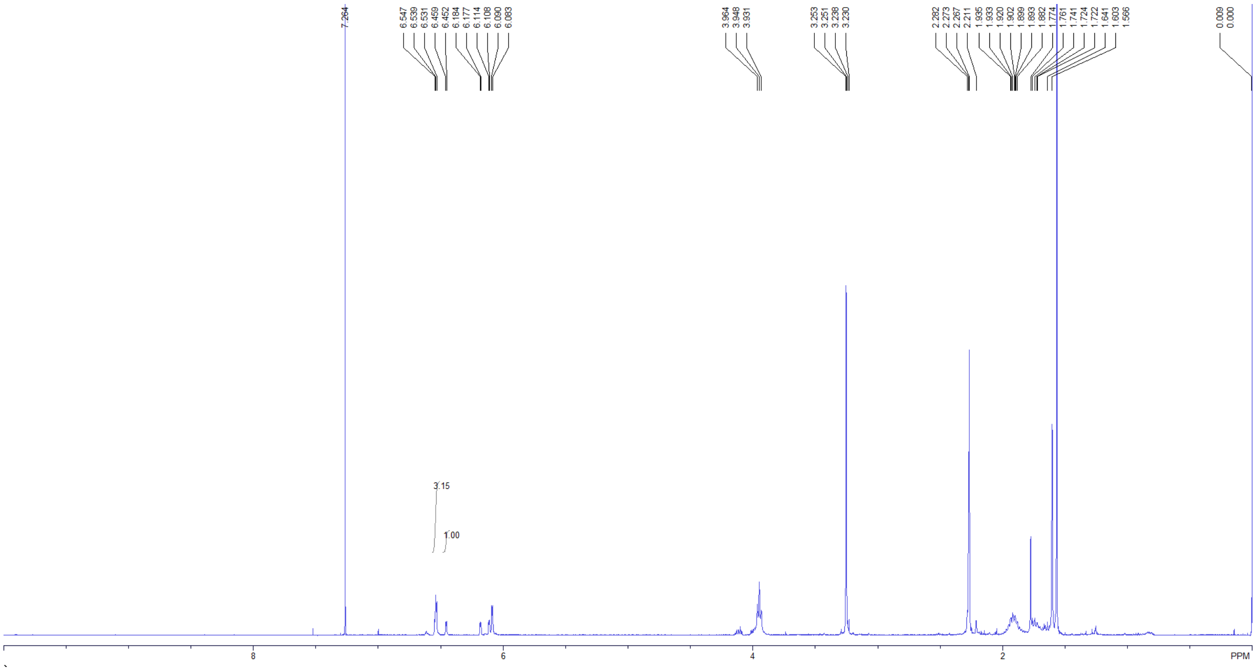

Initial Concentration = 1.0 mM

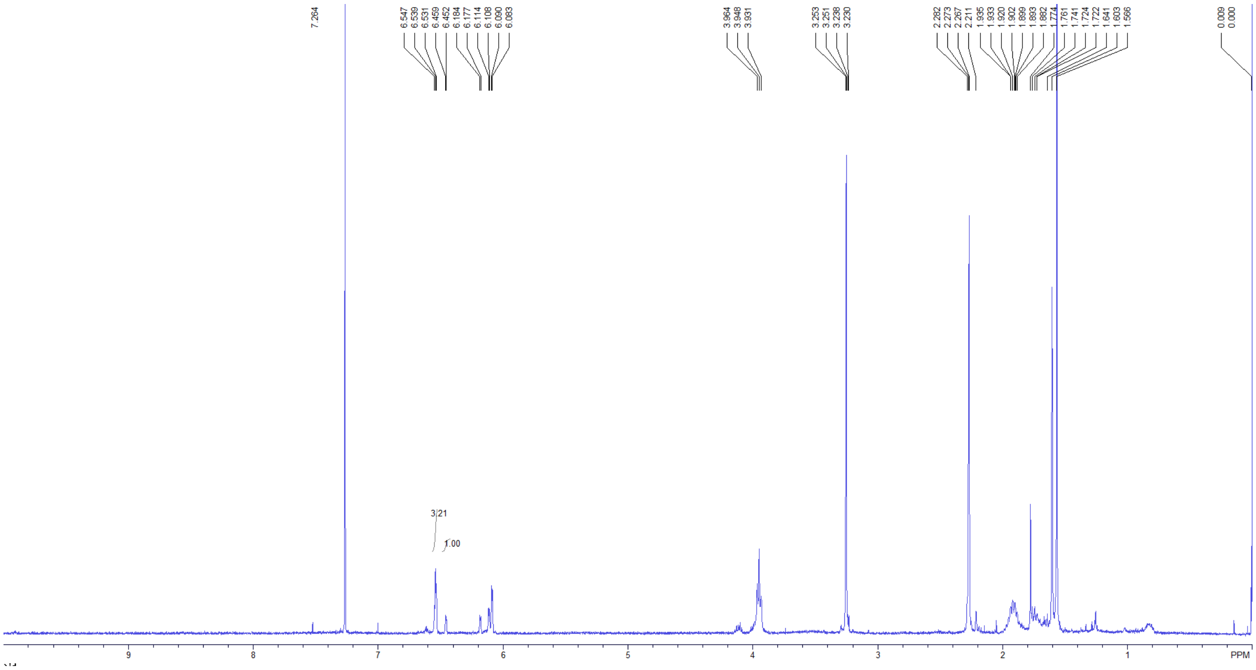

Initial Concentration = 0.5 mM

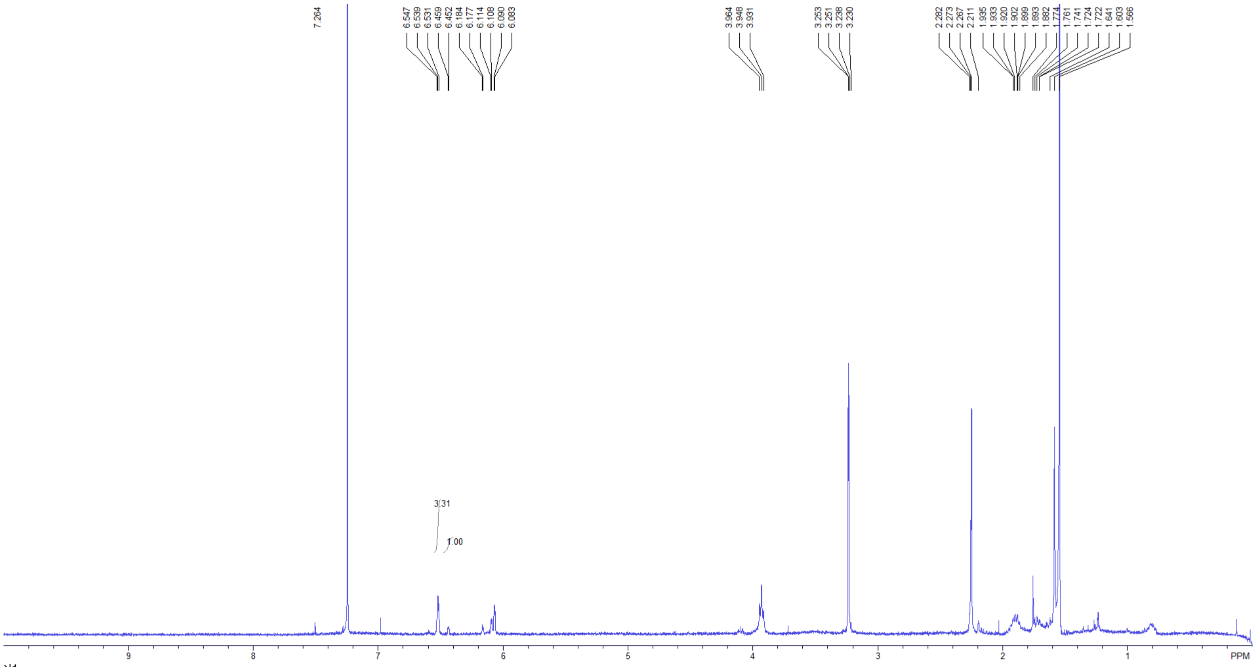

Computational Details

All calculations were carried out at the wB97X-D/6-31G* level of theory. A frequency calculation on the optimized structures confirmed the absence of any imaginary frequencies.

Monomer 4C_1

C2 symmetry

No. Imaginary Frequencies = 0

E = -1827.48311 H

Table S1. Coordinates for Monomer 4C_1

Center Number	Atomic Number	Atomic Type	Coordinates (Angstroms) X	Y	Z
1	8	0	-2.030862	0.245651	3.810502
2	8	0	0.961729	1.219146	-4.179652
3	8	0	-2.755895	2.877467	-2.059540
4	7	0	-1.376252	1.053330	-1.641828
5	7	0	-0.117435	0.674945	-2.186078
6	7	0	-0.929972	2.334714	-3.400092
7	6	0	-1.757956	1.264489	4.768659
8	1	0	-2.474950	1.079136	5.575429
9	1	0	-1.993475	2.244007	4.334228
10	6	0	0.072770	1.401887	-3.384354
11	6	0	-1.816139	2.167817	-2.331020
12	6	0	-1.092287	3.327001	-4.440599
13	1	0	-0.165476	3.891783	-4.559542
14	1	0	-1.900118	3.990896	-4.131445
15	1	0	-1.346184	2.844999	-5.387719
16	6	0	-1.539233	0.889032	-0.231164
17	6	0	-2.354369	-0.153411	0.224556
18	6	0	-2.479061	-0.338491	1.596221
19	1	0	-3.111187	-1.126477	1.993490
20	6	0	-1.013796	1.536317	2.019314
21	1	0	-0.476800	2.184332	2.701392
22	6	0	-0.862244	1.736273	0.651226
23	6	0	-3.125650	-1.014438	-0.741007
24	1	0	-4.034275	-0.495151	-1.066126
25	1	0	-3.423580	-1.955741	-0.270782
26	1	0	-2.547068	-1.234249	-1.641747
27	6	0	0.000000	2.859735	0.141513
28	1	0	0.718480	2.503405	-0.602557
29	1	0	0.563032	3.318915	0.957927
30	1	0	-0.616270	3.636510	-0.325940
31	6	0	-1.822469	0.504135	2.494535

32	8	0	2.030862	-0.245651	3.810502
33	8	0	2.755895	-2.877467	-2.059540
34	8	0	-0.961729	-1.219146	-4.179652
35	7	0	1.376252	-1.053330	-1.641828
36	7	0	0.117435	-0.674945	-2.186078
37	7	0	0.929972	-2.334714	-3.400092
38	6	0	0.341361	-1.251403	5.336937
39	1	0	-0.400133	-1.385358	4.544214
40	1	0	0.270640	-2.131787	5.989378
41	6	0	1.757956	-1.264489	4.768659
42	1	0	1.993475	-2.244007	4.334228
43	1	0	2.474950	-1.079136	5.575429
44	6	0	-0.072770	-1.401887	-3.384354
45	6	0	1.816139	-2.167817	-2.331020
46	6	0	1.092287	-3.327001	-4.440599
47	1	0	1.900118	-3.990896	-4.131445
48	1	0	0.165476	-3.891783	-4.559542
49	1	0	1.346184	-2.844999	-5.387719
50	6	0	1.539233	-0.889032	-0.231164
51	6	0	0.862244	-1.736273	0.651226
52	6	0	1.013796	-1.536317	2.019314
53	1	0	0.476800	-2.184332	2.701392
54	6	0	1.822469	-0.504135	2.494535
55	6	0	2.479061	0.338491	1.596221
56	1	0	3.111187	1.126477	1.993490
57	6	0	2.354369	0.153411	0.224556
58	6	0	0.000000	-2.859735	0.141513
59	1	0	0.616270	-3.636510	-0.325940
60	1	0	-0.563032	-3.318915	0.957927
61	1	0	-0.718480	-2.503405	-0.602557
62	6	0	3.125650	1.014438	-0.741007
63	1	0	2.547068	1.234249	-1.641747
64	1	0	3.423580	1.955741	-0.270782
65	1	0	4.034275	0.495151	-1.066126
66	6	0	-0.341361	1.251403	5.336937
67	1	0	-0.270640	2.131787	5.989378
68	1	0	0.400133	1.385358	4.544214
69	6	0	0.000000	0.000000	6.156403
70	1	0	0.846766	0.228333	6.817209
71	1	0	-0.846766	-0.228333	6.817209

Dimer 4C_2

Ci symmetry

No. Imaginary Frequencies = 0

E = -3654.98911 H

Table S2. Coordinates for Monomer 4C_2

Center Number	Atomic Number	Atomic Type	Coordinates (Angstroms) X	Y	Z
1	8	0	1.748885	-0.852190	3.613498
2	8	0	1.878597	-0.827045	-3.560039
3	8	0	-0.592362	1.475989	11.507436
4	8	0	3.510035	1.517050	9.446660
5	8	0	3.835437	1.579814	-9.318373
6	8	0	-0.196113	1.541482	-11.514800
7	7	0	1.527752	0.385923	9.008636
8	7	0	0.212202	0.541666	9.529102
9	7	0	1.597236	1.750371	10.756995
10	7	0	1.841659	0.443355	-8.951876
11	7	0	0.543874	0.600091	-9.514824
12	7	0	1.966841	1.816131	-10.690509
13	6	0	1.743448	0.128872	2.589427
14	1	0	2.587483	0.819735	2.725679
15	1	0	0.815808	0.717399	2.624723
16	6	0	1.855594	-0.633757	1.281862
17	1	0	1.031056	-1.357342	1.239081
18	1	0	2.780042	-1.222614	1.304352
19	6	0	1.830501	0.243166	0.030840
20	1	0	0.914117	0.848869	0.016755
21	1	0	2.671307	0.949172	0.048417
22	6	0	1.900429	-0.624677	-1.224790
23	1	0	2.825766	-1.212534	-1.218978
24	1	0	1.075733	-1.349295	-1.216082
25	6	0	1.833524	0.146954	-2.530220
26	1	0	0.906150	0.733431	-2.595009
27	1	0	2.680136	0.840740	-2.631044
28	6	0	0.308152	1.288576	10.726223
29	6	0	2.361685	1.239622	9.702335
30	6	0	2.123629	2.604404	11.799855
31	1	0	1.494448	3.490655	11.903711
32	1	0	3.132675	2.892254	11.503360
33	1	0	2.152296	2.067593	12.751054
34	6	0	1.638141	0.124384	7.608857
35	6	0	1.960607	-1.178060	7.208632

36	6	0	1.993411	-1.455652	5.848854
37	1	0	2.232366	-2.451982	5.490606
38	6	0	1.435352	0.840049	5.326136
39	1	0	1.216356	1.620352	4.606181
40	6	0	1.378393	1.140686	6.684598
41	6	0	2.304727	-2.238726	8.220689
42	1	0	3.322800	-2.085984	8.596060
43	1	0	2.254154	-3.234783	7.772338
44	1	0	1.640534	-2.206178	9.088018
45	6	0	0.678204	1.352148	-10.704776
46	6	0	2.696682	1.301729	-9.613327
47	6	0	2.526254	2.675950	-11.711184
48	1	0	2.591809	2.142477	-12.662447
49	1	0	3.522591	2.967662	-11.377737
50	1	0	1.896796	3.559445	-11.834829
51	6	0	1.905631	0.174027	-7.550710
52	6	0	1.614475	1.184804	-6.629820
53	6	0	1.624749	0.876009	-5.271997
54	1	0	1.381391	1.652133	-4.555370
55	6	0	1.899617	-0.424511	-4.855013
56	6	0	2.200533	-1.416591	-5.789116
57	1	0	2.426866	-2.415142	-5.428875
58	6	0	2.214204	-1.130892	-7.147496
59	6	0	1.310879	2.585430	-7.089465
60	1	0	2.208446	3.055532	-7.507634
61	1	0	0.958364	3.200381	-6.257779
62	1	0	0.535288	2.589893	-7.861439
63	6	0	2.592033	-2.185593	-8.153793
64	1	0	1.955486	-2.149497	-9.041543
65	1	0	2.529032	-3.184107	-7.712538
66	1	0	3.621175	-2.029253	-8.496025
67	6	0	1.057954	2.538269	7.142057
68	1	0	0.252897	2.537378	7.883325
69	1	0	0.738072	3.159248	6.301708
70	1	0	1.938736	3.005206	7.597728
71	6	0	1.724808	-0.457903	4.911017
72	8	0	-1.748885	0.852190	-3.613498
73	8	0	-1.878597	0.827045	3.560039
74	8	0	0.592362	-1.475989	-11.507436
75	8	0	-3.510035	-1.517050	-9.446660
76	8	0	-3.835437	-1.579814	9.318373
77	8	0	0.196113	-1.541482	11.514800
78	7	0	-1.527752	-0.385923	-9.008636
79	7	0	-0.212202	-0.541666	-9.529102
80	7	0	-1.597236	-1.750371	-10.756995
81	7	0	-1.841659	-0.443355	8.951876
82	7	0	-0.543874	-0.600091	9.514824
83	7	0	-1.966841	-1.816131	10.690509

84	6	0	-1.743448	-0.128872	-2.589427
85	1	0	-2.587483	-0.819735	-2.725679
86	1	0	-0.815808	-0.717399	-2.624723
87	6	0	-1.855594	0.633757	-1.281862
88	1	0	-1.031056	1.357342	-1.239081
89	1	0	-2.780042	1.222614	-1.304352
90	6	0	-1.830501	-0.243166	-0.030840
91	1	0	-0.914117	-0.848869	-0.016755
92	1	0	-2.671307	-0.949172	-0.048417
93	6	0	-1.900429	0.624677	1.224790
94	1	0	-2.825766	1.212534	1.218978
95	1	0	-1.075733	1.349295	1.216082
96	6	0	-1.833524	-0.146954	2.530220
97	1	0	-0.906150	-0.733431	2.595009
98	1	0	-2.680136	-0.840740	2.631044
99	6	0	-0.308152	-1.288576	-10.726223
100	6	0	-2.361685	-1.239622	-9.702335
101	6	0	-2.123629	-2.604404	-11.799855
102	1	0	-1.494448	-3.490655	-11.903711
103	1	0	-3.132675	-2.892254	-11.503360
104	1	0	-2.152296	-2.067593	-12.751054
105	6	0	-1.638141	-0.124384	-7.608857
106	6	0	-1.960607	1.178060	-7.208632
107	6	0	-1.993411	1.455652	-5.848854
108	1	0	-2.232366	2.451982	-5.490606
109	6	0	-1.435352	-0.840049	-5.326136
110	1	0	-1.216356	-1.620352	-4.606181
111	6	0	-1.378393	-1.140686	-6.684598
112	6	0	-2.304727	2.238726	-8.220689
113	1	0	-3.322800	2.085984	-8.596060
114	1	0	-2.254154	3.234783	-7.772338
115	1	0	-1.640534	2.206178	-9.088018
116	6	0	-0.678204	-1.352148	10.704776
117	6	0	-2.696682	-1.301729	9.613327
118	6	0	-2.526254	-2.675950	11.711184
119	1	0	-2.591809	-2.142477	12.662447
120	1	0	-3.522591	-2.967662	11.377737
121	1	0	-1.896796	-3.559445	11.834829
122	6	0	-1.905631	-0.174027	7.550710
123	6	0	-1.614475	-1.184804	6.629820
124	6	0	-1.624749	-0.876009	5.271997
125	1	0	-1.381391	-1.652133	4.555370
126	6	0	-1.899617	0.424511	4.855013
127	6	0	-2.200533	1.416591	5.789116
128	1	0	-2.426866	2.415142	5.428875
129	6	0	-2.214204	1.130892	7.147496
130	6	0	-1.310879	-2.585430	7.089465
131	1	0	-2.208446	-3.055532	7.507634

132	1	0	-0.958364	-3.200381	6.257779
133	1	0	-0.535288	-2.589893	7.861439
134	6	0	-2.592033	2.185593	8.153793
135	1	0	-1.955486	2.149497	9.041543
136	1	0	-2.529032	3.184107	7.712538
137	1	0	-3.621175	2.029253	8.496025
138	6	0	-1.057954	-2.538269	-7.142057
139	1	0	-0.252897	-2.537378	-7.883325
140	1	0	-0.738072	-3.159248	-6.301708
141	1	0	-1.938736	-3.005206	-7.597728
142	6	0	-1.724808	0.457903	-4.911017

General Information for Crystal Structures

The diffraction data were collected on a Rigaku XtaLAB Synergy-S Dualflex HyPix diffractometer with monochromated Cu-K_α radiation. The structure was solved by direct methods (OLEX2.solve)[1,2] and refined by full-matrix least-squares on F^2 values (SHELXL).[3] All the heavy atoms were refined anisotropically. The hydrogen atoms were localized from the difference electron density maps, after which they were refined isotropically (U_{iso} with a factor of 1.2 for CH and CH_2 groups, 1.5 for CH_3 groups) with riding coordinates or as rotation CH_3groups. Mercury was used for the structure presentation in the Figures.[4]

Crystal Structure Data for Dimer $4a_2$

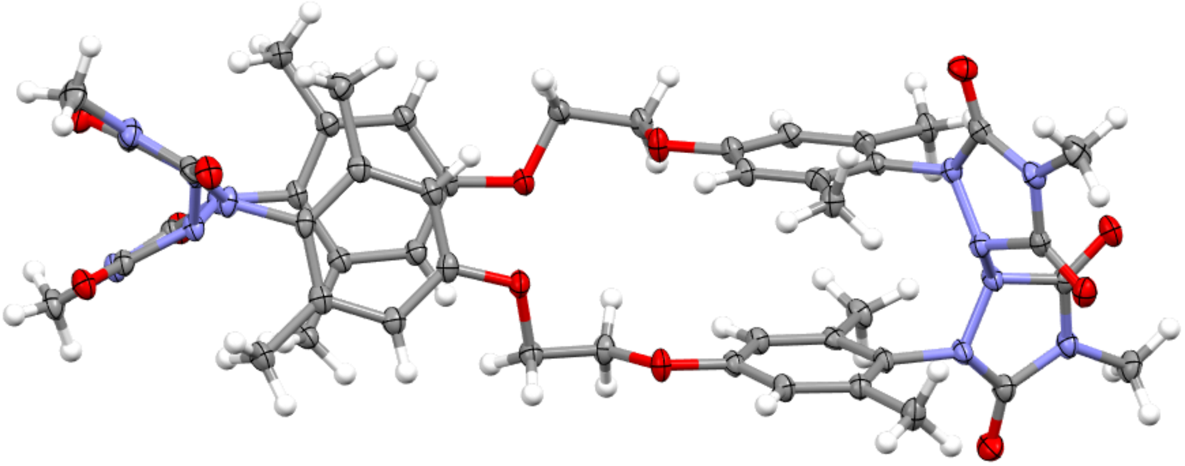

*elipsoids represent 50% probability level

Table S3. Crystal data and structure refinement for $4a_2$.

Identification code	GB2020_Dec
Empirical formula	$C_{49}H_{54}Cl_2N_{12}O_{12}$
Formula weight	1073.94
Temperature/K	100.15
Crystal system	monoclinic
Space group	$P2_1$
a/Å	8.83819(7)
b/Å	14.23369(11)
c/Å	20.10392(16)
α/°	90
β/°	91.7994(7)
γ/°	90
Volume/Å^3	2527.83(3)
Z	2
ρ_{calc}g/cm^3	1.411
μ/mm^{-1}	1.792
F(000)	1124.0
Crystal size/mm^3	0.33 × 0.13 × 0.1
Radiation	CuKα (λ = 1.54184)

2Θ range for data collection/°	4.398 to 155.172
Index ranges	$-11 \leq h \leq 10$, $-18 \leq k \leq 18$, $-24 \leq l \leq 24$
Reflections collected	35964
Independent reflections	10128 [R_{int} = 0.0441, R_{sigma} = 0.0387]
Data/restraints/parameters	10128/1/689
Goodness-of-fit on F^2	1.035
Final R indexes [I>=2σ (I)]	R_1 = 0.0338, wR_2 = 0.0885
Final R indexes [all data]	R_1 = 0.0353, wR_2 = 0.0896
Largest diff. peak/hole / e Å^{-3}	0.50/-0.37
Flack parameter	0.491(13)

Table S4. Fractional Atomic Coordinates (×10^4) and Equivalent Isotropic Displacement Parameters (Å^2×10^3) for 4a$_2$. U_{eq} is defined as 1/3 of the trace of the orthogonalised U_{IJ} tensor.

Atom	*x*	*y*	*z*	U(eq)
Cl1	4557.6(9)	12871.1(7)	-1928.1(4)	44.87(19)
Cl2	6423.8(10)	11623.6(6)	-2730.1(4)	42.27(18)
C49	6395(4)	12446(2)	-2067.1(16)	36.4(7)
O1	5563(2)	9820.9(14)	593.9(10)	29.0(4)
O2	942(2)	8333.5(13)	525.1(9)	25.5(4)
O3	2924(2)	6610.6(14)	578.6(9)	25.9(4)
O4	229(2)	6182.6(13)	-1380.1(10)	26.5(4)
O5	3703(2)	8955.4(13)	-3344.9(9)	25.4(4)
O6	5315.1(19)	10178.1(14)	-4206.5(9)	23.1(4)
O7	9067(2)	11735.4(13)	-6629.5(9)	24.8(4)
O8	11956(2)	9185.9(13)	-7148.6(9)	25.3(4)
O9	9014(2)	8881.3(14)	-7923.7(10)	30.6(4)
O10	9726(2)	6155.5(13)	-6730.2(10)	27.9(4)
O11	7931(2)	8140.1(13)	-3802.5(9)	24.1(4)
O12	7901(2)	8374.7(13)	-2337.3(9)	23.7(4)
N1	4537(2)	8630.7(15)	-70.0(10)	20.9(4)
N2	3127(2)	9234.6(16)	715.3(11)	22.7(4)
N3	2991(2)	8329.0(15)	-172.0(10)	19.5(4)
N4	2820(2)	7433.2(14)	-425.9(10)	19.1(4)
N5	1467(2)	6135.3(15)	-345.4(11)	22.5(4)
N6	1774(2)	7390.7(15)	-985.9(10)	19.1(4)
N7	8730(2)	10135.5(15)	-6483.9(10)	20.3(4)
N8	10827(2)	10637.0(15)	-6963.0(10)	20.8(4)
N9	9746(2)	9356.5(14)	-6558.4(10)	19.2(4)

Table S4. Fractional Atomic Coordinates (×10⁴) and Equivalent Isotropic Displacement Parameters (Å²×10³) for 4a₂. U_{eq} is defined as 1/3 of the trace of the orthogonalised U_{IJ} tensor.

Atom	*x*	*y*	*z*	U(eq)
N10	9029(2)	8543.6(15)	-6789.1(10)	21.2(4)
N11	9364(3)	7369.7(16)	-7496.8(11)	25.2(5)
N12	9512(2)	7721.1(15)	-6425.9(10)	21.5(4)
C1	4547(3)	9290.3(18)	430.7(13)	22.2(5)
C2	2716(3)	9761(2)	1306.7(13)	27.2(5)
C3	2198(3)	8604.3(18)	391.3(12)	20.6(5)
C4	2439(3)	6697.0(18)	12.7(12)	20.6(5)
C5	946(3)	5215(2)	-132.7(16)	31.6(6)
C6	1057(3)	6528.1(18)	-960.0(13)	21.3(5)
C7	2254(3)	7803.4(18)	-1597.2(12)	18.9(4)
C8	3301(3)	7320.6(18)	-1985.2(13)	20.5(5)
C9	3896(3)	6371.8(17)	-1783.0(13)	24.0(5)
C10	3755(3)	7744.1(18)	-2564.6(12)	20.8(5)
C11	3182(3)	8618.9(18)	-2756.5(12)	21.1(5)
C12	2156(3)	9090.8(18)	-2368.1(13)	20.8(5)
C13	1685(3)	8680.2(17)	-1773.2(12)	19.3(5)
C14	599(3)	9209.8(18)	-1347.7(13)	22.4(5)
C15	3165(3)	9848.4(18)	-3564.0(13)	23.3(5)
C16	3723(3)	9998.3(19)	-4252.0(13)	22.7(5)
C17	6067(3)	10181.8(18)	-4791.6(13)	21.1(5)
C18	5396(3)	10058.5(18)	-5413.6(13)	21.8(5)
C19	6278(3)	10044.2(18)	-5981.9(13)	21.2(5)
C20	5534(3)	9947(2)	-6662.6(14)	27.8(6)
C21	7839(3)	10151.5(17)	-5896.8(12)	20.2(5)
C22	8536(3)	10302.2(16)	-5270.5(12)	19.9(5)
C23	10221(3)	10440.9(18)	-5180.7(13)	22.0(5)
C24	7632(3)	10322.1(17)	-4718.6(12)	19.9(5)
C25	9477(3)	10936.6(17)	-6686.1(12)	19.6(5)
C26	11887(3)	11276.2(19)	-7265.0(13)	25.3(5)
C27	10983(3)	9684.7(18)	-6928.9(12)	19.9(5)
C28	9131(3)	8321.3(19)	-7475.3(13)	23.2(5)
C29	9457(4)	6839(2)	-8113.2(14)	32.6(6)
C30	9551(3)	6984.5(18)	-6862.0(14)	23.3(5)
C31	9134(3)	7701.0(17)	-5735.9(12)	20.5(5)
C32	7612(3)	7625.4(17)	-5566.0(13)	21.7(5)
C33	6372(3)	7464.8(19)	-6082.1(14)	26.4(5)
C34	7276(3)	7746.9(18)	-4903.5(13)	22.2(5)
C35	8410(3)	7964.0(18)	-4432.2(13)	22.1(5)

Table S4. Fractional Atomic Coordinates (×10^4) and Equivalent Isotropic Displacement Parameters (Å^2×10^3) for 4a$_2$. U_{eq} is defined as 1/3 of the trace of the orthogonalised U_{IJ} tensor.

Atom	*x*	*y*	*z*	U(eq)
C36	9926(3)	7987.1(18)	-4605.0(13)	22.1(5)
C37	10299(3)	7856.2(18)	-5269.2(13)	22.2(5)
C38	11936(3)	7882(2)	-5464.3(14)	25.3(5)
C39	8888(3)	8721.1(18)	-3385.4(12)	22.7(5)
C40	7935(3)	9091.8(18)	-2835.3(13)	22.9(5)
C41	7088(3)	8524.6(18)	-1781.3(12)	20.1(5)
C42	6161(3)	9298.5(18)	-1687.4(12)	20.2(5)
C43	5324(3)	9359.2(17)	-1110.0(12)	19.2(5)
C44	4325(3)	10196.4(18)	-1013.0(14)	24.2(5)
C45	5473(3)	8645.0(18)	-637.9(12)	19.4(5)
C46	6465(3)	7889.3(18)	-712.8(12)	21.1(5)
C47	6684(3)	7146(2)	-183.6(14)	27.7(6)
C48	7268(3)	7834.9(18)	-1293.4(12)	22.0(5)

Table S5. Anisotropic Displacement Parameters (Å^2×10^3) for 4a$_2$. The Anisotropic displacement factor exponent takes the form: $-2\pi^2[h^2a^{*2}U_{11}+2hka^*b^*U_{12}+\ldots]$.

Atom	U_{11}	U_{22}	U_{33}	U_{23}	U_{13}	U_{12}
Cl1	37.9(4)	51.7(5)	45.1(4)	-1.7(4)	3.1(3)	2.4(3)
Cl2	50.5(4)	36.7(4)	39.4(4)	0.5(3)	-0.3(3)	5.3(3)
C49	37.3(16)	38.8(17)	33.1(16)	3.6(12)	0.0(12)	-3.1(12)
O1	29.1(10)	31.6(10)	26.6(10)	-10.8(8)	4.7(7)	-4.9(8)
O2	23.1(9)	25.5(9)	28.6(9)	0.2(7)	10.4(7)	1.2(7)
O3	29.1(9)	27.0(9)	21.9(9)	3.7(7)	4.1(7)	4.2(8)
O4	24.7(9)	23.7(9)	31.0(10)	0.2(8)	-1.0(7)	-5.0(7)
O5	29.9(9)	23.7(9)	23.2(9)	6.2(7)	11.9(7)	6.6(7)
O6	19.8(8)	29.9(9)	19.9(9)	0.9(7)	5.4(6)	0.4(7)
O7	28.9(9)	18.7(9)	27.1(9)	1.0(7)	5.4(7)	2.3(7)
O8	25.1(9)	23.2(9)	28.1(10)	-2.3(7)	9.2(7)	0.3(7)
O9	42.5(11)	28.1(10)	21.5(9)	1.7(8)	4.0(8)	-4.7(8)
O10	33.6(10)	19.2(9)	31.3(10)	-2.4(7)	8.5(8)	-2.7(7)
O11	25.2(9)	29.3(9)	18.3(9)	-1.9(7)	6.8(7)	-3.9(7)
O12	28.8(9)	21.7(9)	21.3(9)	1.7(7)	11.2(7)	4.1(7)
N1	18.8(10)	23.6(10)	20.7(10)	-4.4(8)	6.4(8)	-3.6(8)
N2	24.0(10)	23.4(10)	21.1(11)	-4.3(8)	6.8(8)	1.7(9)
N3	19.6(10)	18.1(10)	21.1(10)	-3.0(8)	6.7(8)	-2.6(8)
N4	21.5(10)	17.3(9)	18.6(10)	-1.0(8)	3.1(8)	-1.1(8)

Table S5. Anisotropic Displacement Parameters (Å^2×10^3) for 4a$_2$. The Anisotropic displacement factor exponent takes the form: $-2\pi^2[h^2a^{*2}U_{11}+2hka^*b^*U_{12}+\ldots]$.

Atom	U_{11}	U_{22}	U_{33}	U_{23}	U_{13}	U_{12}
N5	22.5(10)	18.9(10)	26.2(11)	3.4(8)	4.1(8)	-2.5(8)
N6	19.6(10)	18.2(9)	19.6(10)	1.8(8)	3.1(8)	-1.8(8)
N7	22.5(10)	18.0(10)	20.7(10)	0.1(8)	6.5(8)	1.5(8)
N8	22.4(10)	18.4(10)	22.0(10)	0.7(8)	7.1(8)	-1.2(8)
N9	22.1(10)	14.5(9)	21.5(10)	-2.3(8)	7.2(8)	-1.5(8)
N10	27.1(11)	17.2(10)	19.6(10)	-0.3(8)	4.6(8)	-3.0(8)
N11	31.0(12)	23.8(10)	21.2(11)	-3.3(9)	6.3(9)	-4.9(9)
N12	30.3(11)	15.8(10)	18.8(10)	0.5(8)	6.7(8)	-1.1(8)
C1	23.6(12)	22.0(12)	21.2(12)	-2.3(10)	4.2(9)	1.4(10)
C2	32.5(14)	27.7(13)	21.6(13)	-7.1(10)	7.5(10)	2.4(11)
C3	22.2(12)	19.6(11)	20.3(11)	-0.3(9)	5.1(9)	4.6(9)
C4	19.4(11)	21.0(11)	21.7(12)	1.6(9)	7.1(9)	2.7(9)
C5	32.6(14)	23.0(13)	39.4(17)	10.2(11)	5.6(12)	-6.3(11)
C6	19.3(11)	19.0(11)	25.8(12)	0.1(10)	5.8(9)	0.5(9)
C7	18.9(11)	20.6(11)	17.3(11)	-0.4(9)	3.1(8)	-3.2(9)
C8	19.9(11)	18.6(11)	23.0(12)	-2.4(9)	0.5(9)	-0.7(9)
C9	28.8(13)	19.0(12)	24.7(13)	1.5(9)	7.0(10)	3.8(10)
C10	21.4(11)	20.1(11)	21.2(12)	-1.1(9)	6.5(9)	1.7(9)
C11	20.7(11)	21.8(12)	21.2(12)	1.7(9)	4.5(9)	-0.9(9)
C12	19.9(11)	19.5(11)	23.0(12)	1.9(9)	1.7(9)	1.5(9)
C13	17.0(11)	19.9(11)	21.1(11)	-0.9(9)	3.4(9)	-0.6(9)
C14	22.6(12)	21.4(12)	23.4(12)	1.6(9)	6.4(9)	2.2(10)
C15	21.7(12)	22.4(12)	26.1(13)	4.2(10)	8.1(10)	3.2(9)
C16	19.1(11)	26.6(13)	22.6(12)	3.1(10)	4.9(9)	0.9(9)
C17	23.3(12)	18.6(11)	21.7(12)	2.6(9)	7.5(9)	1.3(9)
C18	19.2(11)	22.9(12)	23.6(12)	3.5(9)	4.6(9)	0.6(9)
C19	21.0(12)	21.0(11)	21.7(12)	1.4(9)	2.5(9)	-0.7(9)
C20	23.5(12)	37.3(15)	22.7(13)	1.5(11)	2.2(10)	-2.2(11)
C21	20.8(11)	18.4(11)	21.7(12)	0.9(9)	7.6(9)	0.2(9)
C22	20.7(11)	15.5(11)	23.6(12)	-1.3(9)	4.7(9)	-1.0(9)
C23	19.5(12)	22.3(12)	24.5(13)	-2.0(10)	3.8(9)	-1.1(9)
C24	20.7(11)	18.5(11)	20.5(12)	-1.0(9)	2.5(9)	-0.1(9)
C25	19.4(11)	20.7(12)	18.8(11)	0.5(9)	2.4(9)	-1.1(9)
C26	28.3(13)	22.5(12)	25.6(13)	0.4(10)	9.4(10)	-6.9(10)
C27	22.0(12)	20.0(11)	18.2(11)	0.1(9)	5.2(9)	-3.1(9)
C28	26.7(12)	24.4(12)	18.8(12)	-1.4(10)	5.4(9)	-4.3(10)
C29	44.1(16)	29.9(15)	24.2(14)	-9.2(11)	6.0(12)	-4.9(12)
C30	23.1(12)	20.1(12)	26.8(13)	-2.8(10)	5.8(10)	-5.0(9)
C31	27.3(12)	15.5(11)	19.3(12)	-0.1(9)	7.2(9)	-1.7(9)

Table S5. Anisotropic Displacement Parameters (Å²×10³) for 4a₂. The Anisotropic displacement factor exponent takes the form: $-2\pi^2[h^2a^{*2}U_{11}+2hka^*b^*U_{12}+\ldots]$.

Atom	U_{11}	U_{22}	U_{33}	U_{23}	U_{13}	U_{12}
C32	25.7(12)	16.2(11)	23.4(12)	1.1(9)	4.1(9)	-2.8(9)
C33	28.1(13)	26.2(13)	25.0(13)	0.6(10)	3.5(10)	-4.8(10)
C34	21.6(11)	20.6(11)	24.7(12)	1.9(10)	6.9(9)	-1.7(9)
C35	27.3(12)	19.0(11)	20.4(12)	2.2(9)	6.3(9)	1.9(9)
C36	24.3(12)	19.2(11)	23.0(13)	0.8(9)	3.9(9)	-0.2(9)
C37	26.5(12)	15.4(11)	25.1(12)	0.6(9)	6.8(9)	-0.4(9)
C38	24.1(12)	23.7(12)	28.6(13)	-0.6(10)	7.5(10)	0.2(10)
C39	24.1(12)	22.5(12)	21.7(12)	-0.9(10)	4.8(9)	-0.7(10)
C40	28.1(13)	19.6(12)	21.2(12)	2.2(9)	5.0(10)	0.4(9)
C41	20.1(11)	21.0(11)	19.5(11)	-4.0(9)	5.9(9)	-1.2(9)
C42	21.0(11)	17.6(11)	22.2(12)	-0.6(9)	3.9(9)	0.1(9)
C43	17.7(11)	17.4(11)	22.5(12)	-3.5(9)	2.2(9)	-2.3(9)
C44	22.7(12)	19.8(11)	30.6(14)	-3.0(10)	6.6(10)	1.9(10)
C45	18.2(11)	21.5(11)	19.0(11)	-3.9(9)	6.5(9)	-1.9(9)
C46	22.6(11)	21.3(12)	19.6(12)	-0.9(9)	3.7(9)	-1.8(10)
C47	31.6(14)	28.8(14)	23.1(13)	4.2(10)	7.2(10)	7.7(11)
C48	22.6(11)	21.2(12)	22.4(12)	-1.2(10)	4.9(9)	1.5(10)

Table S6. Bond Lengths for 4a₂.

Atom	Atom	Length/Å	Atom	Atom	Length/Å
Cl1	C49	1.763(3)	N10	N12	1.438(3)
Cl2	C49	1.775(3)	N10	C28	1.421(3)
O1	C1	1.210(3)	N11	C28	1.371(3)
O2	C3	1.214(3)	N11	C29	1.456(3)
O3	C4	1.210(3)	N11	C30	1.394(4)
O4	C6	1.205(3)	N12	C30	1.368(3)
O5	C11	1.369(3)	N12	C31	1.437(3)
O5	C15	1.422(3)	C7	C8	1.407(3)
O6	C16	1.430(3)	C7	C13	1.387(4)
O6	C17	1.369(3)	C8	C9	1.501(3)
O7	C25	1.200(3)	C8	C10	1.383(4)
O8	C27	1.210(3)	C10	C11	1.394(4)
O9	C28	1.205(3)	C11	C12	1.388(4)
O10	C30	1.218(3)	C12	C13	1.406(3)
O11	C35	1.371(3)	C13	C14	1.508(3)
O11	C39	1.435(3)	C15	C16	1.498(4)
O12	C40	1.431(3)	C17	C18	1.378(4)

Table S6. Bond Lengths for 4a$_2$.

Atom	Atom	Length/Å	Atom	Atom	Length/Å
O12	C41	1.364(3)	C17	C24	1.401(4)
N1	N3	1.440(3)	C18	C19	1.404(3)
N1	C1	1.376(3)	C19	C20	1.506(4)
N1	C45	1.431(3)	C19	C21	1.393(3)
N2	C1	1.398(3)	C21	C22	1.401(4)
N2	C2	1.461(3)	C22	C23	1.508(3)
N2	C3	1.367(3)	C22	C24	1.387(3)
N3	N4	1.380(3)	C31	C32	1.402(4)
N3	C3	1.406(3)	C31	C37	1.388(4)
N4	N6	1.435(3)	C32	C33	1.503(4)
N4	C4	1.417(3)	C32	C34	1.385(4)
N5	C4	1.362(3)	C34	C35	1.392(4)
N5	C5	1.456(3)	C35	C36	1.395(4)
N5	C6	1.394(3)	C36	C37	1.398(4)
N6	C6	1.384(3)	C37	C38	1.511(3)
N6	C7	1.438(3)	C39	C40	1.506(3)
N7	N9	1.438(3)	C41	C42	1.389(3)
N7	C21	1.440(3)	C41	C48	1.393(4)
N7	C25	1.385(3)	C42	C43	1.398(3)
N8	C25	1.399(3)	C43	C44	1.499(3)
N8	C26	1.453(3)	C43	C45	1.394(4)
N8	C27	1.364(3)	C45	C46	1.399(4)
N9	N10	1.392(3)	C46	C47	1.509(4)
N9	C27	1.421(3)	C46	C48	1.387(3)

Table S7. Bond Angles for 4a$_2$.

Atom	Atom	Atom	Angle/°	Atom	Atom	Atom	Angle/°
Cl1	C49	Cl2	112.37(18)	C11	C12	C13	119.6(2)
C11	O5	C15	117.51(19)	C7	C13	C12	118.4(2)
C17	O6	C16	116.65(19)	C7	C13	C14	122.5(2)
C35	O11	C39	116.84(19)	C12	C13	C14	119.1(2)
C41	O12	C40	119.01(19)	O5	C15	C16	107.3(2)
C1	N1	N3	106.99(19)	O6	C16	C15	108.5(2)
C1	N1	C45	125.7(2)	O6	C17	C18	124.9(2)
C45	N1	N3	117.40(19)	O6	C17	C24	114.5(2)
C1	N2	C2	123.6(2)	C18	C17	C24	120.6(2)
C3	N2	C1	112.1(2)	C17	C18	C19	120.4(2)
C3	N2	C2	124.3(2)	C18	C19	C20	120.2(2)

Table S7. Bond Angles for $4a_2$.

Atom	Atom	Atom	Angle/°	Atom	Atom	Atom	Angle/°
N4	N3	N1	114.90(19)	C21	C19	C18	118.1(2)
N4	N3	C3	120.2(2)	C21	C19	C20	121.6(2)
C3	N3	N1	107.26(19)	C19	C21	N7	117.6(2)
N3	N4	N6	112.94(18)	C19	C21	C22	122.2(2)
N3	N4	C4	118.6(2)	C22	C21	N7	120.2(2)
C4	N4	N6	107.30(19)	C21	C22	C23	122.2(2)
C4	N5	C5	124.9(2)	C24	C22	C21	118.3(2)
C4	N5	C6	112.0(2)	C24	C22	C23	119.5(2)
C6	N5	C5	123.0(2)	C22	C24	C17	120.3(2)
N4	N6	C7	116.95(18)	O7	C25	N7	127.2(2)
C6	N6	N4	107.01(19)	O7	C25	N8	126.2(2)
C6	N6	C7	122.8(2)	N7	C25	N8	106.6(2)
N9	N7	C21	117.21(19)	O8	C27	N8	129.6(2)
C25	N7	N9	107.42(18)	O8	C27	N9	124.5(2)
C25	N7	C21	120.4(2)	N8	C27	N9	105.9(2)
C25	N8	C26	123.0(2)	O9	C28	N10	124.9(2)
C27	N8	C25	111.7(2)	O9	C28	N11	129.7(2)
C27	N8	C26	125.3(2)	N11	C28	N10	105.4(2)
N10	N9	N7	113.35(19)	O10	C30	N11	126.2(2)
N10	N9	C27	116.65(19)	O10	C30	N12	127.5(3)
C27	N9	N7	107.12(19)	N12	C30	N11	106.3(2)
N9	N10	N12	112.49(19)	C32	C31	N12	119.4(2)
N9	N10	C28	117.8(2)	C37	C31	N12	117.3(2)
C28	N10	N12	106.55(19)	C37	C31	C32	122.9(2)
C28	N11	C29	123.5(2)	C31	C32	C33	121.9(2)
C28	N11	C30	111.9(2)	C34	C32	C31	117.5(2)
C30	N11	C29	124.5(2)	C34	C32	C33	120.6(2)
C30	N12	N10	108.1(2)	C32	C34	C35	120.7(2)
C30	N12	C31	127.9(2)	O11	C35	C34	115.6(2)
C31	N12	N10	115.60(19)	O11	C35	C36	123.6(2)
O1	C1	N1	127.7(2)	C34	C35	C36	120.8(2)
O1	C1	N2	126.3(2)	C35	C36	C37	119.5(2)
N1	C1	N2	106.0(2)	C31	C37	C36	118.4(2)
O2	C3	N2	130.0(2)	C31	C37	C38	121.6(2)
O2	C3	N3	124.9(2)	C36	C37	C38	120.1(2)
N2	C3	N3	105.0(2)	O11	C39	C40	107.3(2)
O3	C4	N4	125.2(2)	O12	C40	C39	106.8(2)
O3	C4	N5	129.5(2)	O12	C41	C42	124.3(2)
N5	C4	N4	105.3(2)	O12	C41	C48	114.5(2)
O4	C6	N5	126.4(2)	C42	C41	C48	121.2(2)

Table S7. Bond Angles for $4a_2$.

Atom	Atom	Atom	Angle/°
O4	C6	N6	127.2(2)
N6	C6	N5	106.4(2)
C8	C7	N6	119.4(2)
C13	C7	N6	118.1(2)
C13	C7	C8	122.5(2)
C7	C8	C9	121.3(2)
C10	C8	C7	117.9(2)
C10	C8	C9	120.8(2)
C8	C10	C11	120.7(2)
O5	C11	C10	115.0(2)
O5	C11	C12	124.1(2)
C12	C11	C10	121.0(2)
C41	C42	C43	119.4(2)
C42	C43	C44	119.1(2)
C45	C43	C42	118.6(2)
C45	C43	C44	122.3(2)
C43	C45	N1	120.6(2)
C43	C45	C46	122.2(2)
C46	C45	N1	117.1(2)
C45	C46	C47	122.0(2)
C48	C46	C45	118.2(2)
C48	C46	C47	119.8(2)
C46	C48	C41	120.2(2)

Table S8. Torsion Angles for $4a_2$.

A	B	C	D	Angle/°
O5	C11	C12	C13	179.6(2)
O5	C15	C16	O6	-72.5(3)
O6	C17	C18	C19	-177.9(2)
O6	C17	C24	C22	177.3(2)
O11	C35	C36	C37	-176.0(2)
O11	C39	C40	O12	82.9(2)
O12	C41	C42	C43	177.1(2)
O12	C41	C48	C46	-177.9(2)
N1	N3	N4	N6	-130.7(2)
N1	N3	N4	C4	102.6(2)
N1	N3	C3	O2	-167.2(2)
N1	N3	C3	N2	14.5(3)
N1	C45	C46	C47	-7.0(4)
N1	C45	C46	C48	173.3(2)
N3	N1	C1	O1	-167.2(3)
N3	N1	C1	N2	12.2(3)
N3	N1	C45	C43	76.2(3)
N3	N1	C45	C46	-100.3(3)
N3	N4	N6	C6	-146.8(2)
N3	N4	N6	C7	70.8(3)
N3	N4	C4	O3	-38.5(3)
N3	N4	C4	N5	142.5(2)
N4	N3	C3	O2	-33.5(4)
C8	C10	C11	O5	-179.0(2)
C8	C10	C11	C12	0.7(4)
C9	C8	C10	C11	179.0(2)
C10	C11	C12	C13	-0.2(4)
C11	O5	C15	C16	-172.5(2)
C11	C12	C13	C7	-0.7(4)
C11	C12	C13	C14	178.6(2)
C13	C7	C8	C9	-179.9(2)
C13	C7	C8	C10	-0.5(4)
C15	O5	C11	C10	-179.6(2)
C15	O5	C11	C12	0.7(4)
C16	O6	C17	C18	2.3(4)
C16	O6	C17	C24	-177.5(2)
C17	O6	C16	C15	169.7(2)
C17	C18	C19	C20	-177.6(2)
C17	C18	C19	C21	0.4(4)
C18	C17	C24	C22	-2.5(4)
C18	C19	C21	N7	180.0(2)
C18	C19	C21	C22	-2.1(4)
C19	C21	C22	C23	-178.5(2)
C19	C21	C22	C24	1.5(4)
C20	C19	C21	N7	-2.0(4)
C20	C19	C21	C22	175.9(2)

Table S8. Torsion Angles for $4a_2$.

A	B	C	D	Angle/°	A	B	C	D	Angle/°
N4	N3	C3	N2	148.2(2)	C21	N7	N9	N10	79.4(3)
N4	N6	C6	O4	-170.9(2)	C21	N7	N9	C27	-150.5(2)
N4	N6	C6	N5	9.6(2)	C21	N7	C25	O7	-33.5(4)
N4	N6	C7	C8	76.2(3)	C21	N7	C25	N8	145.6(2)
N4	N6	C7	C13	-102.4(2)	C21	C22	C24	C17	0.8(4)
N6	N4	C4	O3	-167.9(2)	C23	C22	C24	C17	-179.1(2)
N6	N4	C4	N5	13.1(2)	C24	C17	C18	C19	1.9(4)
N6	C7	C8	C9	1.6(3)	C25	N7	N9	N10	-141.4(2)
N6	C7	C8	C10	-179.0(2)	C25	N7	N9	C27	-11.3(2)
N6	C7	C13	C12	179.6(2)	C25	N7	C21	C19	113.0(3)
N6	C7	C13	C14	0.3(3)	C25	N7	C21	C22	-64.9(3)
N7	N9	N10	N12	-135.0(2)	C25	N8	C27	O8	175.6(3)
N7	N9	N10	C28	100.4(2)	C25	N8	C27	N9	-5.4(3)
N7	N9	C27	O8	-170.9(2)	C26	N8	C25	O7	-4.2(4)
N7	N9	C27	N8	10.1(3)	C26	N8	C25	N7	176.6(2)
N7	C21	C22	C23	-0.7(4)	C26	N8	C27	O8	-2.6(4)
N7	C21	C22	C24	179.3(2)	C26	N8	C27	N9	176.4(2)
N9	N7	C21	C19	-113.2(2)	C27	N8	C25	O7	177.5(3)
N9	N7	C21	C22	68.9(3)	C27	N8	C25	N7	-1.6(3)
N9	N7	C25	O7	-171.2(2)	C27	N9	N10	N12	99.8(2)
N9	N7	C25	N8	7.9(3)	C27	N9	N10	C28	-24.7(3)
N9	N10	N12	C30	-144.1(2)	C28	N10	N12	C30	-13.6(3)
N9	N10	N12	C31	64.9(3)	C28	N10	N12	C31	-164.6(2)
N9	N10	C28	O9	-41.9(4)	C28	N11	C30	O10	177.5(3)
N9	N10	C28	N11	138.7(2)	C28	N11	C30	N12	-3.1(3)
N10	N9	C27	O8	-42.7(3)	C29	N11	C28	O9	-2.5(5)
N10	N9	C27	N8	138.3(2)	C29	N11	C28	N10	176.8(2)
N10	N12	C30	O10	-170.4(3)	C29	N11	C30	O10	-4.6(4)
N10	N12	C30	N11	10.2(3)	C29	N11	C30	N12	174.8(2)
N10	N12	C31	C32	67.7(3)	C30	N11	C28	O9	175.4(3)
N10	N12	C31	C37	-105.8(3)	C30	N11	C28	N10	-5.3(3)
N12	N10	C28	O9	-169.4(3)	C30	N12	C31	C32	-76.5(3)
N12	N10	C28	N11	11.3(3)	C30	N12	C31	C37	110.1(3)
N12	C31	C32	C33	6.4(4)	C31	N12	C30	O10	-24.1(4)
N12	C31	C32	C34	-170.7(2)	C31	N12	C30	N11	156.5(2)
N12	C31	C37	C36	170.1(2)	C31	C32	C34	C35	1.9(4)
N12	C31	C37	C38	-10.1(4)	C32	C31	C37	C36	-3.1(4)
C1	N1	N3	N4	-153.4(2)	C32	C31	C37	C38	176.7(2)
C1	N1	N3	C3	-16.9(3)	C32	C34	C35	O11	175.1(2)
C1	N1	C45	C43	-64.8(3)	C32	C34	C35	C36	-5.4(4)

Table S8. Torsion Angles for 4a₂.

A	B	C	D	Angle/°	A	B	C	D	Angle/°
C1	N1	C45	C46	118.8(3)	C33	C32	C34	C35	-175.3(2)
C1	N2	C3	O2	174.6(3)	C34	C35	C36	C37	4.6(4)
C1	N2	C3	N3	-7.2(3)	C35	O11	C39	C40	160.7(2)
C2	N2	C1	O1	-6.3(4)	C35	C36	C37	C31	-0.4(4)
C2	N2	C1	N1	174.3(2)	C35	C36	C37	C38	179.8(2)
C2	N2	C3	O2	-2.9(4)	C37	C31	C32	C33	179.5(2)
C2	N2	C3	N3	175.3(2)	C37	C31	C32	C34	2.4(4)
C3	N2	C1	O1	176.1(3)	C39	O11	C35	C34	-155.0(2)
C3	N2	C1	N1	-3.3(3)	C39	O11	C35	C36	25.5(3)
C3	N3	N4	N6	98.8(2)	C40	O12	C41	C42	6.3(4)
C3	N3	N4	C4	-27.9(3)	C40	O12	C41	C48	-172.9(2)
C4	N4	N6	C6	-14.3(2)	C41	O12	C40	C39	-179.1(2)
C4	N4	N6	C7	-156.6(2)	C41	C42	C43	C44	-179.9(2)
C4	N5	C6	O4	179.1(3)	C41	C42	C43	C45	1.3(3)
C4	N5	C6	N6	-1.5(3)	C42	C41	C48	C46	2.9(4)
C5	N5	C4	O3	-8.4(4)	C42	C43	C45	N1	-174.1(2)
C5	N5	C4	N4	170.5(2)	C42	C43	C45	C46	2.1(3)
C5	N5	C6	O4	1.1(4)	C43	C45	C46	C47	176.7(2)
C5	N5	C6	N6	-179.4(2)	C43	C45	C46	C48	-3.1(4)
C6	N5	C4	O3	173.7(2)	C44	C43	C45	N1	7.2(3)
C6	N5	C4	N4	-7.4(3)	C44	C43	C45	C46	-176.6(2)
C6	N6	C7	C8	-59.7(3)	C45	N1	N3	N4	58.9(3)
C6	N6	C7	C13	121.7(2)	C45	N1	N3	C3	-164.6(2)
C7	N6	C6	O4	-31.3(4)	C45	N1	C1	O1	-23.0(4)
C7	N6	C6	N5	149.2(2)	C45	N1	C1	N2	156.5(2)
C7	C8	C10	C11	-0.4(4)	C45	C46	C48	C41	0.6(4)
C8	C7	C13	C12	1.1(4)	C47	C46	C48	C41	-179.2(2)
C8	C7	C13	C14	-178.2(2)	C48	C41	C42	C43	-3.8(4)

Table S9. Hydrogen Atom Coordinates (Å×10⁴) and Isotropic Displacement Parameters (Å²×10³) for 4a₂.

Atom	*x*	*y*	*z*	U(eq)
H49A	6799.91	12143.45	-1655.37	44
H49B	7063.28	12981.16	-2169.96	44
H2A	3444.37	9625.26	1671.6	41
H2B	1699	9576.8	1437.03	41
H2C	2729.03	10435.66	1208.24	41
H5A	1607.08	4985.77	232.56	47

Table S9. Hydrogen Atom Coordinates (Å×10⁴) and Isotropic Displacement Parameters (Å²×10³) for 4a₂.

Atom	*x*	*y*	*z*	U(eq)
H5B	974.16	4775.41	-507.03	47
H5C	-93.23	5266.4	19.39	47
H9A	4180.17	6377.64	-1307.84	36
H9B	4786.38	6221.55	-2041.42	36
H9C	3110.96	5896.73	-1867.58	36
H10	4465.56	7436.04	-2834.68	25
H12	1775.46	9687.26	-2503.48	25
H14A	1147.54	9701.6	-1098.69	34
H14B	136.88	8774.92	-1035.28	34
H14C	-193.63	9497.13	-1632.38	34
H15A	3553.68	10348.33	-3261.87	28
H15B	2045.24	9863.07	-3568.59	28
H16A	3520.3	9433.05	-4527.59	27
H16B	3190.98	10538.11	-4463.72	27
H18	4329.42	9982.65	-5457.88	26
H20A	4572.96	9609.56	-6626.87	42
H20B	6202.02	9595.07	-6952.46	42
H20C	5342.3	10571.78	-6851.34	42
H23A	10748.24	9877.99	-5331.86	33
H23B	10476.28	10550.96	-4709.23	33
H23C	10533.97	10983.99	-5442.61	33
H24	8077.16	10431.29	-4289.31	24
H26A	11332.21	11714.57	-7558.71	38
H26B	12607.27	10916.23	-7524.42	38
H26C	12437.26	11628.85	-6915.6	38
H29A	8487.75	6526.5	-8210.88	49
H29B	10259.59	6366.17	-8065.49	49
H29C	9688.17	7267.04	-8478.1	49
H33A	6125.78	8059.01	-6306.84	40
H33B	5470.55	7224.54	-5866.76	40
H33C	6709.83	7006.23	-6409.33	40
H34	6260.18	7681.53	-4769.07	27
H36	10698.79	8090.94	-4273.94	27
H38A	12152.57	7333.62	-5740.1	38
H38B	12592.9	7872.14	-5062.56	38
H38C	12122.86	8457.29	-5716.64	38
H39A	9297.96	9247.77	-3646.1	27
H39B	9745.96	8349.63	-3197.29	27
H40A	8382.82	9675.24	-2647.13	27

Table S9. Hydrogen Atom Coordinates (Å×10[4]) and Isotropic Displacement Parameters (Å[2]×10[3]) for 4a$_2$.

Atom	*x*	*y*	*z*	U(eq)
H40B	6897.04	9232.22	-3006.81	27
H42	6095.45	9782.17	-2012.68	24
H44A	4898.44	10687.76	-774.5	36
H44B	3452.87	10012.58	-753.53	36
H44C	3971.59	10436.13	-1447.64	36
H47A	6777.9	7446.26	254.47	42
H47B	7605.67	6787.57	-266.9	42
H47C	5810.79	6721.46	-194.47	42
H48	7942.69	7325.86	-1358.63	26

Experimental

Single crystals were recrystallized from a solution of Compound **4a$_2$** (GB2020_Dec) dissolved in a mixture of dichloromethane and methanol. A suitable crystal with dimensions 0.33 × 0.13 × 0.10 mm^3 was selected and mounted on a loop with paratone oil on a XtaLAB Synergy, Dualflex, HyPix diffractometer. The crystal was kept at 100 K during data collection. Using *Olex2* [5], the structure was solved with the *olex2.solve* [2] structure solution program and refined with *SHELXL* [3] via full matrix least squares minimization on F^2.

1. Dolomanov, O.V., Bourhis, L.J., Gildea, R.J, Howard, J.A.K. & Puschmann, H. (2009), *J. Appl. Cryst.* **42**, 339.
2. Bourhis, L.J., Dolomanov, O.V., Gildea, R.J, Howard, J.A.K. & Puschmann, H. (2015), *Acta Cryst.* A**71**, 59.
3. Sheldrick, G. M. (2015), *Acta Cryst. C71*, 3.

Crystal structure determination of 4a$_2$

Crystal Data for $C_{49}H_{54}Cl_2N_{12}O_{12}$ (*M* =1073.94 g/mol): monoclinic, space group P2$_1$ (no. 4), *a* = 8.83819(7) Å, *b* = 14.23369(11) Å, *c* = 20.10392(16) Å, *β* = 91.7994(7)°, *V* = 2527.83(3) Å^3, *Z* = 2, *T* = 100.15 K, μ(CuKα) = 1.792 mm^{-1}, *Dcalc* = 1.411 g/cm^3, 35964 reflections measured (4.398° ≤ 2Θ ≤ 155.172°), 10128 unique (R_{int} = 0.0441, R_{sigma} = 0.0387) which were used in all calculations. The final R_1 was 0.0338 (I > 2σ(I)) and wR_2 was 0.0896 (all data).

Refinement model description

Number of restraints - 1, number of constraints - unknown.
Details:

```
1. Twinned data refinement
 Scales: 0.50905
 0.49095
2.a Secondary CH2 refined with riding coordinates:
 C49(H49A,H49B), C15(H15A,H15B), C16(H16A,H16B), C39(H39A,H39B), C40(H40A,H40B)
2.b Aromatic/amide H refined with riding coordinates:
 C10(H10), C12(H12), C18(H18), C24(H24), C34(H34), C36(H36), C42(H42), C48(H48)
2.c Idealised Me refined as rotating group:
 C2(H2A,H2B,H2C), C5(H5A,H5B,H5C), C9(H9A,H9B,H9C), C14(H14A,H14B,H14C),
 C20(H20A,H20B,H20C), C23(H23A,H23B,H23C), C26(H26A,H26B,H26C), C29(H29A,H29B,
 H29C), C33(H33A,H33B,H33C), C38(H38A,H38B,H38C), C44(H44A,H44B,H44C), C47(H47A,
 H47B,H47C)
```

This report has been created with Olex2, compiled on 2023.02.24 svn.rf166f9f3 for OlexSys. Please let us know if there are any errors or if you would like to have additional features.

Crystal Structure Data for Dimer $4b_2$

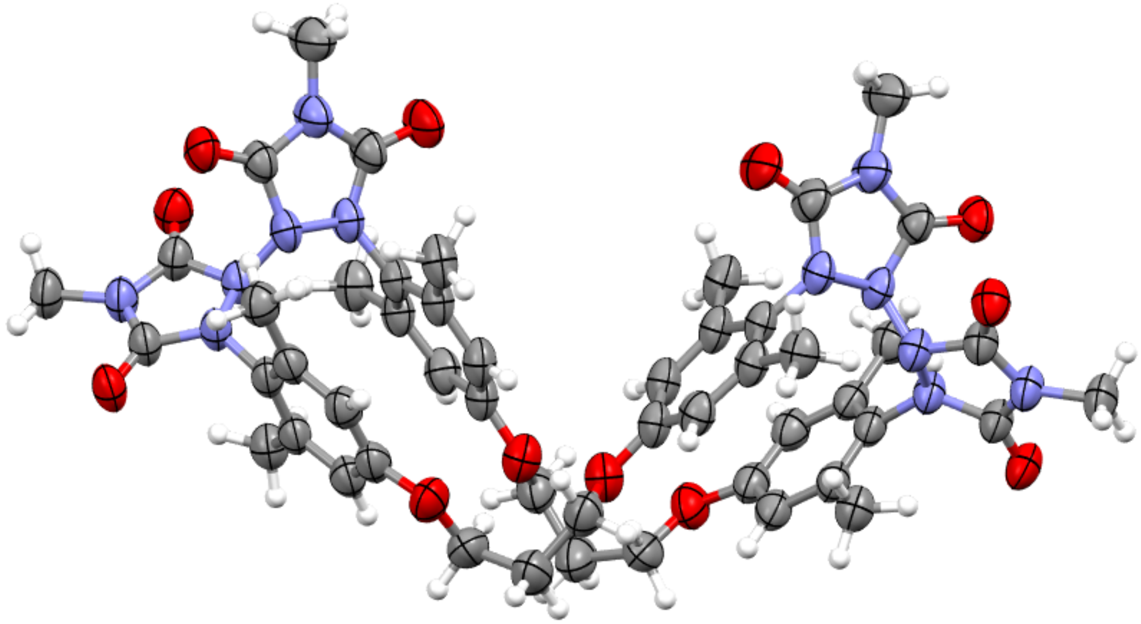

*elipsoids represent 50% probability level

Table S10. Crystal data and structure refinement for $4b_2$.

Identification code	GB-2021-01
Empirical formula	$C_{53}H_{59}Cl_9N_{12}O_{12}$
Formula weight	1375.17
Temperature/K	109(2)
Crystal system	monoclinic
Space group	I2/a
a/Å	27.2958(4)
b/Å	17.6590(5)
c/Å	27.3191(6)
α/°	90
β/°	95.6702(16)
γ/°	90
Volume/Å^3	13103.8(5)
Z	8
ρ_{calc}g/cm^3	1.394
μ/mm^{-1}	4.071
F(000)	5680.0
Crystal size/mm^3	0.373 × 0.3 × 0.212
Radiation	Cu Kα (λ = 1.54184)

2Θ range for data collection/°	6.502 to 130.176
Index ranges	-32 ≤ h ≤ 25, -20 ≤ k ≤ 20, -32 ≤ l ≤ 32
Reflections collected	76750
Independent reflections	11103 [R_{int} = 0.0541, R_{sigma} = 0.0334]
Data/restraints/parameters	11103/1592/976
Goodness-of-fit on F^2	1.060
Final R indexes [I>=2σ (I)]	R_1 = 0.0707, wR_2 = 0.2089
Final R indexes [all data]	R_1 = 0.0802, wR_2 = 0.2169
Largest diff. peak/hole / e Å^{-3}	0.42/-0.43

Table S11. Fractional Atomic Coordinates (×10^4) and Equivalent Isotropic Displacement Parameters (Å^2×10^3) for 4b$_2$. U_{eq} is defined as 1/3 of the trace of the orthogonalised U_{IJ} tensor.

Atom	*x*	*y*	*z*	U(eq)
C21	3143.3(15)	158(2)	4348.9(11)	87.4(11)
C22	3073(2)	7066(4)	5507(2)	60.3(16)
C6	3116(3)	6986(4)	5808(3)	64.8(17)
O1_1	3759.2(8)	825.1(12)	4834.5(6)	75.0(6)
O2_1	5282.6(7)	1631.3(12)	6774.8(7)	69.5(5)
O3_1	4374.7(7)	3444.9(12)	7470.8(7)	69.6(5)
N1_1	4604.1(6)	2333.9(11)	6481.9(6)	56.1(5)
N2_1	4274.3(7)	2811.8(12)	6723.7(7)	57.4(5)
N3_1	4915.4(7)	2516.3(13)	7250.1(7)	62.3(6)
C2_1	3361.3(11)	327.8(17)	4864.6(11)	76.3(9)
C3_1	3985.5(9)	1149.2(14)	5251.1(8)	64.1(7)
C4_1	3871.3(9)	928.8(15)	5714.8(8)	63.1(7)
C5_1	4080.1(9)	1305.7(14)	6130.6(8)	59.5(6)
C6_1	4405.0(8)	1901.1(13)	6065.9(7)	57.0(6)
C7_1	4538.6(9)	2110.1(14)	5606.7(7)	59.7(6)
C8_1	4323.7(10)	1721.9(15)	5199.7(8)	64.5(7)
C9_1	3941.2(11)	1093.9(18)	6629.8(9)	68.1(8)
C10_1	4895.0(10)	2741.2(16)	5540.4(10)	67.9(7)
C11_1	4970.6(8)	2108.4(15)	6825.9(8)	59.9(7)
C12_1	4516.7(8)	2979.0(15)	7190.5(8)	60.3(7)
C13_1	5260.4(10)	2492(2)	7690.1(10)	74.6(9)
O1_2	2597.3(7)	1198.5(12)	4338.2(7)	73.1(6)
O2_2	1875.0(6)	4635.6(12)	3761.9(11)	86.0(7)
O3_2	229.4(5)	4214.5(11)	3379.3(8)	68.2(5)
N1_2	1417.0(5)	3552.4(10)	3581.5(7)	56.2(5)

Table S11. Fractional Atomic Coordinates (×10[4]) and Equivalent Isotropic Displacement Parameters (Å[2]×10[3]) for 4b$_2$. U_{eq} is defined as 1/3 of the trace of the orthogonalised U_{IJ} tensor.

Atom	*x*	*y*	*z*	U(eq)
N2_2	894.7(6)	3415.2(10)	3566.0(8)	55.3(5)
N3_2	1027.5(6)	4647.5(10)	3589.3(10)	68.0(6)
C2_2	2940.3(11)	836.8(18)	4065.2(11)	75.2(9)
C3_2	2334.3(9)	1804.4(14)	4143.3(8)	61.8(7)
C4_2	2066.2(9)	2215.4(15)	4460.5(8)	63.8(7)
C5_2	1775.5(9)	2818.7(14)	4287.0(8)	61.0(7)
C6_2	1742.3(7)	2970.0(13)	3782.8(7)	55.4(6)
C7_2	2014.2(8)	2580.1(14)	3460.3(8)	59.4(7)
C8_2	2318.9(9)	1998.9(15)	3650.6(8)	61.9(7)
C9_2	1512.5(10)	3290.7(18)	4634.1(9)	70.4(8)
C10_2	1980.1(10)	2777.6(19)	2922.7(8)	69.0(8)
C11_2	1488.1(7)	4309.1(12)	3654.2(12)	66.0(7)
C12_2	666.2(7)	4125.7(12)	3494.0(11)	58.7(6)
C13_2	952.6(11)	5459.9(13)	3600.4(19)	93.5(12)
O1_3	3170.0(10)	6066.2(18)	4969.9(13)	60.1(9)
O2_3	4106(10)	2907(5)	4377.1(18)	86(3)
O3_3	4035(2)	3133(3)	2706.1(15)	67.6(12)
N1_3	4065.9(16)	3942(3)	3860(2)	54.9(7)
N2_3	3956(4)	3998(3)	3334(2)	53.0(15)
N3_3	4082(4)	2796(3)	3531.4(16)	61.4(15)
C2_3	3442(2)	6640(3)	5230(3)	66(2)
C3_3	3400.1(15)	5544(4)	4704(4)	56.2(18)
C4_3	3113.1(12)	4980(2)	4462.2(18)	55.8(11)
C5_3	3326.8(13)	4438(2)	4184.7(19)	51.3(12)
C6_3	3834.3(13)	4482(4)	4157(3)	54.5(15)
C7_3	4131.8(14)	5017(5)	4412(4)	58.7(18)
C8_3	3905.2(13)	5558(3)	4681.5(19)	60.1(14)
C9_3	3020.2(16)	3815(3)	3941(2)	59.2(13)
C10_3	4679.4(15)	5031(7)	4387(5)	65(2)
C11_3	4114(4)	3190(4)	3972.4(16)	70.0(17)
C12_3	4030(3)	3273(3)	3140.7(16)	53.9(17)
C13_3	4117(3)	1978(3)	3499(3)	76(2)
O1_4	2445.6(14)	6905(3)	6030(2)	65.1(15)
O2_4	1055(3)	6037(4)	7788(3)	62.5(19)
O3_4	179(4)	4118(7)	6994(5)	63.5(13)
N1_4	1193.6(13)	5294(3)	7120.7(19)	55.9(10)
N2_4	880(3)	4774(6)	6828(3)	56.2(9)
N3_4	511(3)	5107(7)	7480(4)	57.5(16)

Table S11. Fractional Atomic Coordinates (×10^4) and Equivalent Isotropic Displacement Parameters (Å^2×10^3) for 4b₂. U_{eq} is defined as 1/3 of the trace of the orthogonalised U_{IJ} tensor.

Atom	*x*	*y*	*z*	U(eq)
C2_4	2886.6(16)	6578(4)	5907(2)	62.6(14)
C3_4	2153.6(12)	6533(2)	6328.9(17)	55.7(11)
C4_4	1669.0(13)	6775(2)	6335.1(19)	57.7(13)
C5_4	1343.5(13)	6374(3)	6595(3)	49.9(14)
C6_4	1525.0(11)	5749(3)	6870(2)	51.6(16)
C7_4	2014.4(12)	5522(2)	6892.3(17)	52.7(11)
C8_4	2321.2(19)	5906(6)	6600(5)	51(2)
C9_4	818.3(18)	6626(5)	6587(4)	62(2)
C10_4	2208.4(17)	4877(3)	7214(2)	61.3(14)
C11_4	936(3)	5541(5)	7493(3)	59(2)
C12_4	482(3)	4609(6)	7100(4)	56.8(9)
C13_4	139(4)	5195(10)	7820(6)	76(3)
O1_5	2710.3(10)	6343(2)	6383.0(14)	68.2(10)
O2_5	967(3)	5793(4)	7865(3)	67(2)
O3_5	59(3)	4044(7)	6934(5)	63.5(13)
N1_5	1124.0(12)	5068(3)	7193.7(19)	55.9(10)
N2_5	800(3)	4645(7)	6845(3)	56.2(9)
N3_5	397(3)	4963(7)	7477(4)	59.6(17)
C2_5	2652(3)	6969(4)	6066(3)	84(3)
C3_5	2324.9(18)	6085(6)	6620(5)	60(2)
C4_5	1857.6(13)	6391(3)	6509.3(19)	62.0(13)
C5_5	1455.6(12)	6082(3)	6711(2)	53.9(17)
C6_5	1533.9(11)	5447(3)	7014(2)	54.5(14)
C7_5	1997.2(12)	5128(3)	7127(2)	61.1(14)
C8_5	2389.8(12)	5448(2)	6915.7(18)	63.1(13)
C9_5	955.5(17)	6428(5)	6600(4)	61.5(19)
C10_5	2079.0(17)	4466(3)	7470(2)	65.3(14)
C11_5	845(2)	5336(5)	7543(3)	53.2(17)
C12_5	370(3)	4506(7)	7076(4)	56.8(9)
C13_5	16(4)	5029(9)	7810(5)	77(3)
O1_6	3576.0(12)	6343(2)	5226.1(17)	56.5(12)
O2_6	4148(10)	3062(5)	4419.1(17)	75(3)
O3_6	3860(2)	3369(3)	2755.0(14)	67.6(12)
N1_6	4060.6(17)	4122(3)	3923.2(19)	54.9(7)
N2_6	3900(4)	4215(3)	3407.5(19)	52.6(15)
N3_6	3980(4)	2993(2)	3572.7(16)	61.4(15)
C2_6	3124.6(16)	6336(3)	5432(2)	57.2(13)
C3_6	3686.2(12)	5794(2)	4902.4(16)	51.0(11)

Table S11. Fractional Atomic Coordinates (×10⁴) and Equivalent Isotropic Displacement Parameters (Å²×10³) for 4b₂. U_{eq} is defined as 1/3 of the trace of the orthogonalised U_{IJ} tensor.

Atom	*x*	*y*	*z*	U(eq)
C4_6	3324.8(15)	5299(4)	4702(3)	50.3(18)
C5_6	3437.2(13)	4749(3)	4367.1(18)	51.6(12)
C6_6	3924.3(14)	4699(3)	4254(2)	53.1(16)
C7_6	4294.6(15)	5176(4)	4457(3)	50.0(14)
C8_6	4167.0(13)	5727(3)	4783.8(17)	52.4(12)
C9_6	3048.4(17)	4213(3)	4152(2)	58.4(14)
C10_6	4819.1(17)	5086(6)	4344(5)	61(2)
C11_6	4062(3)	3364(3)	4020.5(15)	51.0(13)
C12_6	3914(3)	3491(3)	3194.0(15)	53.4(18)
C13_6	3972(3)	2175(3)	3520(2)	70.0(17)

Table S12. Anisotropic Displacement Parameters (Å²×10³) for 4b₂. The Anisotropic displacement factor exponent takes the form: $-2\pi^2[h^2a^{*2}U_{11}+2hka^*b^*U_{12}+\ldots]$.

Atom	U_{11}	U_{22}	U_{33}	U_{23}	U_{13}	U_{12}
C21	92(2)	81(2)	84(2)	-14.0(19)	-20.2(19)	12.0(19)
C22	43(3)	64(4)	75(4)	0(4)	8(3)	-2(2)
C6	59(4)	57(4)	80(5)	-7(4)	16(4)	-5(3)
O1_1	77.2(13)	80.3(14)	63.9(12)	-14.6(10)	-11.7(10)	2.0(11)
O2_1	56.9(11)	85.5(14)	63.5(12)	2.2(10)	-7.7(9)	21.6(10)
O3_1	61.1(11)	84.3(14)	62.1(12)	-12.9(10)	-0.2(9)	7.3(10)
N1_1	45.5(11)	69.4(14)	51.0(12)	-5.7(10)	-6.6(9)	12.0(10)
N2_1	47.0(11)	71.5(15)	52.4(12)	-0.7(10)	-1.3(9)	9.3(10)
N3_1	53.1(12)	78.5(16)	52.4(12)	-3.6(11)	-9.5(10)	8.8(11)
C2_1	85(2)	61.7(19)	78(2)	-8.9(16)	-12.2(17)	1.8(15)
C3_1	67.1(17)	64.2(17)	57.5(15)	-7.3(12)	-10.9(12)	14.7(13)
C4_1	58.5(16)	65.3(18)	63.2(15)	-2.9(13)	-5.9(12)	10.6(13)
C5_1	50.1(14)	66.2(17)	59.3(15)	0.4(12)	-8.4(11)	11.4(12)
C6_1	51.4(14)	63.7(16)	53.0(13)	-4.4(12)	-9.6(11)	11.5(11)
C7_1	60.0(15)	62.4(16)	54.3(14)	0.2(12)	-6.1(11)	14.3(12)
C8_1	68.1(17)	68.7(18)	54.7(15)	-4.3(13)	-3.5(13)	11.0(13)
C9_1	60.6(17)	79(2)	62.9(16)	2.1(15)	-1.7(13)	6.2(15)
C10_1	69.0(18)	76(2)	57.8(16)	-0.2(14)	-0.7(14)	4.4(14)
C11_1	48.0(14)	74.0(18)	55.4(14)	-0.4(13)	-6.0(11)	11.6(12)
C12_1	50.8(14)	76.3(18)	52.0(14)	-5.4(13)	-3.7(11)	4.3(12)
C13_1	62.4(17)	98(2)	59.1(17)	-3.3(16)	-17.7(14)	5.8(16)
O1_2	59.4(11)	89.9(15)	68.0(12)	4.2(11)	-2.7(9)	4.9(10)

Table S12. Anisotropic Displacement Parameters (Å^2×10^3) for 4b$_2$. The Anisotropic displacement factor exponent takes the form: -2π^2[h^{2}a*^{2}U$_{11}$+2hka*b*U$_{12}$+…].

Atom	U$_{11}$	U$_{22}$	U$_{33}$	U$_{23}$	U$_{13}$	U$_{12}$
O2_2	39.4(10)	84.4(15)	133(2)	-5.4(14)	3.3(11)	-15.5(10)
O3_2	39.3(9)	79.7(14)	84.3(14)	3.8(11)	0.4(9)	-3.9(9)
N1_2	34.9(10)	72.4(14)	61.0(13)	-2.5(11)	3.3(9)	-6.6(9)
N2_2	34.8(10)	69.1(14)	61.2(13)	-4.2(11)	1.0(9)	-8.5(9)
N3_2	40.0(11)	68.2(14)	95.6(19)	-5.4(13)	5.8(11)	-5.9(10)
C2_2	67.6(18)	83(2)	71.8(19)	-15.3(17)	-7.2(15)	4.7(16)
C3_2	40.9(13)	80.8(19)	61.8(15)	-0.5(13)	-3.7(11)	-4.5(12)
C4_2	44.3(14)	90(2)	55.5(15)	-0.2(14)	-2.3(11)	-7.2(12)
C5_2	40.4(13)	87(2)	55.1(14)	-4.9(13)	-0.1(11)	-8.2(12)
C6_2	36.9(12)	73.3(17)	54.9(13)	-4.8(12)	-0.2(10)	-7.7(11)
C7_2	36.5(12)	84.2(19)	57.6(14)	-5.0(13)	5.4(10)	-6.5(12)
C8_2	44.3(13)	81.1(19)	60.6(15)	-7.5(13)	6.8(11)	-2.4(12)
C9_2	49.3(15)	105(2)	56.2(16)	-13.3(16)	2.9(12)	-10.2(15)
C10_2	53.1(15)	96(2)	59.0(16)	-2.4(15)	10.6(13)	-2.7(15)
C11_2	40.4(13)	74.8(17)	83(2)	-5.6(15)	5.8(12)	-9.5(12)
C12_2	40.5(12)	66.7(16)	68.5(17)	-2.8(13)	3.0(11)	-6.9(11)
C13_2	55.5(18)	70(2)	153(4)	-4(2)	4(2)	-1.5(15)
O1_3	39.1(17)	72(2)	70(2)	-3.3(18)	9.0(16)	-2.0(15)
O2_3	87(6)	111(6)	64(3)	21(3)	18(4)	24(7)
O3_3	78(4)	76(4)	49.8(13)	-7.9(16)	10.7(17)	-8(2)
N1_3	47.6(12)	78(2)	39.1(15)	5.3(13)	4.1(10)	11.3(14)
N2_3	49(3)	71(4)	39(2)	3(3)	8(2)	-5(4)
N3_3	62(4)	71(2)	53.4(14)	5.6(15)	16.5(16)	5(3)
C2_3	60(4)	68(5)	70(4)	0(4)	8(3)	-14(4)
C3_3	48(3)	67(5)	54(4)	11(3)	12(3)	8(2)
C4_3	40(3)	72(3)	56(3)	11(2)	7(2)	9(2)
C5_3	39(3)	66(4)	49(3)	14(2)	8(3)	4(2)
C6_3	42(2)	79(4)	43(4)	9(3)	6(2)	9(2)
C7_3	38(3)	91(5)	47(4)	7(3)	8(4)	6(3)
C8_3	43(3)	89(4)	49(3)	6(3)	8(3)	5(3)
C9_3	43(3)	79(4)	56(3)	6(3)	7(2)	-2(3)
C10_3	42(3)	109(6)	46(4)	-11(4)	9(4)	-8(4)
C11_3	80(5)	76(3)	57(2)	4(2)	22(2)	16(3)
C12_3	38(4)	75(4)	49(2)	0(2)	9(2)	3(3)
C13_3	65(5)	73(3)	92(5)	10(4)	27(4)	11(3)
O1_4	55(3)	62(3)	80(3)	-1(2)	12(2)	0(2)
O2_4	64(4)	79(5)	43(3)	-7(3)	0(2)	2(3)
O3_4	44(4)	83(3)	63(3)	2.6(16)	4(3)	0(2)
N1_4	37.8(14)	82(3)	47.1(17)	-9.8(17)	-0.1(11)	-0.2(15)

Table S12. Anisotropic Displacement Parameters (Å^2×10^3) for 4b$_2$. The Anisotropic displacement factor exponent takes the form: $-2\pi^2[h^2a^{*2}U_{11}+2hka^*b^*U_{12}+\ldots]$.

Atom	U_{11}	U_{22}	U_{33}	U_{23}	U_{13}	U_{12}
N2_4	33(2)	83(3)	51.7(13)	-15.8(17)	-0.9(13)	-0.1(14)
N3_4	49(4)	77(5)	47(3)	1(3)	7(3)	6(3)
C2_4	52(3)	68(4)	69(4)	-5(3)	9(3)	5(3)
C3_4	43(2)	60(3)	65(3)	-12(2)	6(2)	-2(2)
C4_4	42(2)	62(4)	69(4)	-9(3)	6(2)	0(2)
C5_4	32(3)	64(4)	52(4)	-9(3)	-3(3)	4(2)
C6_4	40(2)	77(6)	36(5)	-7(3)	-2(2)	3(2)
C7_4	42(2)	62(3)	53(3)	-10(2)	-2(2)	2(2)
C8_4	39(3)	58(5)	56(4)	-22(4)	2(2)	-2(2)
C9_4	35(3)	85(6)	65(4)	-1(4)	8(3)	14(3)
C10_4	49(3)	64(4)	70(4)	-6(3)	1(3)	3(3)
C11_4	56(3)	73(5)	49(3)	-4(3)	6(2)	2(3)
C12_4	34(3)	83(3)	52.1(16)	0.0(17)	0(2)	5.7(18)
C13_4	73(5)	100(10)	58(5)	13(5)	21(4)	10(5)
O1_5	44.1(18)	75(2)	87(3)	-26(2)	13.2(18)	-9.0(18)
O2_5	64(4)	84(5)	51(3)	-11(3)	2(2)	10(3)
O3_5	44(4)	83(3)	63(3)	2.6(16)	4(3)	0(2)
N1_5	37.8(14)	82(3)	47.1(17)	-9.8(17)	-0.1(11)	-0.2(15)
N2_5	33(2)	83(3)	51.7(13)	-15.8(17)	-0.9(13)	-0.1(14)
N3_5	46(3)	82(5)	53(3)	0(3)	12(3)	12(3)
C2_5	91(7)	62(4)	102(6)	-26(3)	25(6)	-27(5)
C3_5	45(3)	65(5)	72(5)	-33(4)	10(3)	-10(2)
C4_5	49(3)	67(4)	72(4)	-17(3)	14(3)	-1(2)
C5_5	34(3)	76(6)	50(6)	-13(3)	-2(3)	4(3)
C6_5	44(2)	75(5)	43(4)	-11(3)	-2(2)	0(2)
C7_5	45(3)	83(5)	54(4)	-10(3)	-1(3)	6(3)
C8_5	49(3)	81(4)	59(3)	-22(3)	4(2)	0(3)
C9_5	40(3)	79(6)	67(4)	-6(4)	11(4)	7(4)
C10_5	48(3)	83(4)	65(3)	-3(3)	2(3)	11(3)
C11_5	45(4)	70(5)	44(3)	2(3)	-1(2)	19(3)
C12_5	34(3)	83(3)	52.1(16)	0.0(17)	0(2)	5.7(18)
C13_5	91(7)	76(7)	71(6)	17(5)	46(6)	30(5)
O1_6	49(2)	61(3)	60(3)	-4(2)	7(2)	-1(2)
O2_6	91(7)	88(4)	48(3)	15(2)	15(3)	26(5)
O3_6	78(4)	76(4)	49.8(13)	-7.9(16)	10.7(17)	-8(2)
N1_6	47.6(12)	78(2)	39.1(15)	5.3(13)	4.1(10)	11.3(14)
N2_6	52(4)	69(3)	36(2)	-2(2)	0(2)	-2(3)
N3_6	62(4)	71(2)	53.4(14)	5.6(15)	16.5(16)	5(3)
C2_6	44(2)	55(3)	74(4)	-5(3)	13(2)	0(2)

Table S12. Anisotropic Displacement Parameters (Å^2×10^3) for 4b$_2$. The Anisotropic displacement factor exponent takes the form: $-2\pi^2[h^2a^{*2}U_{11}+2hka^*b^*U_{12}+\ldots]$.

Atom	U_{11}	U_{22}	U_{33}	U_{23}	U_{13}	U_{12}
C3_6	40(2)	65(3)	49(3)	3(2)	8(2)	5(2)
C4_6	43(3)	59(4)	49(3)	9(3)	3(3)	9(2)
C5_6	48(3)	60(3)	47(3)	7(2)	2(2)	6(2)
C6_6	48(3)	77(5)	34(4)	3(3)	2(3)	7(3)
C7_6	36(3)	72(4)	43(3)	10(2)	6(3)	10(3)
C8_6	37(2)	70(3)	50(3)	2(2)	5(2)	3(2)
C9_6	53(3)	68(4)	54(4)	2(3)	7(3)	-1(3)
C10_6	39(3)	98(6)	47(4)	2(4)	11(4)	19(4)
C11_6	35(3)	78(3)	42(2)	6(2)	14(2)	17(3)
C12_6	48(5)	67(4)	47(2)	-2(2)	13(2)	-6(4)
C13_6	80(5)	76(3)	57(2)	4(2)	22(2)	16(3)

Table S13. Bond Lengths for 4b$_2$.

Atom	Atom	Length/Å	Atom	Atom	Length/Å
C21	C2_1	1.504(3)	C5_3	C9_3	1.498(3)
C21	C2_2	1.503(3)	C6_3	C7_3	1.389(3)
C22	C2_3	1.518(6)	C7_3	C8_3	1.389(3)
C22	C2_4	1.518(6)	C7_3	C10_3	1.503(3)
C6	C2_5	1.510(10)	O1_4	C2_4	1.405(3)
C6	C2_6	1.540(8)	O1_4	C3_4	1.366(3)
O1_1	C2_1	1.405(3)	O2_4	C11_4	1.215(2)
O1_1	C3_1	1.366(2)	O3_4	C12_4	1.214(2)
O2_1	C11_1	1.216(2)	N1_4	N2_4	1.443(2)
O3_1	N1_1	3.446(2)	N1_4	C6_4	1.435(2)
O3_1	C12_1	1.213(2)	N1_4	C11_4	1.364(2)
N1_1	N2_1	1.4411(19)	N2_4	C12_4	1.406(2)
N1_1	C6_1	1.431(2)	N3_4	C11_4	1.386(3)
N1_1	C11_1	1.363(2)	N3_4	C12_4	1.357(2)
N2_1	C12_1	1.409(2)	N3_4	C13_4	1.452(3)
N2_1	N2_2[1]	1.379(3)	C3_4	C4_4	1.392(3)
N3_1	C11_1	1.385(2)	C3_4	C8_4	1.385(3)
N3_1	C12_1	1.358(2)	C4_4	C5_4	1.385(3)
N3_1	C13_1	1.453(2)	C5_4	C6_4	1.397(3)
C3_1	C4_1	1.390(3)	C5_4	C9_4	1.499(3)
C3_1	C8_1	1.386(3)	C6_4	C7_4	1.390(3)
C4_1	C5_1	1.389(3)	C7_4	C8_4	1.389(3)
C5_1	C6_1	1.398(3)	C7_4	C10_4	1.503(3)

Table S13. Bond Lengths for $4b_2$.

Atom	Atom	Length/Å	Atom	Atom	Length/Å
C5_1	C9_1	1.499(3)	O1_5	C2_5	1.405(3)
C6_1	C7_1	1.390(3)	O1_5	C3_5	1.366(3)
C7_1	C8_1	1.386(3)	O2_5	C11_5	1.214(2)
C7_1	C10_1	1.502(3)	O3_5	C12_5	1.213(2)
O1_2	C2_2	1.407(2)	N1_5	N2_5	1.443(2)
O1_2	C3_2	1.367(2)	N1_5	C6_5	1.432(2)
O2_2	C11_2	1.214(2)	N1_5	C11_5	1.363(2)
O3_2	C12_2	1.213(2)	N2_5	C12_5	1.407(2)
N1_2	N2_2	1.4427(18)	N2_5	N2_6[1]	1.356(13)
N1_2	C6_2	1.432(2)	N3_5	C11_5	1.387(3)
N1_2	C11_2	1.362(2)	N3_5	C12_5	1.357(2)
N2_2	C12_2	1.406(2)	N3_5	C13_5	1.452(3)
N3_2	C11_2	1.387(2)	C3_5	C4_5	1.391(3)
N3_2	C12_2	1.356(2)	C3_5	C8_5	1.387(3)
N3_2	C13_2	1.450(2)	C4_5	C5_5	1.387(3)
C3_2	C4_2	1.392(3)	C5_5	C6_5	1.398(3)
C3_2	C8_2	1.386(3)	C5_5	C9_5	1.499(3)
C4_2	C5_2	1.384(3)	C6_5	C7_5	1.390(3)
C5_2	C6_2	1.397(3)	C7_5	C8_5	1.386(3)
C5_2	C9_2	1.498(3)	C7_5	C10_5	1.503(3)
C6_2	C7_2	1.389(2)	O1_6	C2_6	1.404(3)
C7_2	C8_2	1.389(3)	O1_6	C3_6	1.366(3)
C7_2	C10_2	1.503(3)	O2_6	C11_6	1.215(2)
O1_3	C2_3	1.406(3)	O3_6	C12_6	1.213(2)
O1_3	C3_3	1.366(3)	N1_6	N2_6	1.443(2)
O2_3	C11_3	1.216(3)	N1_6	C6_6	1.434(2)
O3_3	C12_3	1.214(2)	N1_6	C11_6	1.364(2)
N1_3	N2_3	1.443(2)	N2_6	C12_6	1.406(2)
N1_3	C6_3	1.437(2)	N3_6	C11_6	1.386(3)
N1_3	C11_3	1.366(2)	N3_6	C12_6	1.357(2)
N2_3	C12_3	1.406(2)	N3_6	C13_6	1.451(3)
N2_3	N2_4[1]	1.521(13)	C3_6	C4_6	1.389(3)
N3_3	C11_3	1.387(3)	C3_6	C8_6	1.388(3)
N3_3	C12_3	1.356(2)	C4_6	C5_6	1.388(3)
N3_3	C13_3	1.451(3)	C5_6	C6_6	1.397(3)
C3_3	C4_3	1.392(3)	C5_6	C9_6	1.499(3)
C3_3	C8_3	1.386(3)	C6_6	C7_6	1.390(3)
C4_3	C5_3	1.385(3)	C7_6	C8_6	1.387(3)
C5_3	C6_3	1.397(3)	C7_6	C10_6	1.503(3)

[1]1/2-X,+Y,1-Z

Table S14. Bond Angles for $4b_2$.

Atom	Atom	Atom	Angle/°	Atom	Atom	Atom	Angle/°
C2_2	C21	C2_1	114.5(3)	O3_3	C12_3	N3_3	129.2(3)
C2_3	C22	C2_4	111.3(6)	N3_3	C12_3	N2_3	106.13(19)
C2_5	C6	C2_6	111.4(6)	C3_4	O1_4	C2_4	120.6(3)
C3_1	O1_1	C2_1	120.11(18)	C6_4	N1_4	N2_4	117.4(2)
C12_1	O3_1	N1_1	12.84(12)	C11_4	N1_4	N2_4	107.16(18)
C6_1	N1_1	N2_1	118.12(15)	C11_4	N1_4	C6_4	124.9(3)
C11_1	N1_1	N2_1	107.71(14)	N1_4	N2_4	N2_3[1]	123.9(9)
C11_1	N1_1	C6_1	125.93(17)	C12_4	N2_4	N2_3[1]	103.4(6)
C12_1	N2_1	N1_1	106.20(13)	C12_4	N2_4	N1_4	106.60(18)
N2_2[1]	N2_1	N1_1	112.20(18)	C11_4	N3_4	C13_4	124.0(3)
N2_2[1]	N2_1	C12_1	117.26(19)	C12_4	N3_4	C11_4	111.40(18)
C11_1	N3_1	C13_1	124.14(18)	C12_4	N3_4	C13_4	124.6(3)
C12_1	N3_1	C11_1	111.39(15)	O1_4	C2_4	C22	107.1(4)
C12_1	N3_1	C13_1	124.33(19)	O1_4	C3_4	C4_4	118.1(2)
O1_1	C2_1	C21	107.8(3)	O1_4	C3_4	C8_4	121.6(2)
O1_1	C3_1	C4_1	121.23(19)	C8_4	C3_4	C4_4	120.2(2)
O1_1	C3_1	C8_1	118.18(19)	C5_4	C4_4	C3_4	120.5(2)
C8_1	C3_1	C4_1	120.56(19)	C4_4	C5_4	C6_4	117.9(2)
C5_1	C4_1	C3_1	120.0(2)	C4_4	C5_4	C9_4	120.1(3)
C4_1	C5_1	C6_1	118.10(19)	C6_4	C5_4	C9_4	122.0(2)
C4_1	C5_1	C9_1	120.3(2)	C5_4	C6_4	N1_4	119.5(2)
C6_1	C5_1	C9_1	121.57(19)	C7_4	C6_4	N1_4	117.8(2)
C5_1	C6_1	N1_1	119.80(18)	C7_4	C6_4	C5_4	122.7(2)
C7_1	C6_1	N1_1	117.50(18)	C6_4	C7_4	C10_4	121.7(2)
C7_1	C6_1	C5_1	122.68(18)	C8_4	C7_4	C6_4	117.7(2)
C6_1	C7_1	C10_1	122.45(19)	C8_4	C7_4	C10_4	120.5(2)
C8_1	C7_1	C6_1	117.7(2)	C3_4	C8_4	C7_4	120.7(3)
C8_1	C7_1	C10_1	119.87(19)	O2_4	C11_4	N1_4	127.0(3)
C3_1	C8_1	C7_1	120.9(2)	O2_4	C11_4	N3_4	125.8(3)
O2_1	C11_1	N1_1	126.73(19)	N1_4	C11_4	N3_4	107.17(18)
O2_1	C11_1	N3_1	126.29(18)	O3_4	C12_4	N2_4	124.5(2)
N1_1	C11_1	N3_1	106.97(15)	O3_4	C12_4	N3_4	129.3(3)
O3_1	C12_1	N2_1	124.24(18)	N3_4	C12_4	N2_4	106.22(19)
O3_1	C12_1	N3_1	129.38(19)	C3_5	O1_5	C2_5	120.6(3)
N3_1	C12_1	N2_1	106.37(15)	C6_5	N1_5	N2_5	117.7(2)
C3_2	O1_2	C2_2	120.03(18)	C11_5	N1_5	N2_5	107.1(2)
C6_2	N1_2	N2_2	117.64(15)	C11_5	N1_5	C6_5	125.9(3)

Table S14. Bond Angles for $4b_2$.

Atom	Atom	Atom	Angle/°	Atom	Atom	Atom	Angle/°
C11_2	N1_2	N2_2	107.16(14)	C12_5	N2_5	N1_5	106.12(19)
C11_2	N1_2	C6_2	125.06(17)	N2_6[1]	N2_5	N1_5	105.3(7)
N2_1[1]	N2_2	N1_2	114.84(17)	N2_6[1]	N2_5	C12_5	134.6(7)
N2_1[1]	N2_2	C12_2	119.37(17)	C11_5	N3_5	C13_5	123.9(3)
C12_2	N2_2	N1_2	106.12(14)	C12_5	N3_5	C11_5	111.32(19)
C11_2	N3_2	C13_2	123.47(18)	C12_5	N3_5	C13_5	124.7(3)
C12_2	N3_2	C11_2	111.38(16)	O1_5	C2_5	C6	104.8(6)
C12_2	N3_2	C13_2	125.10(18)	O1_5	C3_5	C4_5	120.0(3)
O1_2	C2_2	C21	108.6(2)	O1_5	C3_5	C8_5	119.1(3)
O1_2	C3_2	C4_2	116.99(19)	C8_5	C3_5	C4_5	120.2(3)
O1_2	C3_2	C8_2	122.46(19)	C5_5	C4_5	C3_5	120.4(2)
C8_2	C3_2	C4_2	120.5(2)	C4_5	C5_5	C6_5	118.0(2)
C5_2	C4_2	C3_2	120.5(2)	C4_5	C5_5	C9_5	120.0(2)
C4_2	C5_2	C6_2	117.66(19)	C6_5	C5_5	C9_5	122.0(2)
C4_2	C5_2	C9_2	120.39(19)	C5_5	C6_5	N1_5	120.0(2)
C6_2	C5_2	C9_2	121.95(19)	C7_5	C6_5	N1_5	117.3(2)
C5_2	C6_2	N1_2	119.55(17)	C7_5	C6_5	C5_5	122.6(2)
C7_2	C6_2	N1_2	117.51(17)	C6_5	C7_5	C10_5	122.0(2)
C7_2	C6_2	C5_2	122.93(18)	C8_5	C7_5	C6_5	117.8(2)
C6_2	C7_2	C10_2	121.27(19)	C8_5	C7_5	C10_5	120.2(2)
C8_2	C7_2	C6_2	117.80(19)	C3_5	C8_5	C7_5	120.9(2)
C8_2	C7_2	C10_2	120.93(19)	O2_5	C11_5	N1_5	127.3(3)
C3_2	C8_2	C7_2	120.4(2)	O2_5	C11_5	N3_5	125.8(3)
O2_2	C11_2	N1_2	127.5(2)	N1_5	C11_5	N3_5	106.87(18)
O2_2	C11_2	N3_2	125.6(2)	O3_5	C12_5	N2_5	124.3(2)
N1_2	C11_2	N3_2	106.85(15)	O3_5	C12_5	N3_5	129.6(3)
O3_2	C12_2	N2_2	124.23(18)	N3_5	C12_5	N2_5	106.16(19)
O3_2	C12_2	N3_2	129.8(2)	C3_6	O1_6	C2_6	120.8(2)
N3_2	C12_2	N2_2	105.99(15)	C6_6	N1_6	N2_6	117.5(2)
C3_3	O1_3	C2_3	120.5(2)	C11_6	N1_6	N2_6	107.22(19)
C6_3	N1_3	N2_3	117.2(2)	C11_6	N1_6	C6_6	124.7(3)
C11_3	N1_3	N2_3	107.21(18)	N2_5[1]	N2_6	N1_6	114.8(9)
C11_3	N1_3	C6_3	123.8(3)	N2_5[1]	N2_6	C12_6	104.8(7)
N1_3	N2_3	N2_4[1]	108.2(7)	C12_6	N2_6	N1_6	106.49(18)
C12_3	N2_3	N1_3	106.75(18)	C11_6	N3_6	C13_6	123.9(2)
C12_3	N2_3	N2_4[1]	130.9(6)	C12_6	N3_6	C11_6	111.34(18)
C11_3	N3_3	C13_3	123.7(2)	C12_6	N3_6	C13_6	124.8(2)
C12_3	N3_3	C11_3	111.40(19)	O1_6	C2_6	C6	109.4(4)
C12_3	N3_3	C13_3	124.9(2)	O1_6	C3_6	C4_6	120.6(2)
O1_3	C2_3	C22	105.4(4)	O1_6	C3_6	C8_6	119.0(2)

Table S14. Bond Angles for 4b$_2$.

Atom	Atom	Atom	Angle/°	Atom	Atom	Atom	Angle/°
O1_3	C3_3	C4_3	117.9(2)	C8_6	C3_6	C4_6	120.3(2)
O1_3	C3_3	C8_3	121.5(2)	C5_6	C4_6	C3_6	120.3(2)
C8_3	C3_3	C4_3	120.6(2)	C4_6	C5_6	C6_6	117.9(2)
C5_3	C4_3	C3_3	120.4(2)	C4_6	C5_6	C9_6	120.4(2)
C4_3	C5_3	C6_3	117.7(2)	C6_6	C5_6	C9_6	121.7(2)
C4_3	C5_3	C9_3	120.3(2)	C5_6	C6_6	N1_6	119.8(2)
C6_3	C5_3	C9_3	122.0(2)	C7_6	C6_6	N1_6	117.3(2)
C5_3	C6_3	N1_3	119.2(2)	C7_6	C6_6	C5_6	122.9(2)
C7_3	C6_3	N1_3	117.8(2)	C6_6	C7_6	C10_6	121.5(2)
C7_3	C6_3	C5_3	122.9(2)	C8_6	C7_6	C6_6	117.6(2)
C6_3	C7_3	C10_3	121.5(2)	C8_6	C7_6	C10_6	120.8(3)
C8_3	C7_3	C6_3	117.8(2)	C7_6	C8_6	C3_6	120.9(2)
C8_3	C7_3	C10_3	120.7(3)	O2_6	C11_6	N1_6	127.0(3)
C3_3	C8_3	C7_3	120.5(2)	O2_6	C11_6	N3_6	125.7(3)
O2_3	C11_3	N1_3	126.5(3)	N1_6	C11_6	N3_6	107.15(17)
O2_3	C11_3	N3_3	125.3(3)	O3_6	C12_6	N2_6	124.5(2)
N1_3	C11_3	N3_3	107.24(18)	O3_6	C12_6	N3_6	129.2(3)
O3_3	C12_3	N2_3	124.6(2)	N3_6	C12_6	N2_6	106.25(19)

[1]1/2-X,+Y,1-Z

Table S15. Torsion Angles for 4b$_2$.

A	B	C	D	Angle/°	A	B	C	D	Angle/°
O1_1	C3_1	C4_1	C5_1	-175.3(3)	C13_3	N3_3	C12_3	N2_3	173.5(9)
O1_1	C3_1	C8_1	C7_1	175.3(3)	O1_4	C3_4	C4_4	C5_4	173.8(6)
N1_1	O3_1	C12_1	N2_1	-17.8(5)	O1_4	C3_4	C8_4	C7_4	-178.2(9)
N1_1	O3_1	C12_1	N3_1	163.4(10)	N1_4	N2_4	C12_4	O3_4	-170.1(12)
N1_1	N2_1	C12_1	O3_1	170.3(3)	N1_4	N2_4	C12_4	N3_4	10.8(11)
N1_1	N2_1	C12_1	N3_1	-10.6(3)	N1_4	C6_4	C7_4	C8_4	172.0(8)
N1_1	C6_1	C7_1	C8_1	-176.2(2)	N1_4	C6_4	C7_4	C10_4	-7.0(8)
N1_1	C6_1	C7_1	C10_1	2.7(4)	N2_4[1]	N2_3	C12_3	O3_3	-36.0(14)
N2_1	N1_1	C6_1	C5_1	-68.2(3)	N2_4[1]	N2_3	C12_3	N3_3	145.0(10)
N2_1	N1_1	C6_1	C7_1	110.4(3)	N2_4	N1_4	C6_4	C5_4	76.4(10)
N2_1	N1_1	C11_1	O2_1	170.5(3)	N2_4	N1_4	C6_4	C7_4	-100.4(7)
N2_1	N1_1	C11_1	N3_1	-8.4(3)	N2_4	N1_4	C11_4	O2_4	-172.4(13)
N2_1[1]	N2_2	C12_2	O3_2	35.2(4)	N2_4	N1_4	C11_4	N3_4	8.9(9)
N2_1[1]	N2_2	C12_2	N3_2	-146.1(2)	C2_4	C22	C2_3	O1_3	67.8(8)
C2_1	C21	C2_2	O1_2	-54.8(4)	C2_4	O1_4	C3_4	C4_4	-163.3(6)
C2_1	O1_1	C3_1	C4_1	8.0(4)	C2_4	O1_4	C3_4	C8_4	13.2(11)

Table S15. Torsion Angles for $4b_2$.

A	B	C	D	Angle/°	A	B	C	D	Angle/°
C2_1	O1_1	C3_1	C8_1	-170.2(3)	C3_4	O1_4	C2_4	C22	170.5(6)
C3_1	O1_1	C2_1	C21	171.6(3)	C3_4	C4_4	C5_4	C6_4	3.4(11)
C3_1	C4_1	C5_1	C6_1	-0.3(4)	C3_4	C4_4	C5_4	C9_4	-178.1(8)
C3_1	C4_1	C5_1	C9_1	177.2(3)	C4_4	C3_4	C8_4	C7_4	-1.8(16)
C4_1	C3_1	C8_1	C7_1	-2.9(4)	C4_4	C5_4	C6_4	N1_4	-176.3(5)
C4_1	C5_1	C6_1	N1_1	176.1(2)	C4_4	C5_4	C6_4	C7_4	0.4(12)
C4_1	C5_1	C6_1	C7_1	-2.3(4)	C5_4	C6_4	C7_4	C8_4	-4.7(12)
C5_1	C6_1	C7_1	C8_1	2.3(4)	C5_4	C6_4	C7_4	C10_4	176.2(7)
C5_1	C6_1	C7_1	C10_1	-178.9(3)	C6_4	N1_4	N2_4	N2_3[1]	81.6(9)
C6_1	N1_1	N2_1	C12_1	162.3(2)	C6_4	N1_4	N2_4	C12_4	-158.9(7)
C6_1	N1_1	N2_1	N2_2[1]	-68.4(2)	C6_4	N1_4	C11_4	O2_4	-29.0(15)
C6_1	N1_1	C11_1	O2_1	23.1(5)	C6_4	N1_4	C11_4	N3_4	152.3(7)
C6_1	N1_1	C11_1	N3_1	-155.8(2)	C6_4	C7_4	C8_4	C3_4	5.4(16)
C6_1	C7_1	C8_1	C3_1	0.4(4)	C8_4	C3_4	C4_4	C5_4	-2.7(11)
C8_1	C3_1	C4_1	C5_1	2.9(4)	C9_4	C5_4	C6_4	N1_4	5.2(12)
C9_1	C5_1	C6_1	N1_1	-1.4(4)	C9_4	C5_4	C6_4	C7_4	-178.1(8)
C9_1	C5_1	C6_1	C7_1	-179.8(3)	C10_4	C7_4	C8_4	C3_4	-175.5(9)
C10_1	C7_1	C8_1	C3_1	-178.5(3)	C11_4	N1_4	N2_4	N2_3[1]	-131.8(8)
C11_1	N1_1	N2_1	C12_1	11.9(3)	C11_4	N1_4	N2_4	C12_4	-12.3(9)
C11_1	N1_1	N2_1	N2_2[1]	141.2(2)	C11_4	N1_4	C6_4	C5_4	-63.7(10)
C11_1	N1_1	C6_1	C5_1	76.2(3)	C11_4	N1_4	C6_4	C7_4	119.5(7)
C11_1	N1_1	C6_1	C7_1	-105.2(3)	C11_4	N3_4	C12_4	O3_4	175.3(13)
C11_1	N3_1	C12_1	O3_1	-175.2(3)	C11_4	N3_4	C12_4	N2_4	-5.6(13)
C11_1	N3_1	C12_1	N2_1	5.8(3)	C12_4	N3_4	C11_4	O2_4	179.0(13)
C12_1	N3_1	C11_1	O2_1	-177.2(3)	C12_4	N3_4	C11_4	N1_4	-2.2(12)
C12_1	N3_1	C11_1	N1_1	1.7(3)	C13_4	N3_4	C11_4	O2_4	1(2)
C13_1	N3_1	C11_1	O2_1	6.9(5)	C13_4	N3_4	C11_4	N1_4	179.7(14)
C13_1	N3_1	C11_1	N1_1	-174.2(3)	C13_4	N3_4	C12_4	O3_4	-7(2)
C13_1	N3_1	C12_1	O3_1	0.7(5)	C13_4	N3_4	C12_4	N2_4	172.5(15)
C13_1	N3_1	C12_1	N2_1	-178.3(3)	O1_5	C3_5	C4_5	C5_5	173.5(10)
O1_2	C3_2	C4_2	C5_2	-177.5(2)	O1_5	C3_5	C8_5	C7_5	-174.1(9)
O1_2	C3_2	C8_2	C7_2	174.6(3)	N1_5	N2_5	C12_5	O3_5	-164.9(12)
N1_2	N2_2	C12_2	O3_2	166.8(3)	N1_5	N2_5	C12_5	N3_5	13.2(11)
N1_2	N2_2	C12_2	N3_2	-14.5(3)	N1_5	C6_5	C7_5	C8_5	173.9(5)
N1_2	C6_2	C7_2	C8_2	-178.0(2)	N1_5	C6_5	C7_5	C10_5	-6.9(9)
N1_2	C6_2	C7_2	C10_2	1.8(4)	N2_5	N1_5	C6_5	C5_5	70.4(10)
N2_2[1]	N2_1	C12_1	O3_1	44.0(4)	N2_5	N1_5	C6_5	C7_5	-105.4(8)
N2_2[1]	N2_1	C12_1	N3_1	-137.0(2)	N2_5	N1_5	C11_5	O2_5	-170.2(12)
N2_2	N1_2	C6_2	C5_2	-69.9(3)	N2_5	N1_5	C11_5	N3_5	12.5(9)
N2_2	N1_2	C6_2	C7_2	110.3(2)	N2_5[1]	N2_6	C12_6	O3_6	-48.7(11)

Table S15. Torsion Angles for 4b$_2$.

A	B	C	D	Angle/°
N2_2	N1_2	C11_2	O2_2	168.0(3)
N2_2	N1_2	C11_2	N3_2	-11.1(3)
C2_2	C21	C2_1	O1_1	-61.9(4)
C2_2	O1_2	C3_2	C4_2	-169.2(3)
C2_2	O1_2	C3_2	C8_2	12.5(4)
C3_2	O1_2	C2_2	C21	-175.5(3)
C3_2	C4_2	C5_2	C6_2	3.3(4)
C3_2	C4_2	C5_2	C9_2	-176.1(3)
C4_2	C3_2	C8_2	C7_2	-3.6(4)
C4_2	C5_2	C6_2	N1_2	175.3(2)
C4_2	C5_2	C6_2	C7_2	-4.9(4)
C5_2	C6_2	C7_2	C8_2	2.2(4)
C5_2	C6_2	C7_2	C10_2	-178.0(3)
C6_2	N1_2	N2_2	N2_1[1]	-62.6(2)
C6_2	N1_2	N2_2	C12_2	163.2(2)
C6_2	N1_2	C11_2	O2_2	23.9(5)
C6_2	N1_2	C11_2	N3_2	-155.2(2)
C6_2	C7_2	C8_2	C3_2	2.1(4)
C8_2	C3_2	C4_2	C5_2	0.8(4)
C9_2	C5_2	C6_2	N1_2	-5.2(4)
C9_2	C5_2	C6_2	C7_2	174.6(3)
C10_2	C7_2	C8_2	C3_2	-177.7(3)
C11_2	N1_2	N2_2	N2_1[1]	150.2(2)
C11_2	N1_2	N2_2	C12_2	16.1(3)
C11_2	N1_2	C6_2	C5_2	70.8(3)
C11_2	N1_2	C6_2	C7_2	-109.0(3)
C11_2	N3_2	C12_2	O3_2	-173.3(3)
C11_2	N3_2	C12_2	N2_2	8.1(3)
C12_2	N3_2	C11_2	O2_2	-177.1(3)
C12_2	N3_2	C11_2	N1_2	2.0(4)
C13_2	N3_2	C11_2	O2_2	5.7(6)
C13_2	N3_2	C11_2	N1_2	-175.3(3)
C13_2	N3_2	C12_2	O3_2	3.9(6)
C13_2	N3_2	C12_2	N2_2	-174.6(3)
O1_3	C3_3	C4_3	C5_3	179.6(7)
O1_3	C3_3	C8_3	C7_3	179.5(9)
N1_3	N2_3	C12_3	O3_3	-169.8(8)
N1_3	N2_3	C12_3	N3_3	11.3(9)
N1_3	C6_3	C7_3	C8_3	177.3(7)
N1_3	C6_3	C7_3	C10_3	-0.8(13)
N2_5[1]	N2_6	C12_6	N3_6	133.5(8)
C2_5	C6	C2_6	O1_6	-176.0(6)
C2_5	O1_5	C3_5	C4_5	6.8(18)
C2_5	O1_5	C3_5	C8_5	177.5(10)
C3_5	O1_5	C2_5	C6	-169.7(9)
C3_5	C4_5	C5_5	C6_5	-1.7(13)
C3_5	C4_5	C5_5	C9_5	178.4(11)
C4_5	C3_5	C8_5	C7_5	-3.4(18)
C4_5	C5_5	C6_5	N1_5	-174.3(5)
C4_5	C5_5	C6_5	C7_5	1.3(12)
C5_5	C6_5	C7_5	C8_5	-1.8(11)
C5_5	C6_5	C7_5	C10_5	177.4(7)
C6_5	N1_5	N2_5	C12_5	-164.7(6)
C6_5	N1_5	N2_5	N2_6[1]	48.4(10)
C6_5	N1_5	C11_5	O2_5	-24.9(13)
C6_5	N1_5	C11_5	N3_5	157.8(7)
C6_5	C7_5	C8_5	C3_5	2.8(12)
C8_5	C3_5	C4_5	C5_5	2.8(18)
C9_5	C5_5	C6_5	N1_5	5.6(12)
C9_5	C5_5	C6_5	C7_5	-178.8(8)
C10_5	C7_5	C8_5	C3_5	-176.4(10)
C11_5	N1_5	N2_5	C12_5	-16.1(9)
C11_5	N1_5	N2_5	N2_6[1]	-162.9(8)
C11_5	N1_5	C6_5	C5_5	-71.7(9)
C11_5	N1_5	C6_5	C7_5	112.5(7)
C11_5	N3_5	C12_5	O3_5	172.1(13)
C11_5	N3_5	C12_5	N2_5	-5.9(13)
C12_5	N3_5	C11_5	O2_5	178.3(12)
C12_5	N3_5	C11_5	N1_5	-4.3(12)
C13_5	N3_5	C11_5	O2_5	-4(2)
C13_5	N3_5	C11_5	N1_5	173.0(14)
C13_5	N3_5	C12_5	O3_5	-5(2)
C13_5	N3_5	C12_5	N2_5	176.8(15)
O1_6	C3_6	C4_6	C5_6	179.0(7)
O1_6	C3_6	C8_6	C7_6	179.9(7)
N1_6	N2_6	C12_6	O3_6	-170.7(7)
N1_6	N2_6	C12_6	N3_6	11.5(9)
N1_6	C6_6	C7_6	C8_6	-178.9(6)
N1_6	C6_6	C7_6	C10_6	-1.0(12)
N2_6[1]	N2_5	C12_5	O3_5	-32.8(19)

Table S15. Torsion Angles for 4b$_2$.

A	B	C	D	Angle/°	A	B	C	D	Angle/°
N2_3	N1_3	C6_3	C5_3	69.3(8)	N2_6[1]	N2_5	C12_5	N3_5	145.4(15)
N2_3	N1_3	C6_3	C7_3	-111.9(8)	N2_6	N1_6	C6_6	C5_6	73.1(8)
N2_3	N1_3	C11_3	O2_3	-163.8(17)	N2_6	N1_6	C6_6	C7_6	-108.4(8)
N2_3	N1_3	C11_3	N3_3	5.4(8)	N2_6	N1_6	C11_6	O2_6	-175.5(16)
N2_3[1]	N2_4	C12_4	O3_4	-38.1(14)	N2_6	N1_6	C11_6	N3_6	8.8(7)
N2_3[1]	N2_4	C12_4	N3_4	142.8(12)	C2_6	C6	C2_5	O1_5	72.1(9)
C2_3	C22	C2_4	O1_4	-161.0(5)	C2_6	O1_6	C3_6	C4_6	11.7(9)
C2_3	O1_3	C3_3	C4_3	178.7(7)	C2_6	O1_6	C3_6	C8_6	-167.1(5)
C2_3	O1_3	C3_3	C8_3	0.2(14)	C3_6	O1_6	C2_6	C6	176.2(6)
C3_3	O1_3	C2_3	C22	-175.9(7)	C3_6	C4_6	C5_6	C6_6	1.9(12)
C3_3	C4_3	C5_3	C6_3	-0.2(10)	C3_6	C4_6	C5_6	C9_6	179.7(7)
C3_3	C4_3	C5_3	C9_3	177.7(7)	C4_6	C3_6	C8_6	C7_6	1.1(10)
C4_3	C3_3	C8_3	C7_3	1.0(15)	C4_6	C5_6	C6_6	N1_6	177.8(6)
C4_3	C5_3	C6_3	N1_3	-178.2(5)	C4_6	C5_6	C6_6	C7_6	-0.6(12)
C4_3	C5_3	C6_3	C7_3	3.2(11)	C5_6	C6_6	C7_6	C8_6	-0.5(13)
C5_3	C6_3	C7_3	C8_3	-4.0(14)	C5_6	C6_6	C7_6	C10_6	177.4(9)
C5_3	C6_3	C7_3	C10_3	177.9(10)	C6_6	N1_6	N2_6	N2_5[1]	85.4(7)
C6_3	N1_3	N2_3	C12_3	-154.8(5)	C6_6	N1_6	N2_6	C12_6	-159.1(5)
C6_3	N1_3	N2_3	N2_4[1]	60.3(7)	C6_6	N1_6	C11_6	O2_6	-32.2(17)
C6_3	N1_3	C11_3	O2_3	-22.4(18)	C6_6	N1_6	C11_6	N3_6	152.2(5)
C6_3	N1_3	C11_3	N3_3	146.9(6)	C6_6	C7_6	C8_6	C3_6	0.2(12)
C6_3	C7_3	C8_3	C3_3	1.8(14)	C8_6	C3_6	C4_6	C5_6	-2.2(12)
C8_3	C3_3	C4_3	C5_3	-1.9(13)	C9_6	C5_6	C6_6	N1_6	0.0(9)
C9_3	C5_3	C6_3	N1_3	4.0(9)	C9_6	C5_6	C6_6	C7_6	-178.3(8)
C9_3	C5_3	C6_3	C7_3	-174.6(8)	C10_6	C7_6	C8_6	C3_6	-177.7(8)
C10_3	C7_3	C8_3	C3_3	180.0(10)	C11_6	N1_6	N2_6	N2_5[1]	-128.1(6)
C11_3	N1_3	N2_3	C12_3	-10.4(8)	C11_6	N1_6	N2_6	C12_6	-12.7(8)
C11_3	N1_3	N2_3	N2_4[1]	-155.3(7)	C11_6	N1_6	C6_6	C5_6	-66.9(7)
C11_3	N1_3	C6_3	C5_3	-68.7(8)	C11_6	N1_6	C6_6	C7_6	111.5(8)
C11_3	N1_3	C6_3	C7_3	110.1(8)	C11_6	N3_6	C12_6	O3_6	176.0(8)
C11_3	N3_3	C12_3	O3_3	172.8(9)	C11_6	N3_6	C12_6	N2_6	-6.4(11)
C11_3	N3_3	C12_3	N2_3	-8.3(11)	C12_6	N3_6	C11_6	O2_6	-177.4(16)
C12_3	N3_3	C11_3	O2_3	171.2(17)	C12_6	N3_6	C11_6	N1_6	-1.6(10)
C12_3	N3_3	C11_3	N1_3	1.8(11)	C13_6	N3_6	C11_6	O2_6	2(2)
C13_3	N3_3	C11_3	O2_3	-11(2)	C13_6	N3_6	C11_6	N1_6	177.9(8)
C13_3	N3_3	C11_3	N1_3	-180.0(8)	C13_6	N3_6	C12_6	O3_6	-3.6(16)
C13_3	N3_3	C12_3	O3_3	-5.4(16)	C13_6	N3_6	C12_6	N2_6	174.1(9)

[1] 1/2-X,+Y,1-Z

Table S16. Hydrogen Atom Coordinates (Å×10[4]) and Isotropic Displacement Parameters (Å[2]×10[3]) for 4b₂.

Atom	*x*	*y*	*z*	U(eq)
H21A	3400.18	-77.49	4167.07	105
H21B	2875.71	-217.21	4364.08	105
H22A	2791.34	7225.98	5273.78	72
H22B	3230.34	7526.86	5656.88	72
H6A	3406.1	6942.12	6054.15	78
H6B	3138.54	7476.96	5635.99	78
H2A_1	3110.84	564.49	5054.1	92
H2B_1	3475.93	-145.68	5032.91	92
H4_1	3650.32	520.43	5747.44	76
H8_1	4409.33	1850.12	4881.39	77
H9A_1	3665.24	1409.55	6711.01	102
H9B_1	4223.54	1174.01	6874.96	102
H9C_1	3844.05	559.67	6629.92	102
H10A_1	4852.77	3141.27	5781.25	102
H10B_1	4832.42	2948.22	5207.25	102
H10C_1	5232.34	2545.86	5589.37	102
H13A_1	5091.35	2308.99	7967.51	112
H13B_1	5390.64	3000.66	7763.16	112
H13C_1	5532.15	2148.27	7636.48	112
H2A_2	3210.45	1190.4	4008.49	90
H2B_2	2778.33	676.26	3741.6	90
H4_2	2082.92	2080.7	4798.42	77
H8_2	2517.94	1733.15	3441.71	74
H9A_2	1526.54	3037.98	4954.56	106
H9B_2	1168.03	3354.84	4501.86	106
H9C_2	1670.91	3788.01	4673	106
H10A_2	1637.45	2732.29	2780.34	103
H10B_2	2186.97	2430.61	2753.3	103
H10C_2	2093.52	3298.77	2884.09	103
H13A_2	925.84	5622.37	3939.72	140
H13B_2	649.05	5590.1	3395.68	140
H13C_2	1232.14	5716.27	3473.24	140
H2A_3	3593.73	6981.58	5000.81	80(20)
H2B_3	3705.27	6418.34	5461.62	38(15)
H4_3	2768.69	4966.82	4488.03	29(10)
H8_3	4097.72	5941.39	4852.16	72
H9A_3	3080.65	3344.51	4128.1	89
H9B_3	3108.36	3742.5	3605.27	54(15)
H9C_3	2671.04	3949.25	3931.19	81

Table S16. Hydrogen Atom Coordinates (Å×10⁴) and Isotropic Displacement Parameters (Å²×10³) for 4b₂.

Atom	*x*	*y*	*z*	U(eq)
H10A_3	4832.35	4618.2	4586.87	98
H10B_3	4812.19	5516.93	4512.3	170(50)
H10C_3	4750.56	4967.49	4044.41	46(15)
H13A_3	4444.15	1837.63	3410.75	113
H13B_3	3865.7	1791.26	3246.48	113
H13C_3	4063.75	1752.97	3817.18	113
H2A_4	2827.23	6053.95	5786.9	75
H2B_4	3132.05	6563.08	6199.14	75
H4_4	1560.5	7217.96	6159.41	69
H8_4	2649.78	5735.58	6586.17	61
H9A_4	795.79	7168.6	6514.25	92
H9B_4	700.29	6528.62	6908.56	92
H9C_4	615.29	6343.84	6332.95	92
H10A_4	2263.68	5049.96	7555.95	92
H10B_4	2519.61	4697.74	7105.22	92
H10C_4	1968.3	4462.77	7190.87	92
H13A_4	278.9	5475.32	8110.93	114
H13B_4	30.38	4694.78	7920.43	114
H13C_4	-142	5474.88	7659.47	114
H2A_5	2616.81	7442.26	6253.43	101
H2B_5	2358.62	6906.72	5825.67	101
H4_5	1813.49	6813.95	6294.56	74
H8_5	2707.55	5226.98	6974.42	76
H9A_5	872.25	6721.07	6885.33	92
H9B_5	711.13	6026.28	6529.15	92
H9C_5	956.08	6762.95	6314.4	92
H10A_5	1930.99	4572.63	7775.52	98
H10B_5	2433.21	4380.15	7545.21	98
H10C_5	1926.21	4012.82	7314.41	98
H13A_5	168.7	5099.8	8146.44	115
H13B_5	-183.99	4566.89	7792.99	115
H13C_5	-194.32	5464.96	7714.16	115
H2A_6	3081.29	5845.37	5597.34	69
H2B_6	2850.57	6399.53	5169.67	69
H4_6	2999.08	5337.21	4793.59	60
H8_6	4412.1	6062.5	4928.08	63
H9A_6	3121.32	3701.44	4277.73	88
H9B_6	3042.94	4212.66	3792.63	88
H9C_6	2726.56	4374.12	4244.42	88

Table S16. Hydrogen Atom Coordinates (Å×10[4]) and Isotropic Displacement Parameters (Å[2]×10[3]) for 4b$_2$.

Atom	*x*	*y*	*z*	U(eq)
H10A_6	4928.39	4566.17	4418.13	92
H10B_6	5029.22	5440.53	4545	92
H10C_6	4841.75	5192.18	3994.86	92
H13A_6	4305.1	1974.35	3600.27	105
H13B_6	3854.68	2041.83	3180.71	105
H13C_6	3751.07	1957.67	3744.58	105

Table S17. Atomic Occupancy for 4b$_2$.

Atom	*Occupancy*	Atom	*Occupancy*	Atom	*Occupancy*
C22	0.5	H22A	0.5	H22B	0.5
C6	0.5	H6A	0.5	H6B	0.5
O1_3	0.5	O2_3	0.5	O3_3	0.5
N1_3	0.5	N2_3	0.5	N3_3	0.5
C2_3	0.5	H2A_3	0.5	H2B_3	0.5
C3_3	0.5	C4_3	0.5	H4_3	0.5
C5_3	0.5	C6_3	0.5	C7_3	0.5
C8_3	0.5	H8_3	0.5	C9_3	0.5
H9A_3	0.5	H9B_3	0.5	H9C_3	0.5
C10_3	0.5	H10A_3	0.5	H10B_3	0.5
H10C_3	0.5	C11_3	0.5	C12_3	0.5
C13_3	0.5	H13A_3	0.5	H13B_3	0.5
H13C_3	0.5	O1_4	0.5	O2_4	0.5
O3_4	0.5	N1_4	0.5	N2_4	0.5
N3_4	0.5	C2_4	0.5	H2A_4	0.5
H2B_4	0.5	C3_4	0.5	C4_4	0.5
H4_4	0.5	C5_4	0.5	C6_4	0.5
C7_4	0.5	C8_4	0.5	H8_4	0.5
C9_4	0.5	H9A_4	0.5	H9B_4	0.5
H9C_4	0.5	C10_4	0.5	H10A_4	0.5
H10B_4	0.5	H10C_4	0.5	C11_4	0.5
C12_4	0.5	C13_4	0.5	H13A_4	0.5
H13B_4	0.5	H13C_4	0.5	O1_5	0.5
O2_5	0.5	O3_5	0.5	N1_5	0.5
N2_5	0.5	N3_5	0.5	C2_5	0.5
H2A_5	0.5	H2B_5	0.5	C3_5	0.5
C4_5	0.5	H4_5	0.5	C5_5	0.5
C6_5	0.5	C7_5	0.5	C8_5	0.5

Table S17. Atomic Occupancy for 4b₂.

Atom	*Occupancy*	Atom	*Occupancy*	Atom	*Occupancy*
H8_5	0.5	C9_5	0.5	H9A_5	0.5
H9B_5	0.5	H9C_5	0.5	C10_5	0.5
H10A_5	0.5	H10B_5	0.5	H10C_5	0.5
C11_5	0.5	C12_5	0.5	C13_5	0.5
H13A_5	0.5	H13B_5	0.5	H13C_5	0.5
O1_6	0.5	O2_6	0.5	O3_6	0.5
N1_6	0.5	N2_6	0.5	N3_6	0.5
C2_6	0.5	H2A_6	0.5	H2B_6	0.5
C3_6	0.5	C4_6	0.5	H4_6	0.5
C5_6	0.5	C6_6	0.5	C7_6	0.5
C8_6	0.5	H8_6	0.5	C9_6	0.5
H9A_6	0.5	H9B_6	0.5	H9C_6	0.5
C10_6	0.5	H10A_6	0.5	H10B_6	0.5
H10C_6	0.5	C11_6	0.5	C12_6	0.5
C13_6	0.5	H13A_6	0.5	H13B_6	0.5
H13C_6	0.5				

Table S18. Solvent masks information for 4b₂.

Number	X	Y	Z	Volume	Electron count	Content
1	-0.587	-0.515	-0.150	3899.3	1227.4	32 C1,64 H1,16 Cl3,16 O1
2	0.250	0.111	0.000	9.0	0.0	?
3	0.250	0.389	0.500	9.0	0.0	?
4	0.750	0.611	0.500	9.0	0.0	?
5	0.750	0.889	0.000	9.0	0.0	?

Experimental

Single crystals were recrystallized from a solution of Compound **4b₂** (GB-2021-01) dissolved in a mixture of dichlomethane and methanol. A suitable crystal with dimensions 0.37 × 0.30 × 0.21 mm³ was selected and mounted on a loop with paratone oil on a XtaLAB Synergy, Dualflex, HyPix diffractometer. The crystal was kept at 109 K during data collection. Using *Olex2* [1], the structure was solved with the *olex2.solve* [2] structure solution program and refined with *SHELXL* [3] via full matrix least squares minimization on F^2.

Crystal structure determination of 4b₂

Crystal Data for $C_{53}H_{59}Cl_9N_{12}O_{12}$ (M =1375.17 g/mol): monoclinic, space group I2/a (no. 15), a = 27.2958(4) Å, b = 17.6590(5) Å, c = 27.3191(6) Å, β = 95.6702(16)°, V = 13103.8(5) Å^3, Z = 8, T = 109(2) K, μ(Cu Kα) = 4.071 mm^{-1}, $Dcalc$ = 1.394 g/cm^3, 76750 reflections measured (6.502° ≤ 2Θ ≤ 130.176°), 11103 unique (R_{int} = 0.0541, R_{sigma} = 0.0334) which were used in all calculations. The final R_1 was 0.0707 (I > 2σ(I)) and wR_2 was 0.2169 (all data).

Refinement model description

Number of restraints - 1592, number of constraints - unknown.

Details:

1. Restrained distances
 C21-C2_1 ≈ C21-C2_2
 with sigma of 0.002
 C22-C2_3 ≈ C22-C2_4
 with sigma of 0.002
2. Uiso/Uaniso restraints and constraints
Uanis(O3_4) = Uanis(O3_5)
Uanis(N1_4) = Uanis(N1_5)
Uanis(N2_4) = Uanis(N2_5)
Uanis(C12_4) = Uanis(C12_5)
Uanis(O3_3) = Uanis(O3_6)
Uanis(N1_3) = Uanis(N1_6)
Uanis(N3_6) = Uanis(N3_3)
Uanis(C13_6) = Uanis(C11_3)
3. Rigid body (RIGU) restrains
 with sigma for 1-2 distances of 0.002 and sigma for 1-3 distances of 0.004
4. Same fragment restrains
5. Others
 Fixed Sof: C22(0.5) H22A(0.5) H22B(0.5) C6(0.5) H6A(0.5) H6B(0.5) O1(0.5)
 O2(0.5) O3(0.5) N1(0.5) N2(0.5) N3(0.5) C2(0.5) H2A(0.5) H2B(0.5) C3(0.5)
 C4(0.5) H4(0.5) C5(0.5) C6(0.5) C7(0.5) C8(0.5) H8(0.5) C9(0.5) H9A(0.5)
 H9B(0.5) H9C(0.5) C10(0.5) H10A(0.5) H10B(0.5) H10C(0.5) C11(0.5) C12(0.5)
 C13(0.5) H13A(0.5) H13B(0.5) H13C(0.5) O1(0.5) O2(0.5) O3(0.5) N1(0.5) N2(0.5)
 N3(0.5) C2(0.5) H2A(0.5) H2B(0.5) C3(0.5) C4(0.5) H4(0.5) C5(0.5) C6(0.5)
 C7(0.5) C8(0.5) H8(0.5) C9(0.5) H9A(0.5) H9B(0.5) H9C(0.5) C10(0.5) H10A(0.5)
 H10B(0.5) H10C(0.5) C11(0.5) C12(0.5) C13(0.5) H13A(0.5) H13B(0.5) H13C(0.5)
 O1(0.5) O2(0.5) O3(0.5) N1(0.5) N2(0.5) N3(0.5) C2(0.5) H2A(0.5) H2B(0.5)
 C3(0.5) C4(0.5) H4(0.5) C5(0.5) C6(0.5) C7(0.5) C8(0.5) H8(0.5) C9(0.5)
 H9A(0.5) H9B(0.5) H9C(0.5) C10(0.5) H10A(0.5) H10B(0.5) H10C(0.5) C11(0.5)
 C12(0.5) C13(0.5) H13A(0.5) H13B(0.5) H13C(0.5) O1(0.5) O2(0.5) O3(0.5)
 N1(0.5) N2(0.5) N3(0.5) C2(0.5) H2A(0.5) H2B(0.5) C3(0.5) C4(0.5) H4(0.5)
 C5(0.5) C6(0.5) C7(0.5) C8(0.5) H8(0.5) C9(0.5) H9A(0.5) H9B(0.5) H9C(0.5)
 C10(0.5) H10A(0.5) H10B(0.5) H10C(0.5) C11(0.5) C12(0.5) C13(0.5) H13A(0.5)
 H13B(0.5) H13C(0.5)
6.a Secondary CH2 refined with riding coordinates:
 C21(H21A,H21B), C22(H22A,H22B), C6(H6A,H6B), C2(H2A,H2B), C2(H2A,H2B), C2(H2A,
 H2B), C2(H2A,H2B), C2(H2A,H2B), C2(H2A,H2B)
6.b Aromatic/amide H refined with riding coordinates:
 C4(H4), C8(H8), C4(H4), C8(H8), C4(H4), C8(H8), C4(H4), C8(H8), C4(H4),
 C8(H8), C4(H4), C8(H8)
6.c Idealised Me refined as rotating group:
 C9(H9A,H9B,H9C), C10(H10A,H10B,H10C), C13(H13A,H13B,H13C), C9(H9A,H9B,H9C),
 C10(H10A,H10B,H10C), C13(H13A,H13B,H13C), C9(H9A,H9B,H9C), C10(H10A,H10B,H10C),
 C13(H13A,H13B,H13C), C9(H9A,H9B,H9C), C10(H10A,H10B,H10C), C13(H13A,H13B,
 H13C), C9(H9A,H9B,H9C), C10(H10A,H10B,H10C), C13(H13A,H13B,H13C), C9(H9A,H9B,
 H9C), C10(H10A,H10B,H10C), C13(H13A,H13B,H13C)

This report has been created with Olex2, compiled on 2023.02.24 svn.rf166f9f3 for OlexSys. Please let us know if there are any errors or if you would like to have additional features.

Crystal Structure Data for Dimer $4c_2$

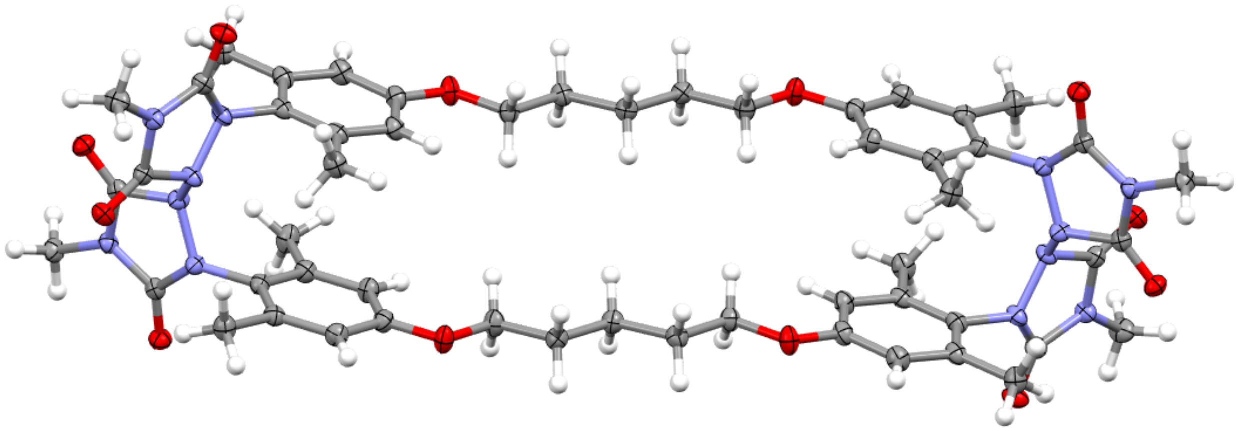

*elipsoids represent 50% probability level

Table S19. Crystal data and structure refinement for $4c_2$.

Identification code	GB-dimer-4-21-22
Empirical formula	$C_{63}H_{82}N_{12}O_{21}$
Formula weight	1343.40
Temperature/K	100.0(9)
Crystal system	monoclinic
Space group	$P2_1/c$
a/Å	8.92534(8)
b/Å	14.21670(12)
c/Å	51.8332(4)
α/°	90
β/°	90.4433(8)
γ/°	90
Volume/Å^3	6576.87(9)
Z	4
ρ_{calc}g/cm^3	1.357
μ/mm^{-1}	0.862
F(000)	2848.0
Crystal size/mm^3	0.27 × 0.21 × 0.1
Radiation	Cu Kα (λ = 1.54184)
2Θ range for data collection/°	6.446 to 146.38
Index ranges	$-9 \le h \le 11$, $-17 \le k \le 15$, $-56 \le l \le 63$
Reflections collected	58388
Independent reflections	12479 [R_{int} = 0.0609, R_{sigma} = 0.0455]
Data/restraints/parameters	12479/30/899
Goodness-of-fit on F^2	1.020

Final R indexes [I>=2σ (I)] R_1 = 0.0448, wR_2 = 0.1092
Final R indexes [all data] R_1 = 0.0554, wR_2 = 0.1150
Largest diff. peak/hole / e Å^{-3} 0.24/-0.25

Table S20. Fractional Atomic Coordinates (×10^4) and Equivalent Isotropic Displacement Parameters (Å^2×10^3) for 4c$_2$. U_{eq} is defined as 1/3 of the trace of the orthogonalised U_{IJ} tensor.

Atom	*x*	*y*	*z*	U(eq)
O1_4	3195.7(15)	4338.6(9)	5645.5(2)	26.8(3)
O2_4	2987.4(14)	4699.7(9)	4238.9(2)	26.5(3)
O3_4	-16.8(13)	5719.7(8)	7175.3(2)	23.5(3)
O4_4	3998.7(13)	6763.8(8)	6709.9(2)	25.5(3)
O5_4	2260.2(13)	7240.7(8)	3208.0(2)	23.5(3)
O6_4	-2094.9(13)	6082.6(8)	2855.2(2)	23.0(3)
N1_4	2368.7(16)	5511.2(10)	6657.7(3)	19.8(3)
N2_4	967.4(16)	5284.5(9)	6777.8(3)	19.1(3)
N3_4	2058.6(16)	6442.6(10)	6994.4(3)	20.2(3)
N4_4	831.4(15)	5915.8(10)	3289.6(3)	19.2(3)
N5_4	-672.4(15)	5672.6(9)	3217.1(3)	18.9(3)
N6_4	29.7(15)	6902.5(10)	2989.3(3)	19.2(3)
C1_4	2548(2)	4840.0(12)	5433.2(3)	22.3(4)
C2_4	3067(2)	4386.8(12)	5185.7(3)	22.4(4)
C3_4	2544(2)	4950.3(12)	4952.9(3)	23.1(4)
C4_4	3041(2)	4531.5(12)	4696.3(3)	22.7(4)
C5_4	2504(2)	5129.8(13)	4473.3(3)	25.5(4)
C6_4	904.2(19)	5811.0(11)	7009.1(3)	19.1(3)
C7_4	2934.8(19)	6293.0(12)	6776.5(3)	20.3(3)
C8_4	2319(2)	7182.6(13)	7184.5(3)	27.4(4)
C9_4	2581.0(19)	5251.3(12)	6392.6(3)	20.3(3)
C10_4	3518(2)	4483.4(12)	6342.3(3)	21.9(4)
C11_4	3695(2)	4215.6(12)	6086.9(3)	24.5(4)
C12_4	2029(2)	5449.2(12)	5942.9(3)	23.2(4)
C13_4	1845.7(19)	5746.2(12)	6197.3(3)	22.0(4)
C14_4	4306(2)	3955.1(13)	6555.0(3)	25.6(4)
C15_4	-1038.1(19)	6215.0(12)	2996.8(3)	19.4(3)
C16_4	1180.8(18)	6745.5(11)	3167.5(3)	18.6(3)
C17_4	-5(2)	7681.5(12)	2806.4(3)	23.6(4)
C18_4	1379.1(19)	5640.1(12)	3537.8(3)	19.6(3)
C19_4	960.0(19)	6140.9(12)	3757.2(3)	22.2(4)

Table S20. Fractional Atomic Coordinates (×10^4) and Equivalent Isotropic Displacement Parameters (Å^2×10^3) for 4c$_2$. U$_{eq}$ is defined as 1/3 of the trace of the orthogonalised U$_{IJ}$ tensor.

Atom	*x*	*y*	*z*	U(eq)
C20_4	1504(2)	5844.7(12)	3997.3(3)	23.4(4)
C21_4	2442(2)	5065.3(12)	4013.1(3)	22.4(4)
C22_4	2878(2)	4592.8(12)	3790.5(3)	22.6(4)
C23_4	2337.3(19)	4862.8(12)	3551.0(3)	20.9(4)
C24_4	-42(2)	6991.4(13)	3736.9(3)	27.7(4)
C25_4	2747(2)	4316.6(13)	3312.5(3)	26.1(4)
C26_4	887(2)	6591.4(13)	6256.7(3)	26.9(4)
C27_4	2951(2)	4682.8(12)	5888.9(3)	23.0(4)
O1_5	8947.5(14)	4777.1(9)	681.7(2)	24.7(3)
O2_5	7209.1(15)	4356.0(8)	-692.3(2)	26.1(3)
O3_5	5001.1(13)	6040.9(8)	2145.5(2)	22.8(3)
O4_5	9340.4(13)	7122.2(8)	1782.1(2)	23.7(3)
O5_5	7040.4(13)	6823.0(8)	-1739.7(2)	23.3(3)
O6_5	2851.1(13)	5723.4(8)	-2158.5(2)	22.3(3)
N1_5	7783.6(15)	5857.4(10)	1689.6(3)	18.9(3)
N2_5	6292.0(15)	5630.4(9)	1773.1(3)	18.3(3)
N3_5	7183.9(16)	6788.1(10)	2014.4(3)	20.3(3)
N4_5	5456.6(15)	5558.5(10)	-1668.4(3)	18.9(3)
N5_5	4001.9(15)	5328.9(9)	-1768.9(3)	18.3(3)
N6_5	4936.8(15)	6511.0(9)	-1993.7(3)	18.7(3)
C1_5	8119(2)	5186.9(13)	473.1(3)	22.8(4)
C2_5	8324(2)	4578.6(12)	237.0(3)	21.6(4)
C3_5	7486.8(19)	4994.1(12)	5.8(3)	21.8(4)
C4_5	7692(2)	4430.5(12)	-241.7(3)	21.9(4)
C5_5	6859(2)	4875.0(12)	-464.0(3)	23.6(4)
C6_5	6040.3(19)	6147.4(12)	2001.7(3)	19.4(3)
C7_5	8237.8(19)	6647.9(11)	1821.9(3)	18.8(3)
C8_5	7287(2)	7513.9(13)	2213.3(3)	27.5(4)
C9_5	8151.6(19)	5645.9(11)	1426.9(3)	18.8(3)
C10_5	9087.2(19)	4875.6(12)	1380.6(3)	20.1(3)
C11_5	9357.0(19)	4635.6(12)	1125.7(3)	21.0(4)
C12_5	7803(2)	5910.3(12)	974.1(3)	21.9(4)
C13_5	7527.4(19)	6177.3(12)	1227.6(3)	20.7(3)
C14_5	9750(2)	4303.4(12)	1596.9(3)	24.0(4)
C15_5	3821.0(19)	5854.4(12)	-1999.2(3)	18.8(3)
C16_5	5945.2(19)	6351.4(11)	-1792.9(3)	19.2(3)
C17_5	5065(2)	7267.1(12)	-2182.2(3)	23.2(4)
C18_5	5870.1(19)	5276.6(12)	-1412.6(3)	19.8(3)

Table S20. Fractional Atomic Coordinates (×10[4]) and Equivalent Isotropic Displacement Parameters (Å[2]×10[3]) for 4c$_2$. U$_{eq}$ is defined as 1/3 of the trace of the orthogonalised U$_{IJ}$ tensor.

Atom	*x*	*y*	*z*	U(eq)
C19_5	5321.7(19)	5766.2(12)	-1199.1(3)	21.3(4)
C20_5	5757(2)	5468.0(12)	-953.3(3)	22.1(4)
C21_5	6716(2)	4703.5(12)	-924.6(3)	22.2(4)
C22_5	7247(2)	4229.8(12)	-1140.0(3)	23.0(4)
C23_5	6828.1(19)	4503.5(12)	-1387.2(3)	20.8(4)
C24_5	4303(2)	6600.3(13)	-1231.8(3)	26.3(4)
C25_5	7402(2)	3969.6(13)	-1616.7(3)	25.3(4)
C26_5	6571(2)	7023.5(13)	1285.0(3)	27.1(4)
C27_5	8689.1(19)	5124.5(12)	923.9(3)	21.0(4)
O1S_1	600.6(17)	6816.1(11)	4737.7(3)	46.0(4)
O2S_1	-1136.9(15)	7928.4(9)	4658.3(3)	32.7(3)
O3S_1	-492.5(15)	7660.9(10)	5051.6(3)	34.7(3)
C1S_1	-253(2)	7409.7(13)	4808.5(4)	30.0(4)
C2S_1	-986(2)	7741.6(15)	4385.4(4)	37.1(5)
C3S_1	357(2)	7142.3(15)	5241.8(4)	39.9(5)
O1S_2	4112.2(16)	1863.5(10)	4710.7(3)	43.6(4)
O2S_2	5824.1(15)	2909.7(9)	4570.8(3)	34.7(3)
O3S_2	5651.6(15)	2671.9(10)	4978.4(3)	35.7(3)
C1S_2	5089(2)	2424.6(13)	4750.2(4)	31.2(4)
C2S_2	5307(2)	2750.0(15)	4309.3(4)	39.1(5)
C3S_2	5026(3)	2178.4(15)	5196.4(4)	42.2(5)
O1S_3	2673(3)	4410.8(18)	2393.2(5)	27.7(4)
O2S_3	3481(3)	5514.3(18)	2675.8(5)	25.0(3)
O3S_3	1381(3)	5746.4(18)	2481.1(5)	25.0(3)
C1S_3	2550(50)	5160(30)	2498(12)	22.3(13)
C2S_3	4864(17)	5005(13)	2711(4)	29.2(14)
C3S_3	177(19)	5413(14)	2318(4)	28.0(15)
O1_3	2481(3)	5997.5(16)	2604.3(4)	27.7(4)
O2_3	3802(3)	4661.3(16)	2537.2(4)	25.0(3)
O3_3	1702(3)	4831.4(16)	2337.8(4)	25.0(3)
C1_3	2630(40)	5230(30)	2509(10)	22.3(13)
C2_3	5023(15)	5042(11)	2685(3)	29.2(14)
C3_3	328(16)	5345(12)	2293(3)	28.0(15)

Table S21. Anisotropic Displacement Parameters (Å^2×10^3) for 4c$_2$. The Anisotropic displacement factor exponent takes the form: $-2\pi^2[h^2a^{*2}U_{11}+2hka^*b^*U_{12}+…]$.

Atom	U_{11}	U_{22}	U_{33}	U_{23}	U_{13}	U_{12}
O1_4	39.7(8)	22.6(6)	18.1(6)	-0.2(5)	2.4(5)	4.8(6)
O2_4	34.5(7)	26.6(7)	18.3(6)	0.2(5)	-1.8(5)	9.7(6)
O3_4	24.7(6)	21.9(6)	24.1(6)	0.3(5)	5.0(5)	0.1(5)
O4_4	23.7(6)	19.2(6)	33.7(7)	-1.2(5)	5.4(5)	-3.0(5)
O5_4	23.1(6)	21.4(6)	26.0(6)	1.9(5)	-1.7(5)	-4.6(5)
O6_4	22.8(6)	21.0(6)	24.9(6)	-0.6(5)	-3.7(5)	-1.1(5)
N1_4	21.7(7)	16.9(7)	21.0(7)	-0.7(6)	5.3(6)	-2.8(6)
N2_4	21.8(7)	14.1(7)	21.5(7)	-0.1(5)	4.2(6)	-3.1(6)
N3_4	22.5(7)	17.3(7)	20.7(7)	-3.1(6)	2.0(5)	-2.0(6)
N4_4	20.0(7)	16.7(7)	20.9(7)	2.1(6)	-2.2(5)	-2.0(6)
N5_4	20.8(7)	12.9(7)	23.0(7)	1.6(5)	-1.8(6)	-2.0(6)
N6_4	21.7(7)	16.3(7)	19.5(7)	1.7(5)	0.1(5)	-1.1(6)
C1_4	27.0(9)	19.0(9)	21.1(8)	1.5(7)	0.1(7)	0.3(7)
C2_4	25.8(9)	19.0(8)	22.3(9)	-0.6(7)	1.3(7)	-1.2(7)
C3_4	25.2(9)	21.5(9)	22.6(8)	-0.6(7)	-0.1(7)	1.3(7)
C4_4	23.2(9)	22.3(9)	22.6(9)	-0.3(7)	-1.3(7)	0.3(7)
C5_4	31.9(10)	23.5(9)	21.2(9)	-2.0(7)	1.5(7)	6.0(8)
C6_4	21.9(9)	15.5(8)	19.8(8)	0.6(6)	-1.6(7)	0.7(7)
C7_4	21.3(9)	15.8(8)	23.7(8)	1.1(7)	1.5(7)	2.3(7)
C8_4	29.2(10)	23.6(9)	29.5(9)	-9.2(8)	1.8(7)	-3.4(8)
C9_4	23.6(9)	16.4(8)	21.0(8)	-1.1(7)	3.7(7)	-3.2(7)
C10_4	25.7(9)	17.5(8)	22.6(8)	0.9(7)	3.2(7)	-3.2(7)
C11_4	29.7(10)	18.2(9)	25.6(9)	-0.1(7)	3.8(7)	2.4(7)
C12_4	27.4(9)	20.0(9)	22.2(8)	4.5(7)	2.2(7)	0.5(7)
C13_4	23.7(9)	17.1(8)	25.1(9)	0.9(7)	4.3(7)	-2.7(7)
C14_4	32.8(10)	20.9(9)	23.2(9)	-0.4(7)	1.6(7)	4.6(8)
C15_4	23.0(9)	16.1(8)	19.3(8)	-1.5(6)	2.0(7)	2.4(7)
C16_4	21.8(9)	15.6(8)	18.2(8)	-1.2(6)	1.6(6)	0.5(7)
C17_4	28.9(10)	17.6(9)	24.2(9)	6.3(7)	-1.9(7)	-2.4(7)
C18_4	21.7(9)	17.7(8)	19.4(8)	1.4(6)	-1.8(6)	-2.2(7)
C19_4	24.6(9)	17.8(9)	24.3(9)	0.5(7)	-0.9(7)	-0.1(7)
C20_4	28.3(9)	20.4(9)	21.6(8)	-2.6(7)	-0.5(7)	2.6(7)
C21_4	24.5(9)	20.1(9)	22.6(8)	2.1(7)	-1.4(7)	-0.1(7)
C22_4	24.2(9)	18.0(9)	25.5(9)	0.7(7)	0.2(7)	2.7(7)
C23_4	21.5(9)	18.0(8)	23.1(8)	0.4(7)	0.5(7)	-1.3(7)
C24_4	37.0(11)	22.0(9)	24.2(9)	-1.7(7)	-3.4(8)	7.1(8)
C25_4	31.3(10)	23.5(9)	23.4(9)	0.5(7)	1.7(7)	5.7(8)
C26_4	33.1(10)	21.0(9)	26.7(9)	1.8(7)	4.7(7)	5.3(8)
C27_4	28.8(9)	19.6(9)	20.5(8)	-1.7(7)	3.4(7)	-2.0(7)

Table S21. Anisotropic Displacement Parameters (Å^2×10^3) for 4c$_2$. The Anisotropic displacement factor exponent takes the form: $-2\pi^2[h^2a^{*2}U_{11}+2hka^*b^*U_{12}+…]$.

Atom	U_{11}	U_{22}	U_{33}	U_{23}	U_{13}	U_{12}
O1_5	29.8(7)	27.4(7)	16.9(6)	-2.3(5)	-1.2(5)	8.3(5)
O2_5	38.6(7)	21.2(6)	18.3(6)	-0.8(5)	-5.3(5)	3.3(5)
O3_5	22.8(6)	23.2(6)	22.6(6)	0.3(5)	5.4(5)	-0.4(5)
O4_5	23.1(6)	22.4(6)	25.6(6)	-2.5(5)	3.4(5)	-5.3(5)
O5_5	22.2(6)	18.8(6)	28.9(6)	1.7(5)	-1.9(5)	-3.4(5)
O6_5	23.3(6)	20.8(6)	22.8(6)	0.0(5)	-3.1(5)	1.0(5)
N1_5	20.2(7)	17.0(7)	19.6(7)	-1.8(5)	3.8(5)	-3.1(6)
N2_5	20.7(7)	13.8(7)	20.6(7)	-0.6(5)	3.8(5)	-2.7(5)
N3_5	23.7(7)	18.9(7)	18.3(7)	-3.1(6)	2.2(5)	-1.8(6)
N4_5	20.7(7)	15.8(7)	20.3(7)	1.7(5)	-3.1(5)	-3.8(6)
N5_5	19.8(7)	13.8(7)	21.4(7)	0.1(5)	-2.9(5)	-2.3(5)
N6_5	20.6(7)	15.1(7)	20.3(7)	2.2(5)	-0.1(5)	0.0(6)
C1_5	25.1(9)	24.6(9)	18.6(8)	2.2(7)	0.4(7)	3.6(7)
C2_5	23.2(9)	20.2(9)	21.4(8)	-0.2(7)	0.9(7)	-0.5(7)
C3_5	22.6(9)	21.6(9)	21.1(8)	-0.2(7)	-0.2(7)	-0.5(7)
C4_5	25.6(9)	18.7(9)	21.2(8)	1.3(7)	-1.2(7)	-2.8(7)
C5_5	30.3(10)	19.9(9)	20.5(8)	-1.5(7)	-0.6(7)	-1.0(7)
C6_5	23.0(9)	16.2(8)	19.0(8)	0.4(6)	-1.7(7)	0.9(7)
C7_5	22.5(9)	15.2(8)	18.6(8)	-0.9(6)	-0.4(6)	1.5(7)
C8_5	32.5(10)	25.1(10)	24.9(9)	-10.0(8)	5.3(7)	-5.5(8)
C9_5	21.8(8)	16.1(8)	18.6(8)	-1.7(6)	2.3(6)	-2.9(7)
C10_5	23.6(9)	16.3(8)	20.3(8)	-0.3(6)	-0.5(6)	-2.6(7)
C11_5	22.3(9)	16.0(8)	24.8(9)	-1.4(7)	0.6(7)	3.8(7)
C12_5	25.8(9)	19.2(9)	20.7(8)	3.4(7)	-0.2(7)	1.2(7)
C13_5	22.3(9)	17.0(8)	22.9(8)	-0.4(7)	2.5(7)	-0.5(7)
C14_5	31.5(10)	18.0(9)	22.4(8)	-0.9(7)	-1.3(7)	3.0(7)
C15_5	21.5(9)	17.1(8)	17.8(8)	-1.5(6)	1.8(7)	1.8(7)
C16_5	20.6(9)	15.3(8)	21.7(8)	-0.9(6)	1.6(6)	2.4(7)
C17_5	25.7(9)	19.7(9)	24.0(9)	6.7(7)	1.2(7)	-1.1(7)
C18_5	22.7(9)	15.8(8)	20.8(8)	1.6(6)	-2.1(6)	-3.3(7)
C19_5	22.7(9)	16.2(8)	24.9(9)	-0.1(7)	-1.3(7)	-2.7(7)
C20_5	27.2(9)	18.5(9)	20.4(8)	-2.4(7)	-0.3(7)	-0.8(7)
C21_5	26.0(9)	19.6(9)	20.8(8)	2.1(7)	-3.4(7)	-3.5(7)
C22_5	27.4(9)	16.6(8)	25.0(9)	0.9(7)	-3.3(7)	2.4(7)
C23_5	22.3(9)	17.3(8)	22.8(8)	-0.3(7)	-1.4(7)	-2.1(7)
C24_5	34.4(10)	20.6(9)	23.8(9)	-1.3(7)	-0.8(7)	4.0(8)
C25_5	28.7(10)	22.1(9)	25.3(9)	-0.7(7)	-0.3(7)	4.1(7)
C26_5	35.2(10)	22.7(9)	23.4(9)	1.1(7)	1.9(7)	8.6(8)
C27_5	23.7(9)	20.4(9)	18.9(8)	-2.3(7)	2.1(7)	-0.1(7)

Table S21. Anisotropic Displacement Parameters (Å^2×10^3) for 4c$_2$. The Anisotropic displacement factor exponent takes the form: -2π^2[h^{2}a*^{2}U$_{11}$+2hka*b*U$_{12}$+…].

Atom	U11	U22	U33	U23	U13	U12
O1S_1	45.3(9)	36.1(9)	56.8(9)	-7.9(7)	3.2(7)	17.6(7)
O2S_1	33.0(7)	26.2(7)	39.0(7)	-2.9(6)	5.1(6)	5.1(6)
O3S_1	33.8(8)	29.6(7)	40.8(8)	-1.6(6)	2.2(6)	5.1(6)
C1S_1	25.5(10)	21.3(9)	43.3(11)	-2.9(8)	3.8(8)	-2.5(8)
C2S_1	42.1(12)	28.5(11)	40.7(11)	-5.1(9)	6.2(9)	-0.2(9)
C3S_1	40.0(12)	31.4(11)	48.1(13)	4.3(9)	-4.4(10)	0.3(9)
O1S_2	34.3(8)	31.3(8)	65.1(10)	-1.7(7)	0.0(7)	-12.1(7)
O2S_2	27.8(7)	25.5(7)	50.7(8)	-1.0(6)	-2.0(6)	-4.8(6)
O3S_2	30.0(7)	27.2(7)	50.1(9)	-3.3(6)	1.5(6)	-1.7(6)
C1S_2	22.6(10)	20.2(9)	50.7(12)	-3.6(9)	-1.0(8)	3.1(8)
C2S_2	38.0(12)	30.4(11)	48.9(12)	-4.9(9)	-4.2(9)	0.6(9)
C3S_2	43.1(13)	30.7(11)	53.0(13)	3.4(10)	7.8(10)	6.9(10)
O1S_3	32.6(10)	20.1(9)	30.5(9)	-6.7(8)	0.7(8)	-2.6(8)
O2S_3	28.2(7)	20.5(6)	26.2(6)	-2.5(5)	-3.1(5)	-0.2(5)
O3S_3	28.2(7)	20.5(6)	26.2(6)	-2.5(5)	-3.1(5)	-0.2(5)
C1S_3	27(3)	22(4)	17(3)	0(2)	1.5(14)	-2(3)
C2S_3	29(2)	33.6(14)	24(3)	3.1(14)	-2(2)	-0.9(17)
C3S_3	28(2)	31(2)	26(3)	1.4(16)	-5(2)	-0.6(17)
O1_3	32.6(10)	20.1(9)	30.5(9)	-6.7(8)	0.7(8)	-2.6(8)
O2_3	28.2(7)	20.5(6)	26.2(6)	-2.5(5)	-3.1(5)	-0.2(5)
O3_3	28.2(7)	20.5(6)	26.2(6)	-2.5(5)	-3.1(5)	-0.2(5)
C1_3	27(3)	22(4)	17(3)	0(2)	1.5(14)	-2(3)
C2_3	29(2)	33.6(14)	24(3)	3.1(14)	-2(2)	-0.9(17)
C3_3	28(2)	31(2)	26(3)	1.4(16)	-5(2)	-0.6(17)

Table S22. Bond Lengths for 4c$_2$.

Atom	Atom	Length/Å
O1_4	C1_4	1.429(2)
O1_4	C27_4	1.372(2)
O2_4	C5_4	1.430(2)
O2_4	C21_4	1.367(2)
O3_4	C6_4	1.202(2)
O4_4	C7_4	1.214(2)
O5_4	C16_4	1.210(2)
O6_4	C15_4	1.205(2)
N1_4	N2_4	1.4380(19)
N1_4	C7_4	1.366(2)
N1_5	C9_5	1.435(2)
N2_5	N5_5^2	1.3888(19)
N2_5	C6_5	1.414(2)
N3_5	C6_5	1.369(2)
N3_5	C7_5	1.391(2)
N3_5	C8_5	1.461(2)
N4_5	N5_5	1.4328(19)
N4_5	C16_5	1.372(2)
N4_5	C18_5	1.431(2)
N5_5	C15_5	1.416(2)

Table S22. Bond Lengths for 4c$_2$.

Atom	Atom	Length/Å	Atom	Atom	Length/Å
N1_4	C9_4	1.437(2)	N6_5	C15_5	1.365(2)
N2_4	N5_4[1]	1.3863(19)	N6_5	C16_5	1.390(2)
N2_4	C6_4	1.415(2)	N6_5	C17_5	1.458(2)
N3_4	C6_4	1.369(2)	C1_5	C2_5	1.511(2)
N3_4	C7_4	1.395(2)	C2_5	C3_5	1.526(2)
N3_4	C8_4	1.459(2)	C3_5	C4_5	1.525(2)
N4_4	N5_4	1.4333(19)	C4_5	C5_5	1.505(2)
N4_4	C16_4	1.376(2)	C9_5	C10_5	1.399(2)
N4_4	C18_4	1.428(2)	C9_5	C13_5	1.393(2)
N5_4	C15_4	1.414(2)	C10_5	C11_5	1.388(2)
N6_4	C15_4	1.366(2)	C10_5	C14_5	1.503(2)
N6_4	C16_4	1.394(2)	C11_5	C27_5	1.386(2)
N6_4	C17_4	1.458(2)	C12_5	C13_5	1.391(2)
C1_4	C2_4	1.512(2)	C12_5	C27_5	1.394(2)
C2_4	C3_4	1.519(2)	C13_5	C26_5	1.506(2)
C3_4	C4_4	1.526(2)	C18_5	C19_5	1.399(2)
C4_4	C5_4	1.510(2)	C18_5	C23_5	1.398(2)
C9_4	C10_4	1.401(2)	C19_5	C20_5	1.395(2)
C9_4	C13_4	1.393(2)	C19_5	C24_5	1.503(2)
C10_4	C11_4	1.388(2)	C20_5	C21_5	1.391(2)
C10_4	C14_4	1.504(2)	C21_5	C22_5	1.391(2)
C11_4	C27_4	1.387(2)	C22_5	C23_5	1.388(2)
C12_4	C13_4	1.395(2)	C23_5	C25_5	1.504(2)
C12_4	C27_4	1.395(2)	O1S_1	C1S_1	1.197(2)
C13_4	C26_4	1.508(2)	O2S_1	C1S_1	1.328(2)
C18_4	C19_4	1.395(2)	O2S_1	C2S_1	1.447(2)
C18_4	C23_4	1.399(2)	O3S_1	C1S_1	1.328(2)
C19_4	C20_4	1.397(2)	O3S_1	C3S_1	1.442(2)
C19_4	C24_4	1.507(2)	O1S_2	C1S_2	1.198(2)
C20_4	C21_4	1.391(2)	O2S_2	C1S_2	1.334(2)
C21_4	C22_4	1.393(2)	O2S_2	C2S_2	1.446(2)
C22_4	C23_4	1.383(2)	O3S_2	C1S_2	1.329(2)
C23_4	C25_4	1.507(2)	O3S_2	C3S_2	1.446(3)
O1_5	C1_5	1.429(2)	O1S_3	C1S_3	1.204(14)
O1_5	C27_5	1.3705(19)	O2S_3	C1S_3	1.334(14)
O2_5	C5_5	1.431(2)	O2S_3	C2S_3	1.441(13)
O2_5	C21_5	1.371(2)	O3S_3	C1S_3	1.335(14)
O3_5	C6_5	1.204(2)	O3S_3	C3S_3	1.442(13)
O4_5	C7_5	1.212(2)	O1_3	C1_3	1.202(13)
O5_5	C16_5	1.215(2)	O2_3	C1_3	1.332(13)

Table S22. Bond Lengths for 4c$_2$.

Atom	Atom	Length/Å	Atom	Atom	Length/Å
O6_5	C15_5	1.206(2)	O2_3	C2_3	1.433(16)
N1_5	N2_5	1.4397(19)	O3_3	C1_3	1.336(12)
N1_5	C7_5	1.376(2)	O3_3	C3_3	1.445(15)

[1]-X,1-Y,1-Z; [2]1-X,1-Y,-Z

Table S23. Bond Angles for 4c$_2$.

Atom	Atom	Atom	Angle/°	Atom	Atom	Atom	Angle/°
C27_4	O1_4	C1_4	117.62(13)	C7_5	N3_5	C8_5	124.62(14)
C21_4	O2_4	C5_4	117.21(13)	C16_5	N4_5	N5_5	107.87(13)
C7_4	N1_4	N2_4	107.89(13)	C16_5	N4_5	C18_5	125.82(14)
C7_4	N1_4	C9_4	126.12(14)	C18_5	N4_5	N5_5	119.97(13)
C9_4	N1_4	N2_4	118.55(13)	N2_5[2]	N5_5	N4_5	113.57(12)
N5_4[1]	N2_4	N1_4	113.22(12)	N2_5[2]	N5_5	C15_5	118.98(13)
N5_4[1]	N2_4	C6_4	119.62(13)	C15_5	N5_5	N4_5	106.45(12)
C6_4	N2_4	N1_4	106.79(13)	C15_5	N6_5	C16_5	111.84(13)
C6_4	N3_4	C7_4	111.84(14)	C15_5	N6_5	C17_5	123.47(14)
C6_4	N3_4	C8_4	123.46(14)	C16_5	N6_5	C17_5	124.66(14)
C7_4	N3_4	C8_4	124.70(14)	O1_5	C1_5	C2_5	108.33(14)
C16_4	N4_4	N5_4	107.54(13)	C1_5	C2_5	C3_5	110.68(14)
C16_4	N4_4	C18_4	124.94(14)	C4_5	C3_5	C2_5	113.33(14)
C18_4	N4_4	N5_4	118.91(13)	C5_5	C4_5	C3_5	111.20(14)
N2_4[1]	N5_4	N4_4	114.17(12)	O2_5	C5_5	C4_5	107.87(14)
N2_4[1]	N5_4	C15_4	120.52(13)	O3_5	C6_5	N2_5	125.57(15)
C15_4	N5_4	N4_4	106.86(12)	O3_5	C6_5	N3_5	129.15(15)
C15_4	N6_4	C16_4	112.17(13)	N3_5	C6_5	N2_5	105.25(13)
C15_4	N6_4	C17_4	123.40(14)	O4_5	C7_5	N1_5	127.29(15)
C16_4	N6_4	C17_4	124.37(14)	O4_5	C7_5	N3_5	126.66(15)
O1_4	C1_4	C2_4	108.43(14)	N1_5	C7_5	N3_5	106.04(14)
C1_4	C2_4	C3_4	110.81(14)	C10_5	C9_5	N1_5	117.92(14)
C2_4	C3_4	C4_4	113.39(14)	C13_5	C9_5	N1_5	119.81(14)
C5_4	C4_4	C3_4	110.76(14)	C13_5	C9_5	C10_5	122.21(15)
O2_4	C5_4	C4_4	108.26(14)	C9_5	C10_5	C14_5	121.85(15)
O3_4	C6_4	N2_4	125.59(15)	C11_5	C10_5	C9_5	117.65(15)
O3_4	C6_4	N3_4	129.03(15)	C11_5	C10_5	C14_5	120.48(15)
N3_4	C6_4	N2_4	105.35(13)	C27_5	C11_5	C10_5	121.21(15)
O4_4	C7_4	N1_4	127.45(15)	C13_5	C12_5	C27_5	119.96(16)
O4_4	C7_4	N3_4	126.20(16)	C9_5	C13_5	C26_5	120.71(15)
N1_4	C7_4	N3_4	106.34(14)	C12_5	C13_5	C9_5	118.68(15)

Table S23. Bond Angles for 4c₂.

Atom	Atom	Atom	Angle/°	Atom	Atom	Atom	Angle/°
C10_4	C9_4	N1_4	117.51(15)	C12_5	C13_5	C26_5	120.61(15)
C13_4	C9_4	N1_4	120.02(15)	O6_5	C15_5	N5_5	124.89(15)
C13_4	C9_4	C10_4	122.47(15)	O6_5	C15_5	N6_5	129.74(15)
C9_4	C10_4	C14_4	121.94(15)	N6_5	C15_5	N5_5	105.36(13)
C11_4	C10_4	C9_4	117.63(16)	O5_5	C16_5	N4_5	127.22(15)
C11_4	C10_4	C14_4	120.42(16)	O5_5	C16_5	N6_5	126.51(15)
C27_4	C11_4	C10_4	121.15(16)	N4_5	C16_5	N6_5	106.27(14)
C27_4	C12_4	C13_4	120.00(16)	C19_5	C18_5	N4_5	120.28(15)
C9_4	C13_4	C12_4	118.41(16)	C23_5	C18_5	N4_5	117.41(15)
C9_4	C13_4	C26_4	121.26(15)	C23_5	C18_5	C19_5	122.31(15)
C12_4	C13_4	C26_4	120.33(16)	C18_5	C19_5	C24_5	121.23(15)
O6_4	C15_4	N5_4	125.53(15)	C20_5	C19_5	C18_5	118.34(16)
O6_4	C15_4	N6_4	129.61(15)	C20_5	C19_5	C24_5	120.43(15)
N6_4	C15_4	N5_4	104.85(14)	C21_5	C20_5	C19_5	120.12(16)
O5_4	C16_4	N4_4	127.03(15)	O2_5	C21_5	C20_5	124.67(15)
O5_4	C16_4	N6_4	127.09(15)	O2_5	C21_5	C22_5	114.93(15)
N4_4	C16_4	N6_4	105.88(14)	C20_5	C21_5	C22_5	120.40(15)
C19_4	C18_4	N4_4	120.15(15)	C23_5	C22_5	C21_5	120.97(16)
C19_4	C18_4	C23_4	122.08(15)	C18_5	C23_5	C25_5	122.29(15)
C23_4	C18_4	N4_4	117.77(14)	C22_5	C23_5	C18_5	117.87(15)
C18_4	C19_4	C20_4	118.59(16)	C22_5	C23_5	C25_5	119.84(15)
C18_4	C19_4	C24_4	121.00(15)	O1_5	C27_5	C11_5	115.84(15)
C20_4	C19_4	C24_4	120.40(15)	O1_5	C27_5	C12_5	124.01(15)
C21_4	C20_4	C19_4	119.83(16)	C11_5	C27_5	C12_5	120.14(15)
O2_4	C21_4	C20_4	124.30(15)	C1S_1	O2S_1	C2S_1	114.33(15)
O2_4	C21_4	C22_4	115.19(15)	C1S_1	O3S_1	C3S_1	115.06(15)
C20_4	C21_4	C22_4	120.50(16)	O1S_1	C1S_1	O2S_1	126.05(19)
C23_4	C22_4	C21_4	120.77(16)	O1S_1	C1S_1	O3S_1	126.03(19)
C18_4	C23_4	C25_4	121.27(15)	O2S_1	C1S_1	O3S_1	107.92(15)
C22_4	C23_4	C18_4	118.18(15)	C1S_2	O2S_2	C2S_2	114.62(15)
C22_4	C23_4	C25_4	120.54(15)	C1S_2	O3S_2	C3S_2	114.94(16)
O1_4	C27_4	C11_4	115.52(15)	O1S_2	C1S_2	O2S_2	125.90(19)
O1_4	C27_4	C12_4	124.16(16)	O1S_2	C1S_2	O3S_2	126.69(19)
C11_4	C27_4	C12_4	120.31(16)	O3S_2	C1S_2	O2S_2	107.40(16)
C27_5	O1_5	C1_5	117.16(13)	C1S_3	O2S_3	C2S_3	115.4(11)
C21_5	O2_5	C5_5	118.02(13)	C1S_3	O3S_3	C3S_3	114.3(10)
C7_5	N1_5	N2_5	107.62(12)	O1S_3	C1S_3	O2S_3	125.9(13)
C7_5	N1_5	C9_5	125.13(14)	O1S_3	C1S_3	O3S_3	126.6(12)
C9_5	N1_5	N2_5	117.21(13)	O2S_3	C1S_3	O3S_3	107.1(10)
N5_5[2]	N2_5	N1_5	112.98(12)	C1_3	O2_3	C2_3	114.9(9)

Table S23. Bond Angles for 4c$_2$.

Atom	Atom	Atom	Angle/°	Atom	Atom	Atom	Angle/°
N5_5[2]	N2_5	C6_5	119.49(13)	C1_3	O3_3	C3_3	114.3(8)
C6_5	N2_5	N1_5	106.78(12)	O1_3	C1_3	O2_3	126.5(11)
C6_5	N3_5	C7_5	112.22(13)	O1_3	C1_3	O3_3	126.1(11)
C6_5	N3_5	C8_5	123.15(14)	O2_3	C1_3	O3_3	107.1(9)

[1]-X,1-Y,1-Z; [2]1-X,1-Y,-Z

Table S24. Torsion Angles for 4c$_2$.

A	B	C	D	Angle/°	A	B	C	D	Angle/°
O1_4	C1_4	C2_4	C3_4	-174.18(14)	N2_5	N1_5	C7_5	O4_5	-169.90(16)
O2_4	C21_4	C22_4	C23_4	176.54(15)	N2_5	N1_5	C7_5	N3_5	10.95(17)
N1_4	N2_4	C6_4	O3_4	-170.59(16)	N2_5	N1_5	C9_5	C10_5	-106.00(17)
N1_4	N2_4	C6_4	N3_4	11.43(17)	N2_5	N1_5	C9_5	C13_5	71.24(19)
N1_4	C9_4	C10_4	C11_4	178.37(15)	N2_5[2]	N5_5	C15_5	O6_5	36.9(2)
N1_4	C9_4	C10_4	C14_4	-1.2(2)	N2_5[2]	N5_5	C15_5	N6_5	-143.84(14)
N1_4	C9_4	C13_4	C12_4	-177.36(15)	N4_5	N5_5	C15_5	O6_5	166.69(15)
N1_4	C9_4	C13_4	C26_4	3.4(2)	N4_5	N5_5	C15_5	N6_5	-14.04(16)
N2_4	N1_4	C7_4	O4_4	-170.29(16)	N4_5	C18_5	C19_5	C20_5	-179.87(15)
N2_4	N1_4	C7_4	N3_4	10.58(17)	N4_5	C18_5	C19_5	C24_5	-0.6(2)
N2_4	N1_4	C9_4	C10_4	-105.46(18)	N4_5	C18_5	C23_5	C22_5	179.41(15)
N2_4	N1_4	C9_4	C13_4	73.4(2)	N4_5	C18_5	C23_5	C25_5	-0.7(2)
N2_4[1]	N5_4	C15_4	O6_4	33.5(2)	N5_5[2]	N2_5	C6_5	O3_5	-39.5(2)
N2_4[1]	N5_4	C15_4	N6_4	-147.91(14)	N5_5[2]	N2_5	C6_5	N3_5	142.38(14)
N4_4	N5_4	C15_4	O6_4	165.96(15)	N5_5	N4_5	C16_5	O5_5	170.51(16)
N4_4	N5_4	C15_4	N6_4	-15.44(16)	N5_5	N4_5	C16_5	N6_5	-9.60(16)
N4_4	C18_4	C19_4	C20_4	179.29(15)	N5_5	N4_5	C18_5	C19_5	-76.6(2)
N4_4	C18_4	C19_4	C24_4	-1.5(3)	N5_5	N4_5	C18_5	C23_5	103.29(18)
N4_4	C18_4	C23_4	C22_4	179.75(15)	C1_5	O1_5	C27_5	C11_5	171.93(15)
N4_4	C18_4	C23_4	C25_4	-1.6(2)	C1_5	O1_5	C27_5	C12_5	-7.3(2)
N5_4[1]	N2_4	C6_4	O3_4	-40.4(2)	C1_5	C2_5	C3_5	C4_5	178.01(14)
N5_4[1]	N2_4	C6_4	N3_4	141.61(14)	C2_5	C3_5	C4_5	C5_5	-179.38(14)
N5_4	N4_4	C16_4	O5_4	168.83(16)	C3_5	C4_5	C5_5	O2_5	175.08(14)
N5_4	N4_4	C16_4	N6_4	-10.67(16)	C5_5	O2_5	C21_5	C20_5	-7.6(2)

Table S24. Torsion Angles for 4c₂.

A	B	C	D	Angle/˚
N5_4	N4_4	C18_4	C19_4	-77.6(2)
N5_4	N4_4	C18_4	C23_4	102.52(18)
C1_4	O1_4	C27_4	C11_4	-175.25(15)
C1_4	O1_4	C27_4	C12_4	4.6(3)
C1_4	C2_4	C3_4	C4_4	179.65(15)
C2_4	C3_4	C4_4	C5_4	-179.27(15)
C3_4	C4_4	C5_4	O2_4	-179.42(14)
C5_4	O2_4	C21_4	C20_4	2.7(3)
C5_4	O2_4	C21_4	C22_4	-176.67(16)
C6_4	N3_4	C7_4	O4_4	177.42(17)
C6_4	N3_4	C7_4	N1_4	-3.44(19)
C7_4	N1_4	N2_4	N5_4[1]	-147.65(14)
C7_4	N1_4	N2_4	C6_4	-13.93(17)
C7_4	N1_4	C9_4	C10_4	108.43(19)
C7_4	N1_4	C9_4	C13_4	-72.7(2)
C7_4	N3_4	C6_4	O3_4	176.89(17)
C7_4	N3_4	C6_4	N2_4	-5.22(18)
C8_4	N3_4	C6_4	O3_4	-3.5(3)
C8_4	N3_4	C6_4	N2_4	174.41(15)
C8_4	N3_4	C7_4	O4_4	-2.2(3)
C8_4	N3_4	C7_4	N1_4	176.94(15)
C9_4	N1_4	N2_4	N5_4[1]	60.61(18)
C9_4	N1_4	N2_4	C6_4	-165.67(14)
C9_4	N1_4	C7_4	O4_4	-21.3(3)
C9_4	N1_4	C7_4	N3_4	159.59(15)
C9_4	C10_4	C11_4	C27_4	-1.0(3)
C10_4	C9_4	C13_4	C12_4	1.4(3)
C10_4	C9_4	C13_4	C26_4	-177.82(16)
C10_4	C11_4	C27_4	O1_4	-178.74(16)
C10_4	C11_4	C27_4	C12_4	1.4(3)
C5_5	O2_5	C21_5	C22_5	172.51(15)
C6_5	N3_5	C7_5	O4_5	177.80(16)
C6_5	N3_5	C7_5	N1_5	-3.05(18)
C7_5	N1_5	N2_5	N5_5[2]	-148.28(13)
C7_5	N1_5	N2_5	C6_5	-14.93(17)
C7_5	N1_5	C9_5	C10_5	112.92(18)
C7_5	N1_5	C9_5	C13_5	-69.8(2)
C7_5	N3_5	C6_5	O3_5	175.72(17)
C7_5	N3_5	C6_5	N2_5	-6.22(18)
C8_5	N3_5	C6_5	O3_5	-2.7(3)
C8_5	N3_5	C6_5	N2_5	175.38(15)
C8_5	N3_5	C7_5	O4_5	-3.8(3)
C8_5	N3_5	C7_5	N1_5	175.31(15)
C9_5	N1_5	N2_5	N5_5[2]	64.34(17)
C9_5	N1_5	N2_5	C6_5	-162.31(14)
C9_5	N1_5	C7_5	O4_5	-25.8(3)
C9_5	N1_5	C7_5	N3_5	155.07(15)
C9_5	C10_5	C11_5	C27_5	-1.8(3)
C10_5	C9_5	C13_5	C12_5	2.4(3)
C10_5	C9_5	C13_5	C26_5	-177.73(16)
C10_5	C11_5	C27_5	O1_5	-175.08(15)
C10_5	C11_5	C27_5	C12_5	4.1(3)
C13_5	C9_5	C10_5	C11_5	-1.4(2)
C13_5	C9_5	C10_5	C14_5	-179.70(16)
C13_5	C12_5	C27_5	O1_5	175.98(16)
C13_5	C12_5	C27_5	C11_5	-3.2(3)
C14_5	C10_5	C11_5	C27_5	176.45(16)
C15_5	N6_5	C16_5	O5_5	-179.48(16)
C15_5	N6_5	C16_5	N4_5	0.64(18)
C16_5	N4_5	N5_5	N2_5[2]	147.73(13)

Table S24. Torsion Angles for $4c_2$.

A	B	C	D	Angle/°
C13_4	C9_4	C10_4	C11_4	-0.4(3)
C13_4	C9_4	C10_4	C14_4	179.98(16)
C13_4	C12_4	C27_4	O1_4	179.77(16)
C13_4	C12_4	C27_4	C11_4	-0.3(3)
C14_4	C10_4	C11_4	C27_4	178.62(16)
C15_4	N6_4	C16_4	O5_4	-178.61(16)
C15_4	N6_4	C16_4	N4_4	0.89(18)
C16_4	N4_4	N5_4	N2_4[1]	152.38(13)
C16_4	N4_4	N5_4	C15_4	16.53(16)
C16_4	N4_4	C18_4	C19_4	66.1(2)
C16_4	N4_4	C18_4	C23_4	-113.77(18)
C16_4	N6_4	C15_4	O6_4	-172.20(17)
C16_4	N6_4	C15_4	N5_4	9.28(17)
C17_4	N6_4	C15_4	O6_4	4.9(3)
C17_4	N6_4	C15_4	N5_4	-173.58(14)
C17_4	N6_4	C16_4	O5_4	4.3(3)
C17_4	N6_4	C16_4	N4_4	-176.22(14)
C18_4	N4_4	N5_4	N2_4[1]	-58.21(18)
C18_4	N4_4	N5_4	C15_4	165.94(14)
C18_4	N4_4	C16_4	O5_4	21.7(3)
C18_4	N4_4	C16_4	N6_4	-157.76(14)
C18_4	C19_4	C20_4	C21_4	0.0(3)
C19_4	C18_4	C23_4	C22_4	-0.1(3)
C19_4	C18_4	C23_4	C25_4	178.47(16)
C19_4	C20_4	C21_4	O2_4	-177.50(16)
C19_4	C20_4	C21_4	C22_4	1.9(3)
C20_4	C21_4	C22_4	C23_4	-2.9(3)
C21_4	O2_4	C5_4	C4_4	171.42(14)

A	B	C	D	Angle/°
C16_5	N4_5	N5_5	C15_5	14.89(16)
C16_5	N4_5	C18_5	C19_5	71.9(2)
C16_5	N4_5	C18_5	C23_5	-108.17(19)
C16_5	N6_5	C15_5	O6_5	-172.20(17)
C16_5	N6_5	C15_5	N5_5	8.58(17)
C17_5	N6_5	C15_5	O6_5	6.0(3)
C17_5	N6_5	C15_5	N5_5	-173.19(14)
C17_5	N6_5	C16_5	O5_5	2.3(3)
C17_5	N6_5	C16_5	N4_5	-177.56(14)
C18_5	N4_5	N5_5	N2_5[2]	-58.67(18)
C18_5	N4_5	N5_5	C15_5	168.49(14)
C18_5	N4_5	C16_5	O5_5	18.9(3)
C18_5	N4_5	C16_5	N6_5	-161.24(15)
C18_5	C19_5	C20_5	C21_5	0.2(2)
C19_5	C18_5	C23_5	C22_5	-0.6(3)
C19_5	C18_5	C23_5	C25_5	179.21(16)
C19_5	C20_5	C21_5	O2_5	180.00(16)
C19_5	C20_5	C21_5	C22_5	-0.1(3)
C20_5	C21_5	C22_5	C23_5	-0.4(3)
C21_5	O2_5	C5_5	C4_5	-172.32(14)
C21_5	C22_5	C23_5	C18_5	0.7(3)
C21_5	C22_5	C23_5	C25_5	-179.12(16)
C23_5	C18_5	C19_5	C20_5	0.2(3)
C23_5	C18_5	C19_5	C24_5	179.49(16)
C24_5	C19_5	C20_5	C21_5	-179.12(16)
C27_5	O1_5	C1_5	C2_5	-168.34(14)
C27_5	C12_5	C13_5	C9_5	0.0(3)
C27_5	C12_5	C13_5	C26_5	-179.92(16)

Table S24. Torsion Angles for 4c$_2$.

A	B	C	D	Angle/°	A	B	C	D	Angle/°
C21_4	C22_4	C23_4	C18_4	2.0(3)	C2S_1	O2S_1	C1S_1	O1S_1	-1.3(3)
C21_4	C22_4	C23_4	C25_4	-176.64(16)	C2S_1	O2S_1	C1S_1	O3S_1	179.21(15)
C23_4	C18_4	C19_4	C20_4	-0.8(3)	C3S_1	O3S_1	C1S_1	O1S_1	-1.2(3)
C23_4	C18_4	C19_4	C24_4	178.38(16)	C3S_1	O3S_1	C1S_1	O2S_1	178.35(15)
C24_4	C19_4	C20_4	C21_4	-179.26(17)	C2S_2	O2S_2	C1S_2	O1S_2	3.0(3)
C27_4	O1_4	C1_4	C2_4	175.30(14)	C2S_2	O2S_2	C1S_2	O3S_2	-177.72(15)
C27_4	C12_4	C13_4	C9_4	-1.0(3)	C3S_2	O3S_2	C1S_2	O1S_2	2.0(3)
C27_4	C12_4	C13_4	C26_4	178.23(16)	C3S_2	O3S_2	C1S_2	O2S_2	-177.33(15)
O1_5	C1_5	C2_5	C3_5	-178.85(14)	C2S_3	O2S_3	C1S_3	O1S_3	10(9)
O2_5	C21_5	C22_5	C23_5	179.53(15)	C2S_3	O2S_3	C1S_3	O3S_3	-176(3)
N1_5	N2_5	C6_5	O3_5	-169.20(16)	C3S_3	O3S_3	C1S_3	O1S_3	0(9)
N1_5	N2_5	C6_5	N3_5	12.65(16)	C3S_3	O3S_3	C1S_3	O2S_3	-173(3)
N1_5	C9_5	C10_5	C11_5	175.71(15)	C2_3	O2_3	C1_3	O1_3	-4(8)
N1_5	C9_5	C10_5	C14_5	-2.5(2)	C2_3	O2_3	C1_3	O3_3	171(3)
N1_5	C9_5	C13_5	C12_5	-174.76(15)	C3_3	O3_3	C1_3	O1_3	-8(8)
N1_5	C9_5	C13_5	C26_5	5.2(2)	C3_3	O3_3	C1_3	O2_3	177(3)

[1]-X,1-Y,1-Z; [2]1-X,1-Y,-Z

Table S25. Hydrogen Atom Coordinates (Å×10^4) and Isotropic Displacement Parameters (Å^2×10^3) for 4c$_2$.

Atom	*x*	*y*	*z*	U(eq)
H1A_4	2866.36	5506.92	5438.55	27
H1B_4	1441.65	4817.7	5443.03	27
H2A_4	2663.91	3739.34	5174.3	27
H2B_4	4174.62	4347.35	5186.42	27
H3A_4	2939.33	5599	4967.43	28
H3B_4	1436.63	4987.84	4954.2	28
H4A_4	4148.08	4489.24	4693.43	27
H4B_4	2631.76	3887.44	4678.26	27
H5A_4	1397.07	5176.22	4474.79	31
H5B_4	2924.26	5772.08	4487.99	31
H8A_4	1459.09	7610.91	7186.26	41
H8B_4	3224.88	7533.79	7139.3	41

Table S25. Hydrogen Atom Coordinates (Å×10^4) and Isotropic Displacement Parameters (Å^2×10^3) for 4c$_2$.

Atom	*x*	*y*	*z*	U(eq)
H8C_4	2449.1	6900.26	7355.73	41
H11_4	4335.86	3703.34	6046.97	29
H12_4	1526.93	5768.92	5806.58	28
H14A_4	4528.86	4385.44	6697.88	38
H14B_4	5242.67	3690.08	6489.45	38
H14C_4	3660.22	3445.56	6616.11	38
H17A_4	25.92	7434.13	2630.02	35
H17B_4	862.92	8090.01	2836.54	35
H17C_4	-928.46	8044.01	2829.59	35
H20_4	1234.41	6174.98	4149.35	28
H22_4	3555.53	4079.13	3803.42	27
H24A_4	568.95	7551.53	3704.8	42
H24B_4	-590.81	7071.11	3898.41	42
H24C_4	-755.06	6904.5	3594.15	42
H25A_4	1997.04	3825.01	3281.33	39
H25B_4	3733.55	4026.77	3337.1	39
H25C_4	2775.02	4742.9	3164.06	39
H26A_4	112.5	6414.47	6380.64	40
H26B_4	410.84	6818.38	6097.46	40
H26C_4	1513.99	7090.63	6330.6	40
H1A_5	8487.09	5831.56	438.91	27
H1B_5	7044.09	5224.23	517.54	27
H2A_5	7942.73	3937.65	272.14	26
H2B_5	9403.7	4529.68	196.96	26
H3A_5	6405.39	5025.03	45.96	26
H3B_5	7842.8	5644.96	-22.92	26
H4A_5	8771.65	4394.59	-282.93	26
H4B_5	7320.4	3781.55	-215.58	26
H5A_5	5767.03	4856.99	-432.59	28
H5B_5	7167.51	5539.87	-483.79	28
H8A_5	6391.11	7911.64	2206.06	41
H8B_5	8178.41	7900.8	2183.88	41
H8C_5	7361.45	7216.11	2383.47	41
H11_5	10011.29	4126.96	1088.67	25
H12_5	7387.59	6263.1	835.33	26
H14A_5	10015.82	4719.24	1740.97	36
H14B_5	10650.55	3979.99	1536.22	36
H14C_5	9015.72	3837.72	1654.71	36
H17A_5	5174.94	6997.58	-2355.04	35

Table S25. Hydrogen Atom Coordinates (Å×10^4) and Isotropic Displacement Parameters (Å^2×10^3) for 4c$_2$.

Atom	*x*	*y*	*z*	U(eq)
H17B_5	5944.67	7653	-2140.84	35
H17C_5	4162.64	7658.95	-2177.4	35
H20_5	5397.23	5787.82	-805.19	26
H22_5	7906.7	3711.12	-1117.64	28
H24A_5	4869.02	7127.3	-1304.3	39
H24B_5	3900.19	6781.83	-1063.69	39
H24C_5	3476.18	6435.67	-1348.83	39
H25A_5	6744.94	3431.61	-1651.73	38
H25B_5	8419.78	3744.47	-1580.27	38
H25C_5	7415.66	4384.91	-1767.55	38
H26A_5	5703.51	6829.74	1386.83	41
H26B_5	6225.92	7306.61	1122.71	41
H26C_5	7161.47	7484.65	1382.78	41
H2SA_1	-1648.13	8162.76	4287.57	56
H2SB_1	-1261.41	7086.85	4349.79	56
H2SC_1	54.6	7848.72	4333.93	56
H3SA_1	1425.74	7180.23	5201.47	60
H3SB_1	38.66	6482.48	5240.3	60
H3SC_1	182.45	7411.57	5413.05	60
H2SA_2	5899.54	3131.58	4190.25	59
H2SB_2	4248.41	2927.15	4294.59	59
H2SC_2	5421.65	2083.01	4266.03	59
H3SA_2	5320.23	1514.83	5189.54	63
H3SB_2	3930.59	2226.13	5190.52	63
H3SC_2	5402.08	2459.71	5356.87	63
H2SA_3	5369.78	4942.94	2545.05	44
H2SB_3	4648.61	4378.66	2780.63	44
H2SC_3	5512.39	5347.77	2832.28	44
H3SA_3	481.99	5447.28	2136.94	42
H3SB_3	-712.44	5805.24	2343.64	42
H3SC_3	-57.47	4759.36	2362.26	42
H2A_3	5318.66	5649.85	2611.71	44
H2B_3	5874.02	4607.74	2679.4	44
H2C_3	4710.13	5130.18	2863.94	44
H3A_3	543.16	5914.96	2193.11	42
H3B_3	-113.9	5520.66	2458.37	42
H3C_3	-377.55	4949.13	2195.93	42

Table S26. Atomic Occupancy for 4c₂.

Atom	*Occupancy*	Atom	*Occupancy*	Atom	*Occupancy*
O1S_3	0.4628(12)	O2S_3	0.4628(12)	O3S_3	0.4628(12)
C1S_3	0.4628(12)	C2S_3	0.4628(12)	H2SA_3	0.4628(12)
H2SB_3	0.4628(12)	H2SC_3	0.4628(12)	C3S_3	0.4628(12)
H3SA_3	0.4628(12)	H3SB_3	0.4628(12)	H3SC_3	0.4628(12)
O1_3	0.5372(12)	O2_3	0.5372(12)	O3_3	0.5372(12)
C1_3	0.5372(12)	C2_3	0.5372(12)	H2A_3	0.5372(12)
H2B_3	0.5372(12)	H2C_3	0.5372(12)	C3_3	0.5372(12)
H3A_3	0.5372(12)	H3B_3	0.5372(12)	H3C_3	0.5372(12)

Experimental

Single crystals were recrystallized from a solution of Compound **4c₂** (GB2022) dissolved in dimethylcarbonate. A suitable crystal with dimensions 0.27 × 0.21 × 0.10 mm^3 was selected and mounted on a loop with paratone oil on a XtaLAB Synergy, Dualflex, HyPix diffractometer. The crystal was kept at 100 K during data collection. Using *Olex2* [1], the structure was solved with the *olex2.solve* [2] structure solution program and refined with *SHELXL* [3] via full matrix least squares minimization on F^2.

Crystal structure determination of 4c₂

Crystal Data for $C_{63}H_{82}N_{12}O_{21}$ (M =1343.40 g/mol): monoclinic, space group $P2_1/c$ (no. 14), a = 8.92534(8) Å, b = 14.21670(12) Å, c = 51.8332(4) Å, β = 90.4433(8)°, V = 6576.87(9) $Å^3$, Z = 4, T = 100.0(9) K, μ(Cu Kα) = 0.862 mm^{-1}, $Dcalc$ = 1.357 g/cm^3, 58388 reflections measured (6.446° ≤ 2Θ ≤ 146.38°), 12479 unique (R_{int} = 0.0609, R_{sigma} = 0.0455) which were used in all calculations. The final R_1 was 0.0448 (I > 2σ(I)) and wR_2 was 0.1150 (all data).

Refinement model description

Number of restraints - 30, number of constraints - unknown.

Details:

```
1. Uiso/Uaniso restraints and constraints
Uanis(C3S_3) = Uanis(C3_3)
Uanis(O2_3) = Uanis(O3_3)
Uanis(C1S_3) = Uanis(C1_3)
Uanis(C2S_3) = Uanis(C2_3)
Uanis(O1S_3) = Uanis(O1_3)
Uanis(O3S_3) = Uanis(O3_3)
Uanis(O2_3) = Uanis(O2S_3)
2. Same fragment restrains
3. Others
 Sof(O1S)=Sof(O2S)=Sof(O3S)=Sof(C1S)=Sof(C2S)=Sof(H2SA)=Sof(H2SB)=Sof(H2SC)=
 Sof(C3S)=Sof(H3SA)=Sof(H3SB)=Sof(H3SC)=1-FVAR(1)
 Sof(O1)=Sof(O2)=Sof(O3)=Sof(C1)=Sof(C2)=Sof(H2A)=Sof(H2B)=Sof(H2C)=Sof(C3)=
 Sof(H3A)=Sof(H3B)=Sof(H3C)=FVAR(1)
4.a Secondary CH2 refined with riding coordinates:
 C1(H1A,H1B), C2(H2A,H2B), C3(H3A,H3B), C4(H4A,H4B), C5(H5A,H5B), C1(H1A,H1B),
 C2(H2A,H2B), C3(H3A,H3B), C4(H4A,H4B), C5(H5A,H5B)
4.b Aromatic/amide H refined with riding coordinates:
 C11(H11), C12(H12), C20(H20), C22(H22), C11(H11), C12(H12), C20(H20), C22(H22)
4.c Idealised Me refined as rotating group:
 C8(H8A,H8B,H8C), C14(H14A,H14B,H14C), C17(H17A,H17B,H17C), C24(H24A,H24B,
 H24C), C25(H25A,H25B,H25C), C26(H26A,H26B,H26C), C8(H8A,H8B,H8C), C14(H14A,
 H14B,H14C), C17(H17A,H17B,H17C), C24(H24A,H24B,H24C), C25(H25A,H25B,H25C),
 C26(H26A,H26B,H26C), C2S(H2SA,H2SB,H2SC), C3S(H3SA,H3SB,H3SC), C2S(H2SA,H2SB,
 H2SC), C3S(H3SA,H3SB,H3SC), C2S(H2SA,H2SB,H2SC), C3S(H3SA,H3SB,H3SC), C2(H2A,
 H2B,H2C), C3(H3A,H3B,H3C)
```

This report has been created with Olex2, compiled on 2023.02.24 svn.rf166f9f3 for OlexSys. Please let us know if there are any errors or if you would like to have additional features.

References

1. Dolomanov, O. V.; Bourhis, L. J.; Gildea, R. J.; Howard, J. A. K.; Puschmann, H. *J.* OLEX2: A Complete Structure Solution, Refinement and Analysis Program. *Appl. Cryst.* **2009**, *42*, 339-341. https://doi.org/10.1107/S0021889808042726

2. Bourhis, L. J.; Dolomanov, O. V.; Gildea, R. J.; Howard, J. A. K.; Puschmann, H. The Anatomy of a Comprehensive Constrained, Restrained Refinement Program for the Modern Computing Environment – Olex2 Dissected. *Acta Cryst.* **2015**, *A71*, 59-75. https://doi.org/10.1107/S2053273314022207

3. Sheldrick, G. M. SHELXT- Integrated Space-Group and Crystal-Structure Determination. *Acta Cryst.* **2015**, *C71*, 3-8. https://doi.org/10.1107/S2053273314026370

4. Macrae, C. F.; Sovago, I.; Cottrell, S. J.; Galek, P. T. A.; McCabe, P.; Pidcock, E.; Platings, M.; Shields, G. P.; Stevens, J. S.; Towler, M.; Wood, P. A. Mercury 4.0: From Visualization to Analysis, Design and Prediction. *J. Appl. Cryst.* **2020**, *53*, 226-235. https://doi.org/10.1107/S1600576719014092
